# Clinical laboratory parameters and fatality of Severe fever with thrombocytopenia syndrome patients: A systematic review and meta-analysis

**DOI:** 10.1371/journal.pntd.0010489

**Published:** 2022-06-17

**Authors:** Yao Wang, Zexuan Song, Xuemin Wei, Haowen Yuan, Xiaoying Xu, Hao Liang, Hongling Wen

**Affiliations:** 1 Department of Microbiological Laboratory Technology, School of Public Health, Cheeloo College of Medicine, Shandong University, Jinan, China; 2 National Tuberculosis Reference Laboratory, Chinese Center for Disease Control and Prevention, Beijing, China; NIAID Integrated Research Facility, UNITED STATES

## Abstract

**Background:**

Severe fever with thrombocytopenia syndrome (SFTS) is an emerging tick-borne infectious disease with high case fatality rate. Unfortunately, no vaccine or antiviral specifically targeting SFTS virus (SFTSV) are available for the time being. Our objective was to investigate the association between clinical laboratory parameters and fatality of SFTS patients.

**Methods:**

The systematic review was conducted in accordance with The Preferred Reporting Items for Systematic Reviews and Meta-Analyses 2020 guidelines. We searched (from inception to 24th February 2022) Web of Science, PubMed, National Knowledge Infrastructure databases and Wan Fang Data for relevant researchers on SFTS. Studies were eligible if they reported on laboratory parameters of SFTS patients and were stratified by clinical outcomes. A modified version of Newcastle-Ottawa scale was used to evaluate the quality of included studies. Standardized mean difference (SMD) was used to evaluate the association between laboratory parameters and outcomes. The between-study heterogeneity was evaluated quantitatively by standard Chi-square and the index of heterogeneity (*I*^2^). Heterogeneity was explored by subgroup and sensitivity analyses, and univariable meta-regression. Publication bias was determined using funnel plots and Egger’s test.

**Results:**

We identified 34 relevant studies, with over 3300 participants across three countries. The following factors were strongly (SMD>1 or SMD<-0.5) and significantly (*P*<0.05) associated mortality: thrombin time (TT) (SMD = 1.53), viral load (SMD = 1.47), activated partial-thromboplastin time (APTT) (SMD = 1.37), aspartate aminotransferase (AST) (SMD = 1.19), lactate dehydrogenase (LDH) (SMD = 1.13), platelet count (PLT) (SMD = -0.47), monocyte percentage (MON%) (SMD = -0.47), lymphocyte percentage (LYM%) (SMD = -0.46) and albumin (ALB) (SMD = -0.43). Alanine aminotransferase, AST, creatin phosphokinase, LDH, PLT, partial-thromboplastin time and viral load contributed to the risk of dying of SFTS patients in each subgroup analyses. Sensitivity analysis demonstrated that the results above were robust.

**Conclusions/significance:**

The abnormal levels of viral load, PLT, coagulation function and liver function, significantly increase the risk of SFTS mortality, suggesting that SFTS patients with above symptoms call for special concern.

## Author summary

Severe fever with thrombocytopenia syndrome (SFTS) is an emerging infectious disease with high case-fatality rate and lack of vaccines, calling for an urgent need to identify the risk factors of mortality. Compared to the previous studies concentrating on clinical manifestations diagnosed partly relying on empirical subjective assessment, our study aimed to systematically analyzed the association between SFTS patients’ outcomes and clinical laboratory parameters. What’s more, no consistent conclusion derived because of sample sizes with enormous differences. In this systematic review, we searched the medical literature and found 34 studies evaluating associations between laboratory factors and risk of dying among SFTS patients. These studies described 3388 SFTS patients of whom 739 (21.81%) died. SFTS patients were at increased risk of dying if they had abnormal strongly levels of viral load, PLT, coagulation function and liver function. Therefore, the patients with above-mentioned situations should be monitored and cured carefully.

## Introduction

Severe fever with thrombocytopenia syndrome (SFTS) is an emerging tick-borne infectious disease characterized by fever, leukopenia, thrombocytopenia, central nervous system symptoms and even multiple organ dysfunctions, with high case fatality rate of 12–50% [1–3]. The pathogen responsible for SFTS was identified as SFTS virus (SFTSV) which is a tick-borne virus in the genus *Bandavirus* in the family *Phenuiviridae*, order *Bunyavirales* [4]. The genome of SFTSV is a single-stranded negative sense RNA virus and comprises three segments (S, M, L) [4].

SFTS was first described in China in 2009, and subsequently reported in South Korea, Japan and the United States. Moreover, an increasing number of novel SFTSV-like viruses continue to be isolated from wild animals and tick vectors around the world with spatial expansion of ticks due to environmental changes, indicating a broader global distribution and raising serious concerns about potentially growing epidemics of SFTSV across continents [1–2]. SFTS has been listed doubtlessly as one of 10 priority diseases by the World Health Organization since 2017 [5]. Unfortunately, no vaccine or antiviral specifically targeting SFTSV is available for the time being, suggesting that capturing the risk factors contributing to fatality is urgent.

Some published meta-analysis studies had described the potential risk factors contributing to fatality of SFTS disease. Liu MM et al. found that old age, central nervous system manifestations, bleeding tendency, elevated serum enzymes and high vial load were risk factors for fatality among SFTS patients [6]. Wang X et al. found that there were some significant differences between the nonfatal and fatal groups, such as headache, fatigue, diarrhea, vomiting and arrhythmia [7]. Dualis et al. emphasized the importance of delay in hospital admission, high viral load, older age and presence of comorbid or complications on risk of dying of SFTS cases [8]. Routine laboratory parameters mentioned in another meta-analysis paper were just used to evaluate the severity of SFTS patients [9]. In brief, the previous meta-analysis studies associating with risk factors related to mortality were concentrated on clinical manifestations diagnosed partly relying on empirical subjective assessment, and they lacked effective methods to deal with significant heterogeneity justifying the robustness of results [6–9]. Though some researches had demonstrated the clinical laboratory parameters contributing to fatality of SFTS disease, the results were inconsistent. In fact, clinical laboratory parameters, the relatively objective factors, are more suitable for future application of predictors for outcome of SFTS disease.

We therefore set out to conduct a systematic review to identify key clinical laboratory parameters associated with mortality among SFTS patients and used several methods to justify the robustness of results. To our knowledge, this is the first meta-analysis exclusively concerning the laboratory indexes to clarify the association between clinical laboratory tests and SFTS patients’ outcomes.

## Materials and methods

### Protocol and registration

This protocol follows the recommendations established by the Preferred Reporting Items for Systematic Reviews and Meta-Analyses (PRISMA) statement, and it has been reported in the International Prospective Register of Systematic Reviews (PROSPERO) database (Registration ID: CRD42021283767). The completed PRISMA checklist is available in S1 Text.

### Eligibility criteria

We aimed to include studies on SFTS patients from all over the world, with laboratory-confirmed diagnosis and treated in hospitals or other health care structures. We included all articles that fulfilled the following inclusion criteria: i) Patients included must meet one or more of the following criteria: (1) isolated the virus from serum samples, (2) a 4-fold or greater increase of antibody titers was detected between a paired serum samples of the patient collected from the acute and convalescent phases of infection, (3) SFTSV RNA was detected from the patient’s serum by reverse-transcriptase PCR (RT-PCR). ii) The clinical outcomes were categorized by “non-fatal” verse “fatal”, and the article must include the total fatal and non-fatal number, and the fatal and non-fatal number associated with various clinical laboratory parameters. iii) The study was published in English or Chinese.

The exclusion criteria: duplicate publications, sample size less than 20 cases, conference abstracts, letters to editor, review articles, commentary, overlapping data sets, animal experiments, no English title.

### Information sources, search strategy, and study selection

According to the PRISMA guidelines, we did a systematic literature review from four databases (PubMed, Web of Science, National Knowledge Infrastructure databases (CNKI), Wan Fang Data) covering literature until February 24, 2022. The search strategy combined terms indicating the disease (such as “severe fever with thrombocytopenia syndrome”, “SFTS”, “bunyavirus” and “Dabie bandavirus”) with terms indicating the outcomes of SFTS cases (such as “outcome”, “fatal”, “death” and “deceased”). Exact search terms are provided in S1 Table.

To ensure literature saturation, references from included studies and excluded review articles were hand searched to check if the automatic search had missed any important investigations.

Two authors (Y.W and Z.X.S) each separately screened the search result titles and abstracts and then reviewed eligible full texts of the retained studies. A third author (H.L.W) resolved disagreement between the two reviewers regarding eligibility of a study.

### Data items and data collection process

The core information was the strength of association between laboratory parameters and mortality. We extracted the information of clinical laboratory parameters at baseline. The following information was extracted from every eligible article: first author, publication year, region, number of patients. Meanwhile clinical laboratory parameters were extracted, including viral load (log_10_), platelet count (PLT), lymphocyte percentage (LYM%), monocyte (MON), monocyte percentage (MON%), hemoglobin (Hgb), neutrophil percentage (NEU%), white blood cell (WBC), lymphocyte (LYM), red blood cell (RBC), neutrophil (NEU), activated partial-thromboplastin time (APTT), partial-thromboplastin time (PT), thrombin time (TT), fibrinogen (FIB), gamma-glutamyl transferase (GGT), alanine aminotransferase (ALT), creatin phosphokinase (AST), alkaline phosphatase (ALP), total bilirubin (TB), albumin (ALB), blood urea nitrogen (BUN), serum creatinine (sCr), creatin phosphokinase (CK), creatinine kinase myocardial b fraction (CK-MB), lactate dehydrogenase (LDH), C-reactive protein (CRP), K (potassium), D-dimer (D-D) and Na (sodium). Two authors (H.W.Y and X.Y.X) independently extracted and recorded data from selected studies. Disagreements were resolved by a third author (W.Y.).

### Quality assessment

Two authors (Y.W and X.Y.X) each separately evaluated the quality of each included study using a modified version of the Newcastle-Ottawa Quality Assessment Scale (NOS) [10], and A third investigator (H.L.W) was consulted when disagreements arose. The NOS tool has scores ranging from 0 to 9. In accordance with the protocol, the NOS scores were divided into low quality (scores 1–4), intermediate quality (scores 5–7), and high-quality (scores 8–9) (S2 Table).

### Data analysis

For pooling of means of numerical variables, we computed missing means and standard deviations (SDs) from medians, ranges (minimum to maximum), and interquartile ranges (IQRs) using the methods proposed by Hozo et al. [11] and Wan et al. [12]. The association between clinical laboratory parameters and outcomes was estimated by calculating standardized mean difference (SMD) and 95% confidence intervals (*CI*).

The heterogeneity between studies was assessed using the standard Chi-square and index of heterogeneity squared (*I*^*2*^) statistic with value of >50% denoting high level of heterogeneity. Fixed effect meta-analysis was used if no heterogeneity was found (*I*^*2*^<50%), otherwise, the random effect model was used. However, fixed effect model was considered irrespective of the degree of heterogeneity if the number of studies included in analysis was small (<five) [13].

In an attempt to account for high heterogeneity, we performed subgroup analyses for factors which were reported in at least five studies and meta-regression analyses for factors which were reported in at least ten studies [14]. We conducted subgroup and meta-regression analyses according to the study types (retrospective and prospective), mean age of the participants (<60, 60–65, >65), study sites (one, two or more) and sample sizes (<50, 50–100, >100). Because the numbers and characteristics of laboratory parameters were different, subgroup analyses and meta-regression of some parameters did not include the above variables simultaneously. Furthermore, the sensitivity analysis was performed by doing leave-one-out analysis to assess the influence of individual study.

For each laboratory parameter reported in at least five studies, funnel plots were utilized to evaluate potential publication bias and asymmetry was assessed by Egger’s test (*P*<0.05 was considered statistically significant). The statistical analysis was performed via STATA version 11.0 (StataCorp LP, College Station, Texas, USA) and Review Manager (RevMan version 5.3, Nordic Cochrane Centre, Copenhagen, Denmark).

## Results

### Characteristics of the selected studies

**Fig 1:**
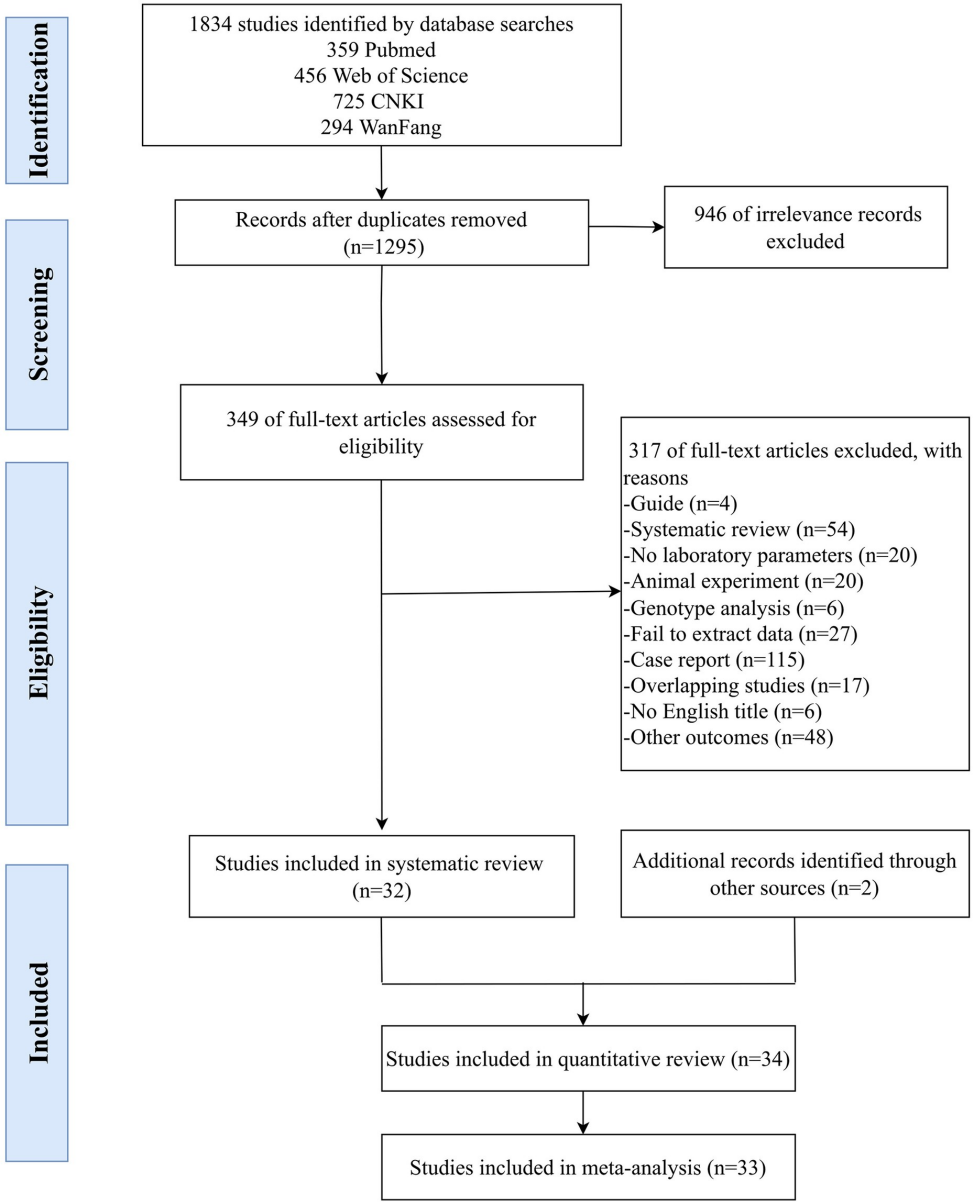
PRISMA diagram of the systematic review and meta-analysis.

The flow chart of literature searching and selection was shown in Fig 1. A total of 1834 studies were identified by database searches and 2 studies were identified from reference lists, of which 539 duplicates and 946 irrelevant studies (solitary fibrous tumors) were removed. Then 273 articles were excluded due to case reports / animal experiments / guides / no laboratory parameter / systematic reviews / genotypes analysis / no English titles and other outcomes after review of the titles and abstracts. After carefully reviewing the full text and data of the remaining 76 articles, 42 ineligible records were excluded due to overlapping data or failing to extract data. Finally, 34 studies containing 3,388 SFTS patients (2,649 survival cases and 739 fatality cases) were included in the final analysis.

The basic characteristics and data extraction from these included studies were shown in Table 1. 23.53% (8/34) of them enrolled SFTS patients prospectively. All studies were conducted in Western Pacific Region, of which China, South Korea and Japan accounted for 85.29% (29/34), 8.82% (3/34) and 5.88% (2/34), respectively. 67.65% (23/34) of studies enrolled participants just from one hospital. Patient inclusion criteria varied across studies: most studies included all SFTS patients [16,18–23,25,28–30,32–35,37,40–48], whereas others had strict enrolment criteria. For example, SFTS cases were excluded if they had a history of serious chronic diseases or they were coinfected by other viruses in some studies [15,17,24,26,36,39]. Besides, some studies focused on specific SFTS patients, such as critical ill patients [27,38]. The included study sample size ranged from 23 [31,38] to 429 [36].

**Table 1:** Characteristics of included studies in the review.

Author and year	Study type	WHO region	Country	Region	Study sites	Year of data collection	Fatal number	Non-fatal number	Mean age (years)
Chen GS et al., 2017 [15]	Retrospective	WPR	China	Anhui	One	2014.1–2015.12	2	40	58.9
Chu SS et al., 2019 [16]	Retrospective	WPR	China	Zhejiang	One	2012.5–2018.5	7	33	65.7
Gui Y et al., 2021 [17]	Prospective	WPR	China	Anhui	One	2019.1–2021.6	36	91	62.3
Han CX et al., 2019 [18]	Retrospective	WPR	China	Liaoning	One	2012–2017	16	56	63.61
Hou HH et al., 2021 [19]	Retrospective	WPR	China	Liaoning	Multi	2018.1–2020.7	33	79	65.3
Jia B et al., 2017 [20]	Retrospective	WPR	China	Jiangsu	One	2010.10–2017.7	33	109	58.3
Kato H et al., 2016 [21]	Retrospective	WPR	Japan	NR	Multi	2013.3–2014.9	15	34	75.52
Kwon JS et al., 2021 [22]	Prospective	WPR	South Korea	NR	Multi	2015.6–2020.10	7	37	63.8
Li J et al., 2014 [23]	Prospective	WPR	China	Jiangsu	One	2011.5–2013.7	4	29	59.5
Li MM et al., 2018 [24]	Retrospective	WPR	China	Hubei	One	2015.5–2016.9	12	30	58.4
Liu JY al., 2018 [25]	Retrospective	WPR	China	Zhejiang	One	2011.5–2016.10	16	40	64
Liu W et al., 2013 [26]	Retrospective	WPR	China	Henan	One	2011–2012	54	257	60.3
Nie Q et al., 2020 [27]	Retrospective	WPR	China	Multi	Multi	2014.1–2019.12	50	66	63
Peng C et al., 2016 [28]	Retrospective	WPR	China	Hubei	One	2014.4–2014.8	9	44	53.4
Sheng QY et al., 2019 [29]	Retrospective	WPR	China	Zhejiang	Multi	2011.8–2017.12	10	15	63.7
Shin J et al., 2015 [30]	Retrospective	WPR	South Korea	National	Multi	2013.5–2013.11	16	19	66.2
Suemori K et al., 2020 [31]	Prospective	WPR	Japan	Western	Multi	2016.4–2016.12 & 2017.9–2018.7	4	19	72.4
Sun L et al., 2014 [32]	Prospective	WPR	China	Hubei	One	2012.5–2012.9	3	31	54.8
Sun Y et al., 2012 [33]	Prospective	WPR	China	Multi	Multi	2010	15	44	61.02
Tan QL et al., 2016 [34]	Retrospective	WPR	China	Zhejiang	One	2013–2014	8	24	66.25
Wang F et al., 2020 [35]	Retrospective	WPR	China	Jiangsu	One	2013.01–2019.01	16	35	74.02
Wang L et al., 2019 [36]	Prospective	WPR	China	Multi	Multi	2011.4–2018.12	69	360	60.8
Wang L et al., 2020 [37]	Retrospective	WPR	China	Shandong	One	2013.5–2017.7	87	234	63.8
Xiao LY et al., 2020 [38]	Retrospective	WPR	China	Jiangsu	One	2014.8–2019.9	8	15	58.3
Xiong et al., 2016 [39]	Retrospective	WPR	China	Hubei	One	2015.3–2015.11	34	145	58.1
Yang B et al., 2017 [40]	Retrospective	WPR	China	Shandong	One	2011.6–2014.10	31	92	59.5
Yang M et al., 2018 [41]	Retrospective	WPR	China	Anhui	One	2012.1–2016.9	20	49	62.81
Yin M et al., 2020 [42]	Retrospective	WPR	China	Anhui	One	2016.1–2019.12	13	46	65.22
Yoo JR et al., 2021 [43]	Retrospective	WPR	South Korea	Jeju Island	One	2013.4–2019.12	7	47	62.8
You EQ et al., 2021 [44]	Retrospective	WPR	China	Anhui	One	2014–2018	30	198	63.9
Zeng QQ et al., 2017 [45]	Retrospective	WPR	China	Zhejiang	One	2011.6–2016.6	17	90	65.5
Zhang YZ et al., 2012 [46]	Prospective	WPR	China	Hubei	Two	2010.4–2010.10	8	41	54.4
Zhao H et al., 2020 [47]	Retrospective	WPR	China	Shandong	Multi	2014–2017	11	36	NA
Zhou SJ et al., 2021 [48]	Retrospective	WPR	China	Anhui	One	2015.5–2020.8	38	164	62.8

WPR: Western Pacific Region. NR: not recognized

A widely variety of potential laboratory parameters were evaluated in S3 Table. The factors that were assessed most frequently (in at least ten articles) were viral load, PLT, NEU, Hgb, WBC, APTT, PT, ALT, AST, ALB, BUN, sCr, LDH, CK and CK-MB. Only five laboratory parameters were assessed in three or four studies.

### Quality of included studies

S4 Table provides the overall quality score for each study included in the review. No study met the threshold for high quality. 33 studies were found to be of moderate quality, and the remaining 1 study had poor quality particularly because there was a poor description of the study population (how and why participants were sampled, adequacy of sample size) and impact of bias. Finally, the poor-quality study was excluded from meta-analysis.

### The relationship between viral load and fatality of SFTS patients

The fixed-effect model showed that high level of viral load was associated with fatal SFTS disease (SMD = 1.47, 95%*CI*: 1.02~1.92; *P*<0.001). Substantial heterogeneity was noticed with *I*^2^ = 79% and *P* < 0.001 (S2 Text).

### The relationship between blood routine tests and fatality of SFTS patients

Blood routine tests showed that, compared with non-fatal SFTS patients, fatal SFTS patients had significantly reduced levels of PLT (SMD = -0.47, 95%*CI*: -0.61~-0.32; *P*<0.001), LYM% (SMD = -0.46, 95%*CI*: -0.61~-0.31; *P*<0.001) and MON% (SMD = -0.47, 95%*CI*: -0.65~-0.30; *P*<0.001), but elevated levels of Hgb (SMD = 0.13, 95%*CI*: 0.00~0.26; *P* = 0.045) and NEU% (SMD = 0.34, 95%*CI*: 0.19~0.49; *P*<0.001). MON (SMD = -0.28, 95%*CI*: -0.66~0.10; *P* = 0.148), WBC (SMD = -0.08, 95%CI: -0.17~0.01; *P* = 0.08), LYM (SMD = -0.22, 95%*CI*: -0.49~0.05; *P* = 0.11) and NEU (SMD = 0.05, 95%*CI*: -0.08~0.18; *P* = 0.48) were not significantly different between the fatal and non-fatal SFTS patients. Modest heterogeneity was demonstrated from Hgb (*I*^2^ = 38%, *P* = 0.10), LYM% (*I*^2^ = 0, *P* = 0.62), MON% (*I*^2^ = 0, *P* = 0.72), WBC (*I*^2^ = 10%, *P* = 0.31), NEU% (*I*^2^ = 29%, *P =* 0.20) and NEU (*I*^2^ = 0, *P* = 0.60), and obvious heterogeneity was detected from PLT (*I*^2^ = 52%, *P*<0.001), MON (*I*^2^ = 73%, *P* = 0.005), LYM (*I*^2^ = 68%, *P* = 0.002) (S2 Text).

### The relationship between coagulation indicators and fatality of SFTS patients

Coagulation indicators analysis suggested that APTT (SMD = 1.37, 95%*CI*: 1.08~1.65, *P*<0.001), PT (SMD = 0.69, 95%*CI*: 0.45~0.94, *P*<0.001) and TT (SMD = 1.53, 95%*CI*: 0.85~2.20, *P*<0.001) significantly prolonged in fatal SFTS patients when comparing non-fatal SFTS patients, but the level of FIB was diminished (SMD = -0.54, 95%*CI*: -0.90~-0.18, *P*<0.001). Obvious heterogeneities were observed (APTT: *I*^2^ = 82% and *P*<0.001; PT: *I*^2^ = 60% and *P* = 0.004; TT: *I*^2^ = 85% and *P*<0.001; FIB: *I*^2^ = 88% and *P*<0.001) (S2 Text).

### The relationship between liver function indexes and fatality of SFTS patients

Liver function indexes analysis showed that the concentration of GGT (SMD = 0.46, 95%*CI*: 0.26~0.65, *P*<0.001), ALT (SMD = 0.92, 95%*CI*: 0.61~1.24, *P*<0.001), AST (SMD = 1.19, 95%*CI*: 0.87~1.50, *P*<0.001), ALP (SMD = 0.52, 95%*CI*: 0.33~0.72, *P*<0.001) and TB (SMD = 0.52, 95%CI: 0.18~0.86, *P* = 0.003) were significantly higher in patients who died than in those who survived, excluding ALB (SMD = -0.43, 95%*CI*: -0.63~-0.22, *P*<0.001). Obvious heterogeneities were observed from this group of meta-analysis (ALT: *I*^2^ = 85% and *P*<0.001; AST: *I*^2^ = 89% and *P*<0.001; ALB: *I*^2^ = 57% and *P* = 0.007; TB: *I*^2^ = 73% and *P* = 0.005) except ALP (*I*^2^ = 49% and *P* = 0.12) and GGT (*I*^2^ = 39% and *P* = 0.18). (S2 Text).

### The relationship between renal function indexes and fatality of SFTS patients

Renal function indexes analysis showed that the level of BUN (SMD = 0.92, 95%*CI*: 0.79~1.05, *P*<0.001) and sCr (SMD = 0.69, 95%*CI*: 0.49~0.89, *P*<0.001) in fatal SFTS patients was higher than that in non-fatal patients. There was obvious heterogeneity detected from sCr (*I*^2^ = 65% and *P*<0.001). (S2 Text).

### The relationship between myocardial infarction indicators and fatality of SFTS patients

Myocardial infarction indicators analysis indicated that fatal SFTS cases had significantly elevated level of LDH (SMD = 1.13, 95%*CI*: 0.89~1.37, *P*<0.001), CK (SMD = 0.95, 95%*CI*: 0.58~1.32, *P*<0.001) and CK-MB (SMD = 0.70, 95%*CI*: 0.44~0.96, *P*<0.001). An obvious heterogeneity was revealed from the meta-analysis of LDH (*I*^2^ = 79% and *P*<0.001), CK (*I*^2^ = 90%, *P*<0.001) and CK-MB (*I*^2^ = 71%, *P*<0.001). (S2 Text).

### The relationship between other laboratory parameters and fatality of SFTS patients

Further, the concentration of CRP (SMD = 0.50, 95%*CI*: 0.30~0.71, *P*<0.001), D-D (SMD = 0.48, 95%*CI*: 0.16~0.81, *P* = 0.004) and K (SMD = 0.47, 95%*CI*: 0.31~0.63 *P*<0.001) were significantly higher in patients who died than in those who survived, but the level of Na (SMD = -0.06, 95%*CI*: -0.22~0.09, *P* = 0.44) was not significantly associated with the fatal outcome. No heterogeneity was observed from this group of meta-analysis (S2 Text).

### Subgroup analyses and meta-regression

The results of subgroup analyses indicated that number of study sites had effects on heterogeneity of ALT, and sample size had effects on heterogeneity of CK, LDH and PT (S5 Table). Overall, the heterogeneities of prospective and elder age groups were lower. However, subgroup analyses of most of factors had large heterogeneity and unevenly distributed subgroups. ALT, AST, CK, LDH, PLT, PT and viral load contributed to the risk of dying of SFTS patients in each subgroup analyses (S5 Table). Meta-regression in estimates of LDH showed that sample size was significantly associated with the fatality risk of SFTS patients (S3 Text).

### Sensitivity analysis and publication bias

Leave-one-out sensitivity analysis showed that there was no effect of removing any study on the summary estimates but MON and FIB (S4 Text). MON was associated with SFTS fatality after excluding Jia B et al. (SMD = -0.47, 95%*CI*: -0.67~-0.27) [20]. Further, the association between FIB and SFTS fatality became significant after excluding Zhang YZ et al. [46] (SMD = -0.91, 95%*CI*: -1.43~-0.39), respectively (S4 Text). Funnel plot asymmetry was done with the estimates of factors which was reported at least five studies (S5 Text). The Egger’s test suggested statistical evidence of publication bias of ALT (t = 2.47, *P* = 0.022), CK (t = 2.57, *P* = 0.018), AST (t = 2.26, *P* = 0.033), K (t = -3.51, *P* = 0.039) and BUN (t = -4.11, *P* = 0.001) (S5 Text).

## Discussions

For an emerging infectious disease without effective therapy and vaccine, identification of risk factors associated with disease progression is essential to clinical monitoring and treatment, avoiding a fatal outcome to the greatest extent. Though laboratory indicators contributing to fatal clinical outcome of SFTS cases have been investigated in different countries and regions in recent years, unfortunately, no consistent conclusion derived because of sample sizes with enormous differences. Therefore, it is necessary to summarize previous studies for a robust and convincing consequence. We found that elevated level of viral load, PT, ALT, AST, LDH and CK significantly increased the risk of dying of SFTS patients, whereas reduced level of PLT was associated with the fatality of SFTS cases. These findings accord with findings of previous meta-analysis [6–9].

High viral load was found to be associated with mortality in several studies. We found that viral load contributes to the progression of SFTS and fatal outcome development, which has similar effects on different age groups. Preceding the clinical deterioration, significantly enhanced viral load was observed, while the laboratory parameters, especially LDH, AST, CK, PLT, began to deviate sharply from normal ranges [49]. A previous study [37] showed that higher numbers of the virus were capable of inducing higher levels of IFN-inducible protein-10 and macrophage inflammatory protein-1 while repressing the production of activation normal T cell expressed and secreted factor, which further cause the severity and even death [50]. The rapidly rising level of viral load activated the innate and acquired immune system, causing the released of proinflammatory cytokines in quantity, and further aggravating tissues and organs damage [51]. Even worse, the “cytokine storm” formed after the body oversecreted proinflammatory cytokines and anti-inflammatory cytokines would lead to serious immune imbalance and extensive tissues and organs damage, thus accelerating the progress of the disease [52]. Besides, previous study had confirmed the importance of viraemia evaluation and given treatment as early as possible due to the viral-load dependent therapeutic effect of ribavirin for SFTSV infection [1].

The results of routine blood tests failed in consistency intensively according to previous published papers [6,7,9]. This discrepancy can be primarily attributed to the differences of sample sizes and study regions. Our study using large amounts of published literature offered a new and credible perspective on the association between routine blood tests and SFTS patients’ outcome. We found that the reduced levels of PLT, LYM% and MON% were significantly associated with increased risk of mortality, as well as elevated Hgb and NEU%. Especially the PLT, though heterogeneity was detected, all subgroup analyses and sensitivity analysis demonstrated the pooled result was credible and steady. The decrease of PLT is the earliest indicator of laboratory abnormality with almost 100% probability, which can be used as indicator for early diagnosis of the disease [34]. However, considering that the decrease of PLT is also associated with many other diseases, we need other specific methods to identify SFTS patients. In addition, the reason why PLT was significantly correlated with the SFTS patients’ fatality was unclear. A previous study suggested that it might be related to the transient suppression of marrow hemopoietic function caused by viral infection [53]. Furthermore, it has been shown that in a mouse infection model, platelets were adhered to SFTSV, which further promoted the clearance of splenic macrophages [54]. Interestingly, this study did not find the association between WBC and SFTS outcomes even though leukopenia is the typical feature of SFTS patients.

Coagulation dysfunction was common in SFTS patients [27,29,55]. Our study showed that APTT, PT and TT were significantly prolonged in fatal patients compared to the non-fatal, which agreed with previous researches [6–9,53]. Similar to other viral hemorrhagic fevers such as Crimean-Conga hemorrhagic fever, SFTSV infection leads to significant damage of vascular endothelial cells and exposure of subcutaneous collagen fibers, promoting PLT aggregation and cytokines activation, which initiates endogenous coagulation system, and further leads to APTT, TT and PT significantly prolonged [56,57]. At the same time, coagulation disorder can cause secondary damage to endothelial cells, causing disseminated intravascular coagulation (DIC), and aggravating coagulation disorder [15]. Due to the above reasons, haemorrhagic signs were observed commonly in SFTS patients. In an observational study of the largest cohort of patients with SFTS to date, over a third of SFTS cases were signed haemorrhagic signs [1]. What’s more, almost all presentations of bleeding were significantly associated with death, indicating haemorrhagic symptoms should be closely monitored across the disease course. Sensitive analysis and subgroup analysis also showed great robustness, even if heterogeneity existed.

Besides PLT, WBC and APTT, the common laboratory indicators including CK, CK-MB, ALT, AST, LDH and BUN, were identified as abnormal in a high proportion at acute infections. Our study suggested that the elevated levels of indexes above significantly increased mortality risk of SFTS patients. Previous studies [41,58] demonstrated that the measurement indicative of pathological lesions mainly involved the hematological system, liver, kidney, muscle and lymphoid system in different stage, indicating acute inflammation and impairment of liver and renal function is present at an early phase of the illness, corroborating the notion that SFTS is a complicated multisystem disease. Liver and renal function parameters were identified as the critical predictors of fatal outcome because they were confirmed to be the major target organ in SFTSV infected animal model [54]. These factors could have crucial applications if confirmed in other cohorts [58]. Furthermore, in some score models for predicting the mortality of SFTS, LDH and BUN were used widely and were shown to achieve high sensitivity and specificity, suggesting that combined multi-markers representing different damage sources of SFTSV infection played an important role for predicting the disease progression [1,59]. Myocardial damage is another common symptom after SFTSV infection and makes SFTS patients more likely to develop critical cases [60]. One study [60] monitoring and analyzing electrocardiograph (ECG), myocardial enzyme and biochemical indexes of SFTS cases found that more than half of patients had ECG abnormalities with the characteristics of ST-T change, sinus bradycardia and atrial fibrillation. Though the ECG change of SFTS patients was reversible with the improvement of condition, the incidence of abnormal ECG in death patients was still at a high level, suggesting the ECG in critical patients was more difficult to recover. Our study showed that the pooled effect of K was labile, implying further researches were necessary and valuable.

This meta-analysis had some limitations. First, significant heterogeneity brought into question the suitability of performing this meta-analysis. Fortunately, the sensitivity analysis showed that the pooled rates were stable, and subgroup analyses also identified several value factors. Secondly, the published studies only contained hospitalized SFTS patients, which might lead to a likely biased toward severe cases. Third, the sample sizes of most studies were small.

## Conclusion

In conclusion, our review adds to the accumulating evidence on the effect of laboratory indexes on the risk of SFTS patients’ mortality. We found that the abnormal levels of viral load, coagulation function, liver function, significantly increase the risk of SFTS mortality, especially ALT, AST, CK, LDH, PLT and PT, corroborating the notion that SFTS is a complicated multi-system disease. These laboratory parameters should be considered key prognostic factors in future studies. In addition, the findings from the review could be used to facilitate better disease management, not only in China but also in other regions where SFTS is present.

## Supporting information

S1 Table Literature search syntax. CNKI-Chinese National Knowledge Infrastructure.
(DOCX)

S2 Table Key to modified Newcastle-Ottawa Quality Assessment Scale scoring. (DOCX)

S3 Table Overview of all laboratory parameters. PLT-platelet count; LYM-lymphocyte; LYM%-lymphocyte percentage, MON-monocyte; MON%-monocyte percentage; NEU-neutrophil; Hgb-hemoglobin; NEU%-neutrophil percentage; WBC-white blood cell; APTT-activated partial-thromboplastin time; PT-partial-thromboplastin time; TT-thrombin time; FIB-fibrinogen; GGT-gamma glutamyl transferase; ALT-alanine aminotransferase; AST-creatin phosphokinase; ALP-alkaline phosphatase; TB-total bilirubin; ALB-albumin; BUN-blood urea nitrogen; sCr-serum creatinine; LDH-lactate dehydrogenase; CK-creatin phosphokinase; CK-MB-creatinine kinase myocardial b fraction; CRP-C reactive protein; D-D-“D-dimer”; K-potassium; Na-sodium; a-All studies that were included in systematic review. b-Studies that reported a significant association in text, a p-value < 0.05, or 95% confidence intervals not including zero.
(DOCX)

S4 Table The quality evaluation of included studies by NOS scale. (DOCX)

S5 Table Subgroup analysis for factors (at least five studies) with significant heterogeneity. ALB-albumin; ALT-alanine aminotransferase; APTT-activated partial-thromboplastin time; AST-creatin phosphokinase; CK-creatin phosphokinase; CK-MB-creatinine kinase myocardial b fraction; sCr-serum creatinine; LDH-lactate dehydrogenase; PLT-platelet count; PT-partial-thromboplastin time; TT-thrombin time; MON-monocyte; LYM-lymphocyte; TB-total bilirubin; SMD-standardized mean difference; *CI*-confidence interval; z&p(z)-tests of subgroup effect size; p (Comparison)-Cochran’s Q statistics for heterogeneity between subgroups; NA-one subgroup contained only one study; * The study site of all studies was one; # The study site of all studies was one and there was missing value for mean age.
(DOCX)

S1 Text PRISMA 2020 Checklist for the clinical laboratory parameters and fatality of severe fever with thrombocytopenia syndrome patients: A systematic review and meta-analysis. (DOCX)

S2 Text Forest plots of the association between fatal risk of laboratory parameters. PLT-platelet count; LYM%-lymphocyte percentage; MON%-monocyte percentage; Hgb-hemoglobin; NEU%-neutrophil percentage; MON-monocyte; WBC-white blood cell; LYM-lymphocyte; NEU-neutrophil; APTT-activated partial-thromboplastin time; PT-partial-thromboplastin time; TT-thrombin time; FIB-fibrinogen; GGT-gamma glutamyl transferase; ALT-alanine aminotransferase; AST-creatin phosphokinase; ALP-alkaline phosphatase; TB-total bilirubin; ALB-albumin; BUN-blood urea nitrogen; sCr-serum creatinine; LDH-lactate dehydrogenase; CK-creatin phosphokinase; CK-MB-creatinine kinase myocardial b fraction; CRP-C reactive protein; D-D-“D-dimer”; K-potassium; Na-sodium.
(DOCX)

S3 Text Meta-regression analysis for factors (at least ten studies) with significant heterogeneity. ALB-albumin; ALT-alanine aminotransferase; APTT-activated partial-thromboplastin time; AST-creatin phosphokinase; CK-creatin phosphokinase; CK-MB-creatinine kinase myocardial b fraction; sCr-serum creatinine; LDH-lactate dehydrogenase; PLT-platelet count; PT-partial-thromboplastin time; *Ref*-reference unit; *CI*-confidence interval.
(DOCX)

S4 Text Sensitivity analysis for laboratory parameters with significant heterogeneity. ALB-albumin; ALT-alanine aminotransferase; APTT-activated partial-thromboplastin time; AST-creatin phosphokinase; CK-creatin phosphokinase; CK-MB-creatinine kinase myocardial b fraction; sCr-serum creatinine; FIB-fibrinogen; LDH-lactate dehydrogenase; MON-monocyte; PLT-platelet count; PT-partial-thromboplastin time; TB-total bilirubin; TT-thrombin time; LYM-lymphocyte.
(DOCX)

S5 Text Funnel plots for laboratory parameters reported in at least five studies. ALB-albumin; ALT-alanine aminotransferase; APTT-activated partial-thromboplastin time; AST-creatin phosphokinase; BUN-blood urea nitrogen; CK-creatin phosphokinase; CK-MB-creatinine kinase myocardial b fraction; sCr-serum creatinine; CRP-C reactive protein; Hgb-hemoglobin; LDH-lactate dehydrogenase; LYM%-lymphocyte percentage; MON%-monocyte percentage; MON-monocyte; NEU%-neutrophil percentage; NEU-neutrophil; PLT-platelet count; PT-partial-thromboplastin time; TB-total bilirubin; TT-thrombin time; WBC-white blood cell; LYM-lymphocyte.
(DOCX)

## References

[1] Li H, Lu QB, Xing B, Zhang SF, Liu K, Du J, et al. Epidemiological and clinical features of laboratory-diagnosed severe fever with thrombocytopenia syndrome in China, 2011–2017: a prospective observational study. Lancet Infect Dis. 2018; 18(10):1127–1137. doi: 10.1016/S1473-3099(18)30293-7 30054190

[2] Miao D, Liu MJ, Wang YX, Ren X, Lu QB, Zhao GP, et al. Epidemiology and Ecology of Severe Fever with Thrombocytopenia Syndrome in China, 2010–2018. Clin Infect Dis. 2021; 73(11): e3851–e3858. doi: 10.1093/cid/ciaa1561 33068430 PMC8664468

[3] Li J, Li S, Yang L, Cao P, Lu J. Severe fever with thrombocytopenia syndrome virus: a highly lethal bunyavirus. Crit Rev Microbiol. 2021; 47(1):112–125. doi: 10.1080/1040841X.2020.1847037 33245676

[4] Lam TT, Liu W, Bowden TA, Cui N, Zhuang L, Liu K, et al. Evolutionary and molecular analysis of the emergent severe fever with thrombocytopenia syndrome virus. Epidemics. 2013; 5(1): 1–10. doi: 10.1016/j.epidem.2012.09.002 23438426 PMC4330987

[5] Mehand MS, Millett P, Al-Shorbaji F, Roth C, Kieny MP, Murgue B, et al. World Health Organization Methodology to Prioritize Emerging Infectious Diseases in Need of Research and Development. Emerg Infect Dis. 2018; 24(9): e171427. doi: 10.3201/eid2409.171427 30124424 PMC6106429

[6] Li MM, Lei XY, Yu XJ. Meta-analysis of the clinical and laboratory parameters of SFTS patients in China. Virol J. 2016; 13(1): 198. doi: 10.1186/s12985-016-0661-9 27899121 PMC5129669

[7] Wang X, Ren X, Ge Z, Cui S, Wang L, Chen Z, et al. Clinical manifestations of death with severe fever and thrombocytopenia syndrome: A meta-analysis and systematic review. J Med Virol. 2021; 93(6): 3960–3968. doi: 10.1002/jmv.26518 32930400

[8] Dualis H, Zefong AC, Joo LK, Dadar Singh NK, Syed Abdul Rahim SS, Avoi R, et al. Factors and outcomes in Severe Fever with Thrombocytopenia Syndrome (SFTS): A systematic review. Ann Med Surg (Lond). 2021; 67: 102501. doi: 10.1016/j.amsu.2021.102501 34188913 PMC8219640

[9] He Z, Wang B, Li Y, Du Y, Ma H, Li X, et al. Severe fever with thrombocytopenia syndrome: a systematic review and meta-analysis of epidemiology, clinical signs, routine laboratory diagnosis, risk factors, and outcomes. BMC Infect Dis. 2020; 20(1): 575. doi: 10.1186/s12879-020-05303-0 32758175 PMC7409422

[10] Gilmour B, Alene KA, Clarke NE, Clements ACA. The prevalence of tuberculosis, malaria and soil-transmitted helminth infection in minority indigenous people of Southeast Asia and the Western Pacific: protocol for a systematic review and meta-analysis. Syst Rev. 2021; 10(1): 203. doi: 10.1186/s13643-021-01753-y 34246316 PMC8271287

[11] Hozo SP, Djulbegovic B, Hozo I. Estimating the mean and variance from the median, range, and the size of a sample. BMC Med Res Methodol. 2005; 5:13. doi: 10.1186/1471-2288-5-13 15840177 PMC1097734

[12] Wan X, Wang W, Liu J, Tong T. Estimating the sample mean and standard deviation from the sample size, median, range and/or interquartile range. BMC Med Res Methodol. 2014; 14:135. doi: 10.1186/1471-2288-14-135 25524443 PMC4383202

[13] Tufanaru C, Munn Z, Stephenson M, Aromataris E. Fixed or random effects meta-analysis? Common methodological issues in systematic reviews of effectiveness. Int J Evid Based Healthc. 2015; 13(3):196–207. doi: 10.1097/XEB.0000000000000065 26355603

[14] Zhang TS, Liu JB, Zhong WZ. Application of Stata in Exploring Sources of Heterogeneity: Meta-Regression Analysis. The Journal of Evidence-Based Medicine. 2009; 9(1): 48–50. doi: 10.3969/j.issn.1671-5144.2009.01.012

[15] Chen GS, Hu LF, Xu XH, Li JB. The clinical characteristics and prognostic indicators of severe fever with thrombocytopenia syndrome infected by new Bunia virus. China Medical Equipment. 2017; 14(5): 94–97.

[16] Chu SS, Zheng W, Wang JW, Chen YJ, Yu L, Xu CD, et al. Clinical characteristics of 40 patients with severe fever with thrombocytopenia syndrome in Tiantai County. Inter J Epidemiol Infect Dis. 2019; 46(6): 526–529. doi: 10.3760/cma.j.issn.1673-4149.2019.06.016

[17] Gui Y, Xu Y, Yang P. Predictive Value of the Platelet-to-Albumin Ratio (PAR) on the Risk of Death at Admission in Patients Suffering from Severe Fever with Thrombocytopenia Syndrome. J Inflamm Res. 2021; 14: 5647–5652. doi: 10.2147/JIR.S335727 34744448 PMC8565980

[18] Han CX, Sun AJ, Pu CW, Li YT, Sui F, Qin SJ, et al. Epidemiological characteristics of severe fever with thrombocytopenia syndrome caused by novel bunyavirus infection and influencing factors for prognosis. Chin J Nosocomiol. 2019; 29(2):171–174+187.

[19] Hou HH, Mao LL, Liang HY, Liu Y, Liu XS, Deng BC. Clinical characteristics and influencing factors for prognosis of fever with severe thrombocytopenia syndrome in Dalian, Liaoning Province. Chin J Infect Control. 2021; 20(10): 897–902. doi: 10.12138/j.issn.1671-9638.20218284

[20] Jia B, Yan X, Chen Y, Wang G, Liu Y, Xu B, et al. A scoring model for predicting prognosis of patients with severe fever with thrombocytopenia syndrome. PLoS Negl Trop Dis. 2017; 11(9): e0005909. doi: 10.1371/journal.pntd.0005909 28934195 PMC5626493

[21] Kato H, Yamagishi T, Shimada T, Matsui T, Shimojima M, Saijo M, et al. SFTS epidemiological research group-Japan. Epidemiological and Clinical Features of Severe Fever with Thrombocytopenia Syndrome in Japan, 2013–2014. PLoS One. 2016; 11(10): e0165207. doi: 10.1371/journal.pone.0165207 27776187 PMC5077122

[22] Kwon JS, Jin S, Kim JY, Ra SH, Kim T, Park SY, et al. Viral and Immunologic Factors Associated with Fatal Outcome of Patients with Severe Fever with Thrombocytopenia in Korea. Virus. 2021; 13(12): 2351. doi: 10.3390/v13122351 34960620 PMC8703577

[23] Li J, Han Y, Xing Y, Li S, Kong L, Zhang Y, et al. Concurrent measurement of dynamic changes in viral load, serum enzymes, T cell subsets, and cytokines in patients with severe fever with thrombocytopenia syndrome. PLoS One. 2014; 9(3): e91679. doi: 10.1371/journal.pone.0091679 24658451 PMC3962368

[24] Li MM, Zhang WJ, Weng XF, Li MY, Liu J, Xiong Y, et al. CD4 T cell loss and Th2 and Th17 bias are associate with the severity of severe fever with thrombocytopenia syndrome (SFTS). Clin Immunol. 2018; 195: 8–17. doi: 10.1016/j.clim.2018.07.009 30036637 PMC7185468

[25] Liu JY, Feng J, Li AL, Wang SY, Zheng R, Chen HZ. Analysis of clinical characteristics and death risk factors in patients infected with severe fever with thrombocytopenia syndrome bunyavirus. Chin J Postgrad Med, 2018; 41(5):429–433. doi: 10.3760/cma.j.issn.1673-4904.2018.05.012

[26] Liu W, Lu QB, Cui N, Li H, Wang LY, Liu K, et al. Case-fatality ratio and effectiveness of ribavirin therapy among hospitalized patients in china who had severe fever with thrombocytopenia syndrome. Clin Infect Dis. 2013; 57(9):1292–9. doi: 10.1093/cid/cit530 23965284

[27] Nie Q, Wang D, Ning Z, Li T, Tian X, Bian P, et al. Analysis of Severe Fever With Thrombocytopenia Syndrome in Critical Ill Patients in Central China. Shock. 2020; 54(4):451–457. doi: 10.1097/SHK.0000000000001527 32097243

[28] Peng C, Wang H, Zhang W, Zheng X, Tong Q, Jie S, et al. Decreased monocyte subsets and TLR4-mediated functions in patients with acute severe fever with thrombocytopenia syndrome (SFTS). Int J Infect Dis. 2016; 43:37–42. doi: 10.1016/j.ijid.2015.12.009 26701820

[29] Sheng QY, Sheng JF, Zhang X, Ye WW, Huang HJ. Clinical characteristics and prognostic factors of 25 patients with new bunyavirus infection. Chin J Crit Care Med (Electronic Edition) 2019; 12: 152–157.

[30] Shin J, Kwon D, Youn SK, Park JH. Characteristics and Factors Associated with Death among Patients Hospitalized for Severe Fever with Thrombocytopenia Syndrome, South Korea, 2013. Emerg Infect Dis. 2015; 21:1704–1710. doi: 10.3201/eid2110.141928 26402575 PMC4593431

[31] Suemori K, Saijo M, Yamanaka A, Himeji D, Kawamura M, Haku T, et al. A multicenter non-randomized, uncontrolled single arm trial for evaluation of the efficacy and the safety of the treatment with favipiravir for patients with severe fever with thrombocytopenia syndrome. PLoS Negl Trop Dis. 2021; 15(2): e0009103. doi: 10.1371/journal.pntd.0009103 33617533 PMC7899362

[32] Sun L, Hu Y, Niyonsaba A, Tong Q, Lu L, Li H, et al. Detection and evaluation of immunofunction of patients with severe fever with thrombocytopenia syndrome. Clin Exp Med. 2014; 14(4):389–95. doi: 10.1007/s10238-013-0259-0 24068614 PMC7101760

[33] Sun Y, Jin C, Zhan F. Wang X, Lian M, Zhang Q, et al. Host cytokine storm is associated with disease severity of severe fever with thrombocytopenia syndrome. J Infect Dis. 2012; 206(7): 1085–94. doi: 10.1093/infdis/jis452 22904342

[34] Tan QL, Ren Y, Yang ZN, Lin JF, Ye L, Li SB. Epidemiology, clinical characteristics and gene sequence of fatal cases of severe fever with thrombocytopenia syndrome in Zhoushan island, China. Chinese Journal of Zoonoses 2016; 32: 70–75. doi: 10.3969/j.issn.1002-2694.2016.01.015

[35] Wang F, Wu Y, Jiao J, Wang J, Ge Z. Risk Factors and Clinical Characteristics of Severe Fever with Thrombocytopenia Syndrome. Int J Gen Med. 2020; 13: 1661–1667. doi: 10.2147/IJGM.S292735 33408503 PMC7779285

[36] Wang L, Wan G, Shen Y, Zhao Z, Lin L, Zhang W, et al. A nomogram to predict mortality in patients with severe fever with thrombocytopenia syndrome at the early stage-A multicenter study in China. PLoS Negl Trop Dis. 2019; 13(11): e0007829. doi: 10.1371/journal.pntd.0007829 31765414 PMC6934327

[37] Wang L, Zou Z, Ding K, Hou C. Predictive risk score model for severe fever with thrombocytopenia syndrome mortality based on qSOFA and SIRS scoring system. BMC Infect Dis. 2020; 20(1): 595. doi: 10.1186/s12879-020-05299-7 32787952 PMC7425036

[38] Xiao LY, Shi DY, Liu YF, Zheng YS. Clinical characteristics and treatment efficacy of sever infection caused by new bunyaviridae. Electronic Journal of Emerging Infectious Diseases. 2020; 5(1): 16–19.

[39] Xiong S, Zhang W, Li M, Xiong Y, Li M, Wang H, et al. A simple and practical score model for predicting the mortality of severe fever with thrombocytopenia syndrome patients. Medicine (Baltimore). 2016; 95(52): e5708. doi: 10.1097/MD.0000000000005708 28033271 PMC5207567

[40] Yang B, Wang X, Li Y, Wu A, Liu Q, Lu Y, et al. A Newly Established Severity Scoring System in Predicting the Prognosis of Patients with Severe Fever with Thrombocytopenia Syndrome. Tohoku J Exp Med. 2017; 242(1): 19–25. doi: 10.1620/tjem.242.19 28496029

[41] Yang M, Ye J, Li H, Hua TF, Zheng Y, Li J. Investigation of clinical characteristics and prognosis of severe fever with thrombocytopenia syndrome: 69 cases analysis. Chin J Dis Control Prev. 2018; 22(4): 402–405.

[42] Yin M, Zhao ZH, Yang Y. Risk factors for death in 95 patients with fever and thrombocytopenia syndrome. Journal of Anhui Health Vocational & Technical College. 2020; 19(4): 21–23+25.

[43] Yoo JR, Kim TJ. Heo ST, Hwang KA, Oh H, Ha T, et al. IL-6 and IL-10 Levels, Rather Than Viral Load and Neutralizing Antibody Titers, Determine the Fate of Patients With Severe Fsver With Thrombocytopenia Syndrome Virus Infection in South Korea. Front Immunol. 2021; 12:711847. doi: 10.3389/fimmu.2021.711847 34484214 PMC8416084

[44] You EQ, Wang L, Zhang L, Wu J, Zhao K, Huang F. Epidemiological characteristics of severe fever with thrombocytopenia syndrome in surveillance study from 2011 to 2018. Eur J Clin Microboil Infect Dis. 2021; 40(5): 929–939. doi: 10.1007/s10096-020-04098-x 33188497

[45] Zeng QQ, Wang QJ, Zhang JJ, Yang ZJ, Li YC, Zhu HM, et al. Risk factors for mortality in patients with severe fever with thrombocytopenia syndrome. Chin J Infect Dis. 2017; 35(6):336–340. doi: 10.3760/cma.j.issn.1000-6680.2017.06.004

[46] Zhang YZ, He YW, Dai YA, Xiong Y, Zheng H, Zhou DJ, et al. Hemorrhagic fever caused by a novel Bunyavirus in China: pathogenesis and correlates of fatal outcome. Clin Infect Dis. 2012; 54(4):527–33. doi: 10.1093/cid/cir804 22144540

[47] Zhao H, Yang GL, Han Y, Han XY, Lv Y, Ding XM, et al. Serum cytokines and chemokines in patients with severe fever with thrombocytopenia syndrome. Chinese J Exp Clin Virol. 2020; 34(5): 537–542.

[48] Zhou SJ, Xia GM, He TF, Xu MY, Ye J, Li X, et al. Clinical characteristics and prognostic factors of patients infected with novel Bunyavirus. Acta Universitatis Medicinalis Anhui. 2021; 56(6): 942–947.

[49] Yang ZD, Hu JG, Lu QB, Guo CT, Cui N, Peng W, et al. The prospective evaluation of viral loads in patients with severe fever with thrombocytopenia syndrome. J Clin Virol. 2016; 78: 123–8. doi: 10.1016/j.jcv.2016.03.017 27062673

[50] He Z, Wang B, Li Y, Hu K, Yi Z, Ma H, et al. Changes in peripheral blood cytokines in patients with severe fever with thrombocytopenia syndrome. J Med Virol. 2021; 93(8): 4704–4713. doi: 10.1002/jmv.26877 33590892 PMC8360139

[51] Li YP, Liu CR, Jia XL, Cui S, Zhang X, Dang SS. Etiology and pathogenesis of coronavirus. Clinical Research and Practice. 2020; 5(1): 12–15.

[52] Han YP, Dong L, Kong LH, Zhang LL, Liu N, Chen N, et al., Viral load and cytokines in the pathogenesis of severe fever with thrombocytopenia syndrome. Chin J Infect Dis. 2014; 32(9):538–544.

[53] Yu Z, Ouyang TZ, Zhou P. Risk factors predicting fatality of severe fever complicated with thrombocytopenia syndrome: a meta-analysis[J]. IMHGN. 2020; 26(14):2054–2063.

[54] Jin C, Liang M, Ning J, Gu W, Jiang H, Wu W, et al. Pathogenesis of emerging severe fever with thrombocytopenia syndrome virus in C57/BL6 mouse model. Proc Natl Acad Sci USA. 2012; 109(25): 10053–10058. doi: 10.1073/pnas.1120246109 22665769 PMC3382536

[55] Ma YQ, Liang MM, Yin HF. Clinical characteristics and prognostic factors of new bunia viral infection. The Journal of Practical Medicine. 2020; 36(23): 3231–3235, 3236. doi: 10.3969/j.issn.1006-5725.2020.23.013

[56] Zhao HY, Sun J, Yan XM, Xiong YL, Huang R, Zhang YY, et al. Clinical characteristics and risk factors for mortality of patients with severe fever with thrombocytopenia syndrome. Chin J Infect Dis. 2016; 34(1):15–18. doi: 10.3760/cma.j.issn.1000-6680.2016.01.004

[57] Ergonul O, Tuncbilek S, Baykam N, Celikbas A, Dokuzoguz B. Evaluation of serum levels of interleukin (IL)-6, IL-10, and tumor necrosis factor-alpha in patients with Crimean-Congo hemorrhagic fever. J Infect Dis. 2006; 193:941–4. doi: 10.1086/500836 16518755

[58] Yu XJ. Risk factors for death in severe fever with thrombocytopenia syndrome. Lancet Infect Dis. 2018; 18(10): 1056–1057. doi: 10.1016/S1473-3099(18)30312-8 30054189

[59] Wang L, Liu XZ, Lin Q. Prognostic value of laboratory parameters in patients with severe fever thrombocytopenia syndrome. Inter J Epidemiol Infect Dis. 2017; 44(6):370–373. doi: 10.3760/cma.j.issn.1673-4149.2017.06.003

[60] Shao F, Zhang Y, Hu XM, Zhang LH, Bian PF, Li Q. Characteristics and significances of ECG in patients of severe fever with thrombocytopenia syndrome. Chin J Exp Clin Dis (Electronic Edition). 2015; 9(6):34–37.

