# Supplementary material for: Clinical laboratory parameters and fatality of Severe fever with thrombocytopenia syndrome patients: A systematic review and meta-analysis
Source: PLoS Negl Trop Dis. 2022 Jun 17;16(6):e0010489. doi: 10.1371/journal.pntd.0010489 (PMC9246219; doi:10.1371/journal.pntd.0010489)
Supplement: S1 Table — CNKI-Chinese National Knowledge Infrastructure. (DOCX) [file pntd.0010489.s001.docx]

**S1 Table. Literature search syntax**

| Database | Result | Date of search | Search string |
| --- | --- | --- | --- |
| PubMed | 359 | 24/2/2022 | **(outcome [Title/Abstract] OR fatal [Title/Abstract] OR non-fatal [Title/Abstract] OR survival [Title/Abstract] OR death [Title/Abstract] OR survivor [Title/Abstract] OR survived [Title/Abstract] OR deceased [Title/Abstract] OR epidemiological [Title/Abstract]) AND (SFTS [Title/Abstract] OR “severe fever with thrombocytopenia syndrome” [Title/Abstract] OR “Dabie bandavirus” [Title/Abstract] OR bunyavirus [Title/Abstract])** |
| Web of Science | 456 | 24/2/2022 | TS = (outcome OR epidemiological OR fatal OR non-fatal OR survival OR death OR survived OR deceased OR prognosis) AND TS = (SFTS OR "severe fever with thrombocytopenia syndrome" OR "Dabie bandavirus" OR bunyavirus) |
| CNKI | 725 | 24/2/2022 | TKA= (outcome + epidemiological + fatal + non-fatal + survival + death + survivor + survived + deceased) AND TKA= (SFTS + “severe fever with thrombocytopenia syndrome” + “**Dabie bandavirus”** + **bunyavirus**) AND TKA= (patient + case) |
| Wanfang | 294 | 24/2/2022 | Title/Abstract:(outcome OR epidemiological OR fatal OR non-fatal OR survival OR death OR survivor OR survived OR deceased) and Title/Abstract:( “severe fever with thrombocytopenia syndrome” OR bunyavirus OR “Dabie bandavurus”) |

.
