# Supplementary material for: Clinical laboratory parameters and fatality of Severe fever with thrombocytopenia syndrome patients: A systematic review and meta-analysis
Source: PLoS Negl Trop Dis. 2022 Jun 17;16(6):e0010489. doi: 10.1371/journal.pntd.0010489 (PMC9246219; doi:10.1371/journal.pntd.0010489)
Supplement: S3 Table — PLT-platelet count; LYM-lymphocyte; LYM%-lymphocyte percentage, MON-monocyte; MON%-monocyte percentage; NEU-neutrophil; Hgb-hemoglobin; NEU%-neutrophil percentage; WBC-white blood cell; APTT-activated partial-thromboplastin time; PT-partial-thromboplastin time; TT-thrombin time; FIB-fibrinogen; GGT-gamma glutamyl transferase; ALT-alanine aminotransferase; AST-creatin phosphokinase; ALP-alkaline phosphatase; TB-total bilirubin; ALB-albumin; BUN-blood urea nitrogen; sCr-serum creatinine; LDH-lactate dehydrogenase; CK-creatin phosphokinase; CK-MB-creatinine kinase myocardial b fraction; CRP-C reactive protein; D-D-“D-dimer”; K-potassium; Na-sodium; a-All studies that were included in systematic review. b-Studies that reported a significant association in text, a p-value < 0.05, or 95% confidence intervals not including zero. (DOCX) [file pntd.0010489.s003.docx]

**S3 Table. Overview of all laboratory parameters**

| **Laboratory parameters** | **Number of studies^a^** | **N significant^b^** | **Reference of N significant** |
| --- | --- | --- | --- |
| **Viral load** | 10 | 10 | [1,2,3,4,5,6,7,8,9,10] |
| **Routine tests** |  |  |  |
| PLT | 30 | 15 | [1,2,4,6,8,11,12-20] |
| LYM | 9 | 3 | [12,18,20] |
| LYM% | 7 | 2 | [8,18] |
| MON | 5 | 2 | [12,18] |
| MON% | 5 | 4 | [3,8,18,21] |
| NEU | 12 | 0 | NA |
| Hgb | 10 | 1 | [15] |
| NEU% | 8 | 3 | [8,18,21] |
| WBC | 28 | 2 | [19,22] |
| **Coagulation indicators** |  |  |  |
| APTT | 19 | 17 | [1,2,4,7,8,10-13,17,18,20,21,23-26] |
| PT | 12 | 10 | [1,8,11-13,17,18,20,23,25] |
| TT | 5 | 4 | [2,8,10,13] |
| FIB | 3 | 2 | [10,26] |
| **Liver function indexes** |  |  |  |
| GGT | 4 | 3 | [2,8,15] |
| ALT | 23 | 10 | [1,4,8,9,11,12,15,18,20,23] |
| AST | 27 | 19 | [1-4,8-12,14,15,17-20,25,27,29,30] |
| ALP | 4 | 3 | [2,8,15] |
| TB | 5 | 1 | [18] |
| ALB | 12 | 6 | [1,2,12,14,20,27] |

| **Laboratory parameters** | **Number of studies^a^** | **N significant^b^** | **Reference of N significant** |
| --- | --- | --- | --- |
| **Renal function indexes** |  |  |  |
| BUN | 15 | 9 | [1,2,5,12,17,18,20,25,28] |
| sCr | 19 | 12 | [1,2,3,8,14,17-21,28,29] |
| **Myocardial infarction indicators** |  |  |  |
| LDH | 22 | 15 | [1,2,4,6,8,13,15,17-20,24,25,26,30] |
| CK | 22 | 14 | [1-4,6,8,11,15,18,20,23,24,26,30] |
| CK-MB | 11 | 7 | [6,13,18,19,20,21,23] |
| **Other laboratory parameters** |  |  |  |
| CRP | 7 | 2 | [1,20] |
| D-D | 4 | 0 | NA |
| K | 5 | 3 | [18,20,24] |
| Na | 6 | 0 | NA |

**References**

1. Hou HH, Mao LL, Liang HY, Liu Y, Liu XS, Deng BC. Clinical characteristics and influencing factors for prog-nosis of fever with severe thrombocytopenia syndrome in Dalian, Liaoning Province. Chin J Infect Control. 2021; 20(10): 897-902. DOI: 10.12138/j.issn.1671-9638.20218284.

2. Jia B, Yan X, Chen Y, Wang G, Liu Y, Xu B, et al. A scoring model for predicting prognosis of patients with severe fever with thrombocytopenia syndrome. PLoS Negl Trop Dis. 2017; 11(9): e0005909. DOI: 10.1371/journal.pntd.0005909.

3. Li MM, Zhang WJ, Weng XF, Li MY, Liu J, Xiong Y, et al. CD4 T cell loss and Th2 and Th17 bias are associate with the severity of severe fever with thrombocytopenia syndrome (SFTS). Clin Immunol. 2018; 195: 8-17. DOI: 10.1016/j.clim.2018.07.009.

4. Liu JY, Feng J, Li AL, Wang SY, Zheng R, Chen HZ. Analysis of clinical characteristics and death risk factors in pa-tients infected with severe fever with thrombocytopenia syndrome bunyavirus. Chin J Post-grad Med, 2018; 41(5):429-433. DOI: 10.3760/cma.j.issn.1673-4904.2018.05.012.

5. Suemori K, Saijo M, Yamanaka A, Himeji D, Kawamura M, Haku T, et al. A multicenter non-randomized, uncontrolled single arm trial for evaluation of the efficacy and the safety of the treatment with favipiravir for pa-tients with severe fever with thrombocytopenia syndrome. PLoS Negl Trop Dis. 2021; 15(2): e0009103. DOI: 10.1371/journal.pntd.0009103.

6. Sun Y, Jin C, Zhan F. Wang X, Lian M, Zhang Q, et al. Host cytokine storm is associated with disease severity of severe fever with thrombocytopenia syndrome. J Infect Dis. 2012; 206(7): 1085-94. DOI: 10.1093/infdis/jis452.

7. Xiao LY, Shi DY, Liu YF, Zheng YS. Clinical characteristics and treatment efficacy of severe infection caused by new bunyaviridae. Electronic Journal of Emerging Infectious Diseases. 2020; 5(1): 16-19. DOI: 0.19871/j.cnki.xfcrbzz.2020.01.003.

8. Xiong S, Zhang W, Li M, Xiong Y, Li M, Wang H, et al. A simple and practical score model for predicting the mortal-ity of severe fever with thrombocytopenia syndrome patients. Medicine (Baltimore). 2016; 95(52): e5708. DOI: 10.1097/MD.0000000000005708.

9. Yoo JR, Kim TJ. Heo ST, Hwang KA, Oh H, Ha T, et al. IL-6 and IL-10 Levels, Rather Than Viral Load and Neutral-izing Antibody Titers, Determine the Fate of Patients With Severe Fsver With Thrombocy-topenia Syndrome Virus Infection in South Korea. Front Immunol. 2021; 12:711847. DOI: 10.3389/fimmu.2021.711847.

10. Zhang YZ, He YW, Dai YA, Xiong Y, Zheng H, Zhou DJ, et al. Hemorrhagic fever caused by a novel Bunyavirus in China: pathogenesis and correlates of fatal outcome. Clin Infect Dis. 2012; 54(4):527-33. DOI:10.1093/cid/cir804.

11. Chen GS, Hu LF, Xu XH, Li JB. The clinical characteristics and prognostic indicators of se-vere fever with thrombocytopenia syndrome infected by new Bunia virus. China Medical Equipment. 2017; 14(5): 94-97. DOI: 10.3969/J.ISSN.1672-8270.2017.05.025.

12. Gui Y, Xu Y, Yang P. Predictive Value of the Platelet-to-Albumin Ratio (PAR) on the Risk of Death at Admission in Patients Suffering from Severe Fever with Thrombocytopenia Syn-drome. J Inflamm Res. 2021; 14: 5647-5652. DOI: 10.2147/JIR.S335727.

13. Han CX, Sun AJ, Pu CW, Li YT, Sui F, Qin SJ, et al. Epidemiological characteristics of severe fever with thrombocytopenia syndrome caused by novel bunyavirus infection and influencing factors for prognosis. Chin J Nosocomiol. 2019; 29(2):171-174+187. DOI: 10.11816/cn.ni.2019-180685.

14. Kato H, Yamagishi T, Shimada T, Matsui T, Shimojima M, Saijo M, et al. SFTS epidemiological research group-Japan. Epide-miological and Clinical Features of Severe Fever with Thrombocytopenia Syndrome in Ja-pan, 2013-2014. PLoS One. 2016; 11(10): e0165207. DOI: 10.1371/journal.pone.0165207.

15. Liu W, Lu QB, Cui N, Li H, Wang LY, Liu K, et al. Case-fatality ratio and effectiveness of ribavirin therapy among hospitalized patients in china who had severe fever with thrombocytopenia syndrome. Clin Infect Dis. 2013; 57(9):1292-9. DOI: 10.1093/cid/cit530.

16. Tan QL, Ren Y, Yang ZN, Lin JF, Ye L, Li SB. Epidemiology, clinical characteristics and gene sequence of fatal cases of severe fever with thrombocytopenia syndrome in Zhoushan island, China. Chinese Journal of Zoonoses 2016; 32: 70-75. DOI: 10.3969/j.issn.1002-2694.2016.01.015.

17. Wang F, Wu Y, Jiao J, Wang J, Ge Z. Risk Factors and Clinical Characteristics of Severe Fever with Thrombocytopenia Syndrome. Int J Gen Med. 2020; 13: 1661-1667. DOI: 10.2147/IJGM.S292735.

18. Wang L, Wan G, Shen Y, Zhao Z, Lin L, Zhang W, et al. A nomogram to predict mortality in patients with severe fever with thrombocytopenia syndrome at the early stage-A multicenter study in China. PLoS Negl Trop Dis. 2019; 13(11): e0007829. DOI: 10.1371/journal.pntd.0007829.

19. Yang M, Ye J, Li H, Hua TF, Zheng Y, Li J. Investigation of clinical characteristics and prognosis of severe fever with thrombocytopenia syndrome: 69 cases analysis. Chin J Dis Control Prev. 2018; 22(4): 402-405. DOI: 10.16462/j.cnki.zhjbkz.2018.04.018.

20. Zhou SJ, Xia GM, He TF, Xu MY, Ye J, Li X, et al. Clinical characteristics and prognostic factors of patients infected with novel Bunyavirus. Acta Universitatis Medicinalis Anhui. 2021; 56(6): 942-947. DOI: 10.19405/j.cnki.issn1000-1492.2021.06.020.

21. Nie Q, Wang D, Ning Z, Li T, Tian X, Bian P, et al. Analysis of Severe Fever With Thrombocytopenia Syndrome in Critical Ill Patients in Central China. Shock. 2020; 54(4):451-457. DOI: 10.1097/SHK.0000000000001527.

22. Peng C, Wang H, Zhang W, Zheng X, Tong Q, Jie S, et al. Decreased monocyte subsets and TLR4-mediated functions in patients with acute severe fever with thrombocytopenia syndrome (SFTS). Int J Infect Dis. 2016; 43:37-42. DOI: 10.1016/j.ijid.2015.12.009.

23. Sheng QY, Sheng JF, Zhang X, Ye WW, Huang HJ. Clinical characteristics and prognostic factors of 25 patients with new bunyavirus infection. Chin J Crit Care Med (Electronic Edition) 2019; 12: 152-157. DOI: 10.3877/cma.j.issn.1674-6880.2019.03.002.

24. Yang B, Wang X, Li Y, Wu A, Liu Q, Lu Y, et al. A Newly Established Severity Scoring System in Predicting the Prognosis of Patients with Severe Fever with Thrombocytopenia Syndrome. Tohoku J Exp Med. 2017; 242(1): 19-25. DOI: 10.1620/tjem.242.19.

25. Yin M, Zhao ZH, Yang Y. Risk factors for death in 95 patients with fever and thrombo-cytopenia syndrome. Journal of Anhui Health Vocational & Technical College. 2020; 19(4): 21-23+25. DOI: 10.3969/j.issn.1671-8054.2020.04.009.

26. Zeng QQ, Wang QJ, Zhang JJ, Yang ZJ, Li YC, Zhu HM, et al. Risk factors for mortality in patients with severe fever with thrombocytopenia syndrome. Chin J Infect Dis. 2017; 35(6):336-340. DOI: 10.3760/cma.j.issn.1000-6680.2017.06.004.

27. Sun L, Hu Y, Niyonsaba A, Tong Q, Lu L, Li H, et al. Detection and evaluation of immunofunction of patients with severe fever with thrombocytopenia syndrome. Clin Exp Med. 2014; 14(4):389-95. DOI: 10.1007/s10238-013-0259-0.

28. Kwon JS, Jin S, Kim JY, Ra SH, Kim T, Park SY, et al. Viral and Immunologic Factors Associated with Fatal Outcome of Patients with Severe Fever with Thrombocytopenia in Korea. Virus. 2021; 13(12): 2351. DOI: 10.3390/v13122351.

29. Wang L, Zou Z, Ding K, Hou C. Predictive risk score model for severe fever with thrombocytopenia syndrome mortality based on qSOFA and SIRS scoring system. BMC Infect Dis. 2020; 20(1): 595. DOI: 10.1186/s12879-020-05299-7.

30. Li J, Han Y, Xing Y, Li S, Kong L, Zhang Y, et al. Concurrent measurement of dynamic changes in viral load, serum enzymes, T cell subsets, and cytokines in patients with severe fever with thrombocytopenia syndrome. PLoS One. 2014; 9(3): e91679. DOI: 10.1371/journal.pone.0091679.
