# Supplementary material for: Clinical laboratory parameters and fatality of Severe fever with thrombocytopenia syndrome patients: A systematic review and meta-analysis
Source: PLoS Negl Trop Dis. 2022 Jun 17;16(6):e0010489. doi: 10.1371/journal.pntd.0010489 (PMC9246219; doi:10.1371/journal.pntd.0010489)
Supplement: S5 Table — ALB-albumin; ALT-alanine aminotransferase; APTT-activated partial-thromboplastin time; AST-creatin phosphokinase; CK-creatin phosphokinase; CK-MB-creatinine kinase myocardial b fraction; sCr-serum creatinine; LDH-lactate dehydrogenase; PLT-platelet count; PT-partial-thromboplastin time; TT-thrombin time; MON-monocyte; LYM-lymphocyte; TB-total bilirubin; SMD-standardized mean difference; CI-confidence interval; z&p(z)-tests of subgroup effect size; p (Comparison)-Cochran’s Q statistics for heterogeneity between subgroups; NA-one subgroup contained only one study; * The study site of all studies was one; # The study site of all studies was one and there was missing value for mean age. (DOCX) [file pntd.0010489.s005.docx]

**S5 Table. Subgroup analysis for factors (at least five studies) with significant heterogeneity**

| Laboratory parameters | Subgroup | | No. of studies | SMD (95%*CI*) | *z* | p(*z*) | *I^2^* | p (Comparison) |
| --- | --- | --- | --- | --- | --- | --- | --- | --- |
| Viral load | Mean age (years) | <60 | 5 | 1.37 (0.95, 1.79) | 6.404 | <0.001 | 58.2 | 0.705 |
|  |  | 60-65 | 3 | 1.93 (0.22, 3.63) | 2.217 | 0.002 | 92.9 |  |
|  |  | >65 | 2 | 1.18 (0.50, 1.85) | 3.413 | 0.001 | 34.1 |  |
|  | Sample size | <50 | 4 | 1.31 (0.68, 1.94) | 4.065 | <0.001 | 49.8 | 0.798 |
|  |  | 50-100 | 3 | 1.93 (0.22, 3.63) | 2.217 | 0.027 | 92.9 |  |
|  |  | >100 | 3 | 1.35 (0.90, 1.80) | 5.870 | <0.001 | 70.9 |  |
|  | Study site | One | 6 | 1.53 (0.81, 2.25) | 4.173 | <0.001 | 87.2 | 0.685 |
|  |  | Two or more | 4 | 1.36 (0.94, 1.78) | 6.410 | <0.001 | 30.6 |  |
|  | Study type | Retrospective | 7 | 1.44 (0.85, 2.03) | 4.754 | <0.001 | 86.0 | 0.600 |
|  |  | Prospective | 3 | 1.64 (1.17, 2.11) | 6.815 | <0.001 | 0 |  |
| ALB | Mean age (years) | <60 | 3 | -0.51 (-0.87, -0.15) | -2.797 | 0.005 | 30.1 | 0.600 |
|  |  | 60-65 | 4 | -0.31 (-0.53, -0.09) | -2.816 | 0.005 | 45.4 |  |
|  |  | >65 | 5 | -0.46 (-1.01, 0.09) | -1.652 | 0.099 | 70.1 |  |
|  | Sample size | <50 | 4 | -0.62 (-1.07, -0.16) | -2.636 | 0.008 | 71.7 | 0.351 |
|  |  | 50-100 | 1 | 0.06 (-0.75, 0.88) | 0.151 | 0.880 | NA |  |
|  |  | >100 | 7 | -0.39 (-0.65, -0.12) | -4.038 | <0.001 | 58.3 |  |
|  | Study site | One | 8 | -0.37 (-0.56, -0.17) | -3.660 | <0.001 | 32.5 | 0.484 |
|  |  | Two or more | 4 | -0.59 (-1.17, 0.00) | -1.973 | 0.048 | 80.8 |  |
|  | Study type | Retrospective | 8 | -0.52 (-0.79, -0.25) | -3.835 | <0.001 | 63.8 | 0.055 |
|  |  | Prospective | 4 | -0.19 (-0.40, 0.02) | -1.809 | 0.070 | 0 |  |
| ALT | Mean age (years) | <60 | 7 | 1.27 (0.33, 2.21) | 2.651 | 0.008 | 91.3 | 0.644 |
|  |  | 60-65 | 12 | 0.90 (0.54, 1.26) | 4.876 | <0.001 | 85.4 |  |
|  |  | >65 | 4 | 0.76 (0.22, 1.29) | 2.771 | 0.006 | 57.1 |  |

| Laboratory parameters | Subgroup | | No. of studies | SMD (95%*CI*) | *z* | p(*z*) | *I^2^* | p (Comparison) |
| --- | --- | --- | --- | --- | --- | --- | --- | --- |
| ALT | Sample size | <50 | 10 | 1.18 (0.32, 2.05) | 2.680 | 0.007 | 88.3 | 0.068 |
|  |  | 50-100 | 5 | 1.62 (0.64, 2.60) | 3.233 | 0.001 | 89.8 |  |
|  |  | >100 | 8 | 0.60 (0.39, 0.81) | 5.590 | <0.001 | 64.6 |  |
|  | Study site | One | 15 | 1.16 (0.70, 1.62) | 4.906 | <0.001 | 89.4 | 0.036 |
|  |  | Two or more | 8 | 0.57 (0.27, 0.87) | 3.742 | <0.001 | 60.2 |  |
|  | Study type | Retrospective | 16 | 1.01 (0.58, 1.44) | 4.644 | <0.001 | 89.8 | 0.505 |
|  |  | Prospective | 7 | 0.85 (0.66, 1.04) | 8.693 | <0.001 | 0 |  |
| APTT | Mean age (years) | <60 | 6 | 1.56 (0.83, 2.30) | 4.149 | <0.001 | 88.9 | 0.154 |
|  |  | 60-65 | 9 | 0.85 (0.37, 1.32) | 3.456 | <0.001 | 89.4 |  |
|  |  | >65 | 4 | 1.72 (0.60, 2.84) | 3.000 | 0.003 | 92.0 |  |
|  | Sample size | <50 | 5 | 2.15 (1.13, 3.16) | 4.150 | <0.001 | 79.9 | 0.127 |
|  |  | 50-100 | 5 | 0.88 (-0.03, 1.79) | 1.903 | 0.057 | 90.4 |  |
|  |  | >100 | 9 | 1.07 (0.61, 1.52) | 6.515 | <0.001 | 91.7 |  |
|  | Study site | One | 13 | 1.46 (0.97, 1.96) | 5.794 | <0.001 | 90.3 | 0.083 |
|  |  | Two or more | 6 | 0.78 (0.19, 1.37) | 2.583 | 0.010 | 89.0 |  |
|  | Study type | Retrospective | 15 | 1.42 (1.04, 1.81) | 7.276 | <0.001 | 85.9 | 0.084 |
|  |  | Prospective | 4 | 0.46 (-0.55, 1.48) | 0.895 | 0.371 | 95.0 |  |
| AST | Mean age (years) | <60 | 8 | 1.62 (063, 2.61) | 3.218 | 0.001 | 92.5 | 0.196 |
|  |  | 60-65 | 14 | 1.25 (0.84, 1.66) | 5.992 | <0.001 | 90.9 |  |
|  |  | >65 | 5 | 0.90 (0.62, 1.18) | 7.762 | <0.001 | 0 |  |
|  | Sample size | <50 | 11 | 1.33 (0.45, 2.21) | 2.974 | 0.003 | 89.5 | 0.117 |
|  |  | 50-100 | 7 | 1.92 (0.99, 2.85) | 4.057 | <0.001 | 91.9 |  |
|  |  | >100 | 9 | 0.93 (0.62, 1.25) | 5.798 | <0.001 | 86.7 |  |
|  | Study site | One | 18 | 1.40 (0.98, 1.83) | 6.492 | <0.001 | 90.4 | 0.127 |
|  |  | Two or more | 9 | 0.89 (0.39, 1.39) | 3.469 | 0.001 | 86.7 |  |

| Laboratory parameters | Subgroup | | No. of studies | SMD (95%*CI*) | *z* | p(*z*) | *I^2^* | p (Comparison) |
| --- | --- | --- | --- | --- | --- | --- | --- | --- |
| AST | Study type | Retrospective | 20 | 1.17 (0.80, 1.55) | 6.107 | <0.001 | 90.6 | 0.444 |
|  |  | Prospective | 7 | 1.39 (0.98, 1.80) | 6.567 | <0.001 | 65.4 |  |
| CK | Mean age (years) | <60 | 8 | 1.78 (0.72, 2.85) | 3.286 | 0.001 | 94.7 | 0.161 |
|  |  | 60-65 | 10 | 1.01 (0.58, 1.44) | 4.618 | <0.001 | 87.7 |  |
|  |  | >65 | 4 | 0.69 (0.22, 1.15) | 2.902 | 0.004 | 50.4 |  |
|  | Sample size | <50 | 8 | 2.70 (1.02, 4.38) | 3.145 | 0.002 | 94.8 | 0.022 |
|  |  | 50-100 | 5 | 1.69 (0.37, 3.01) | 2.513 | 0.012 | 94.2 |  |
|  |  | >100 | 9 | 0.69 (0.56, 0.81) | 11.154 | <0.001 | 0 |  |
|  | Study site | One | 16 | 1.28 (0.72, 1.83) | 4.534 | <0.001 | 92.8 | 0.060 |
|  |  | Two or more | 6 | 0.70 (0.45, 0.94) | 5.596 | <0.001 | 34.7 |  |
|  | Study type | Retrospective | 17 | 0.92 (0.52, 1.33) | 4.490 | <0.001 | 89.7 | 0.324 |
|  |  | Prospective | 5 | 1.64 (0.27, 3.02) | 2.342 | 0.019 | 93.1 |  |
| CK-MB | Mean age (years) | <60 | 3 | 0.30 (-0.31, 0.92) | 0.968 | 0.333 | 78.4 | 0.081 |
|  |  | 60-65 | 7 | 0.89 (0.66, 1.12) | 7.520 | <0.001 | 43.3 |  |
|  |  | >65 | 1 | 0.34 (-0.28, 0.96) | 1.088 | 0.276 | 0 |  |
|  | Sample size | <50 | 3 | 0.94 (0.44, 1.44) | 3.669 | <0.001 | 0 | 0.452 |
|  |  | 50-100 | 3 | 0.97 (0.30, 1.64) | 2.826 | 0.005 | 74.0 |  |
|  |  | >100 | 5 | 0.52 (0.20, 0.85) | 3.157 | <0.001 | 77.0 |  |
|  | Study site | One | 7 | 0.63 (0.22, 1.03) | 3.013 | <0.001 | 78.7 | 0.715 |
|  |  | Two or more | 4 | 0.80 (0.60, 1.00) | 7.914 | <0.001 | 0 |  |
|  | Study type | Retrospective | 9 | 0.64 (0.33, 0.96) | 3.961 | <0.001 | 72.4 | 0.296 |
|  |  | Prospective | 2 | 0.96 (0.46, 1.45) | 3.790 | <0.001 | 56.7 |  |
| sCr | Mean age (years) | <60 | 6 | 0.53 (-0.03, 1.10) | 1.851 | 0.064 | 83.0 | 0.540 |
|  |  | 60-65 | 9 | 0.80 (0.60, 1.00) | 7.870 | <0.001 | 44.9 |  |
|  |  | >65 | 4 | 0.59 (0.12, 1.06) | 2.461 | 0.014 | 46.7 |  |

| Laboratory parameters | Subgroup | | No. of studies | SMD (95%*CI*) | *z* | p(*z*) | *I^2^* | p (Comparison) |
| --- | --- | --- | --- | --- | --- | --- | --- | --- |
| sCr | Sample size | <50 | 8 | 0.50 (0.04, 0.97) | 2.112 | 0.035 | 57.6 | 0.603 |
|  |  | 50-100 | 3 | 0.72 (-0.06, 1.51) | 1.800 | 0.072 | 81.2 |  |
|  |  | >100 | 8 | 0.77 (0.54, 0.99) | 6.699 | <0.001 | 69.7 |  |
|  | Study site | One | 11 | 0.96 (0.47, 1.05) | 5.175 | <0.001 | 71.9 | 0.426 |
|  |  | Two or more | 8 | 0.60 (0.32, 0.88) | 4.255 | <0.001 | 53.1 |  |
|  | Study type | Retrospective | 15 | 0.75 (0.53, 0.97) | 6.736 | <0.001 | 63.8 | 0.332 |
|  |  | Prospective | 4 | 0.42(-0.21, 1.05) | 1.298 | 0.194 | 75.8 |  |
| LDH | Mean age (years) | <60 | 6 | 1.06 (0.46, 1.66) | 3.471 | 0.001 | 83.7 | 0.427 |
|  |  | 60-65 | 11 | 1.32 (0.94, 1.70) | 6.820 | <0.001 | 85.0 |  |
|  |  | >65 | 6 | 1.00 (0.69, 1.31) | 6.356 | <0.001 | 28.0 |  |
|  | Sample size | <50 | 7 | 0.92 (0.21, 1.62) | 2.531 | 0.011 | 77.2 | 0.011 |
|  |  | 50-100 | 7 | 1.82 (1.23, 2.40) | 6.082 | <0.001 | 80.1 |  |
|  |  | >100 | 9 | 0.88 (0.71, 1.06) | 9.788 | <0.001 | 50.1 |  |
|  | Study site | One | 16 | 1.23 (0.92, 1.53) | 4.070 | <0.001 | 78.3 | 0.372 |
|  |  | Two or more | 7 | 0.97 (0.50, 1.44) | 7.950 | <0.001 | 82.3 |  |
|  | Study type | Retrospective | 18 | 1.07 (0.84, 1.29) | 9.279 | <0.001 | 70.4 | 0.408 |
|  |  | Prospective | 5 | 1.62 (0.34, 2.90) | 2.475 | 0.013 | 91.7 |  |
| PLT | Mean age (years) | <60 | 8 | -0.43 (-0.80, -0.07) | -2.326 | 0.020 | 63.7 | 0.949 |
|  |  | 60-65 | 14 | -0.50 (-0.70, -0.29) | -4.810 | <0.001 | 61.6 |  |
|  |  | >65 | 7 | -0.50 (-0.74, -0.27) | -4.246 | <0.001 | 1.3 |  |
|  | Sample size | <50 | 10 | -0.46 (-0.90, -0.02) | -2.052 | 0.040 | 61.8 | 0.973 |
|  |  | 50-100 | 8 | -0.44 (-0.83, -0.05) | -2.213 | 0.027 | 68.0 |  |

| Laboratory parameters | Subgroup | | No. of studies | SMD (95%*CI*) | *z* | p(*z*) | *I^2^* | p (Comparison) |
| --- | --- | --- | --- | --- | --- | --- | --- | --- |
| PLT | Sample size | >100 | 11 | -0.49 (-0.62, -0.35) | -7.092 | <0.001 | 31.6 |  |
|  | Study site | One | 20 | -0.48 (-0.67, -0.29) | -4.991 | <0.001 | 59.8 | 0.701 |
|  |  | Two or more | 9 | -0.43 (-0.65, -0.21) | -3.786 | <0.001 | 34.6 |  |
|  | Study type | Retrospective | 23 | -0.47 (-0.65, -0.29) | -5.108 | <0.001 | 62.2 | 0.840 |
|  |  | Prospective | 6 | -0.50 (-0.69, -0.31) | -5.094 | <0.001 | 0 |  |
| PT | Mean age (years) | <60 | 4 | 0.77 (0.10, 1.44) | 2.261 | 0.024 | 56.4 | 0.546 |
|  |  | 60-65 | 6 | 0.77 (0.41, 1.12) | 4.193 | <0.001 | 72.9 |  |
|  |  | >65 | 2 | 0.48 (0.08, 0.88) | 2.370 | 0.018 | 20.5 |  |
|  | Sample size | <50 | 4 | 0.96 (0.06, 1.85) | 2.085 | 0.037 | 64.3 | 0.010 |
|  |  | 50-100 | 3 | 1.11 (0.75, 1.47) | 6.060 | <0.001 | 0 |  |
|  |  | >100 | 5 | 0.49 (0.28, 0.69) | 4.643 | <0.001 | 40.8 |  |
|  | Study site | One | 9 | 0.78 (0.47, 1.09) | 4.954 | <0.001 | 59.1 | 0.285 |
|  |  | Two or more | 3 | 0.51 (0.11, 0.90) | 2.526 | 0.012 | 57.1 |  |
|  | Study type | Retrospective | 9 | 0.81 (0.47, 1.15) | 4.652 | <0.001 | 67.4 | 0.090 |
|  |  | Prospective | 3 | 0.46 (0.25, 0.68) | 4.256 | <0.001 | 0 |  |
| TT | Mean age (years) | <60 | 3 | 2.02 (0.93, 3.12) | 3.614 | <0.01 | 91.8 | NA |
|  |  | 60-65 | 1 | 0.96 (0.38, 1.54) | 3.248 | 0.051 | NA |  |
|  |  | >65 | 1 | 0.88 (0.04, 1.72) | 2.057 | 0.040 | NA |  |
|  | Sample size | <50 | 2 | 2.40 (-0.62, 5.42) | 1.588 | 0.119 | 94.7 | 0.512 |
|  |  | 50-100 | 1 | 0.96 (0.38, 1.54) | 3.248 | 0.001 | NA |  |
|  |  | >100 | 2 | 1.28 (0.79, 1.77) | 5.094 | <0.001 | 65.4 |  |
|  | Study type | Retrospective | 4 | 0.95 (0.49, 1.40) | 4.059 | <0.001 | 77.8 | NA |
|  |  | Prospective | 1 | 3.96 (2.86, 5.06) | 7.043 | <0.001 | NA |  |
|  | Study site | One | 4 | 1.16 (0.85, 1.47) | 7.445 | <0.001 | 29.3 | NA |

| Laboratory parameters | Subgroup | | No. of studies | SMD (95%*CI*) | *z* | p(*z*) | *I^2^* | p (Comparison) |
| --- | --- | --- | --- | --- | --- | --- | --- | --- |
| TT | Study site | Two or more | 1 | 3.96 (2.86, 5.06) | 7.043 | <0.001 | NA |  |
| MON* | Mean age (years) | <60 | 3 | -0.14 (-0.81, 0.53) | -0.413 | 0.680 | 74.1 | 0.358 |
|  |  | 60-65 | 2 | -0.47 (-0.69, -0.26) | -4.274 | <0.001 | 0 |  |
|  | Sample size | <50 | 1 | -0.24 (-0.91, 0.43) | -0.707 | 0.480 | NA | 0.491 |
|  |  | 50-100 | 1 | -0.73 (-1.46, 0.01) | -1.945 | 0.052 | NA |  |
|  |  | >100 | 3 | -0.20 (-0.72, 0.32) | -0.752 | 0.452 | 85.5 |  |
|  | Study type | Retrospective | 3 | -0.14 (-0.81, 0.53) | -0.413 | 0.680 | 74.1 | 0.358 |
|  |  | Prospective | 2 | -0.47 (-0.69, -0.26) | -4.274 | <0.001 | 0 |  |
| LYM | Mean age (years) | <60 | 3 | -0.06 (-0.71, 0.60) | -0.177 | 0.859 | 73.1 | 0.441 |
|  |  | 60-65 | 5 | -0.33 (-0.56, -0.10) | -2.813 | 0.005 | 47.5 |  |
|  | Sample size | <50 | 1 | -0.11 (-0.78, 0.56) | -0.329 | 0.742 | NA | 0.912 |
|  |  | 50-100 | 2 | -0.31 (-0.93, 0.31) | -0.987 | 0.323 | 43.5 |  |
|  |  | >100 | 5 | -0.21 (-0.57, 0.14) | -1.172 | 0.241 | 80.4 |  |
|  | Study site | One | 7 | -0.18 (-0.51, 0.14) | -1.120 | 0.263 | 69.9 | 0.252 |
|  |  | Two or more | 1 | -0.43 (-0.69, -0.17) | -3.231 | 0.001 | NA |  |
|  | Study type | Retrospective | 6 | -0.12 (-0.48, 0.24) | -0.663 | 0.507 | 69.8 | 0.125 |
|  |  | Prospective | 2 | -0.45 (-0.67, -0.24) | -4.103 | <0.001 | 0 |  |
| TB^#^ | Sample size | <50 | 1 | 0.71 (-0.12, 1.53) | 1.679 | 0.093 | 0 | 0.657 |
|  |  | >100 | 4 | 0.50 (0.12, 0.89) | 2.556 | 0.011 | 79.8 |  |
|  | Study type | Retrospective | 4 | 0.36 (0.11, 0.61) | 2.868 | 0.004 | 22.6 | NA |
|  |  | Prospective | 1 | 0.92 (0.66, 1.19) | 6.818 | <0.001 | NA |  |
