# Supplementary material for: Clinical laboratory parameters and fatality of Severe fever with thrombocytopenia syndrome patients: A systematic review and meta-analysis
Source: PLoS Negl Trop Dis. 2022 Jun 17;16(6):e0010489. doi: 10.1371/journal.pntd.0010489 (PMC9246219; doi:10.1371/journal.pntd.0010489)
Supplement: S3 Text — ALB-albumin; ALT-alanine aminotransferase; APTT-activated partial-thromboplastin time; AST-creatin phosphokinase; CK-creatin phosphokinase; CK-MB-creatinine kinase myocardial b fraction; sCr-serum creatinine; LDH-lactate dehydrogenase; PLT-platelet count; PT-partial-thromboplastin time; Ref-reference unit; CI-confidence interval. (DOCX) [file pntd.0010489.s008.docx]

**Meta-regression analysis for factors (at least ten studies) with significant heterogeneity**

Table S1. Meta regression in estimates of ALB

| Covariate | Unit | Coefficient | 95%*CI* | p value | Residual *I^2^* |
| --- | --- | --- | --- | --- | --- |
| Study Type | Prospective | *Ref* |  |  |  |
|  | Retrospective | -0.323 | -0.835, 0.190 | 0.191 | 51.20% |
| Study site | Two or more | *Ref* |  |  |  |
|  | One | 0.226 | -0.311, 0.763 | 0.371 | 61.56% |
| Age (years) | <60 | *Ref* |  |  |  |
|  | 60-65 | 0.202 | -0.487, 0.892 | 0.523 |  |
|  | >65 | 0.003 | -0.735, 0.741 | 0.992 | 58.59% |
| Sample size | <50 | *Ref* |  |  |  |
|  | 50-100 | 0.658 | -0.599, 1.916 | 0.267 |  |
|  | >100 | 0.213 | -0.358, 0.785 | 0.421 | 63.98% |

Table S2. Meta regression in estimates of ALT

| Covariate | Unit | Coefficient | 95%*CI* | p value | Residual *I^2^* |
| --- | --- | --- | --- | --- | --- |
| Study Type | Prospective | *Ref* |  |  |  |
|  | Retrospective | 0.671 | -1.909, 3.251 | 0.594 | 85.97% |
| Study site | Two or more | *Ref* |  |  |  |
|  | One | 0.922 | -1.420, 3.264 | 0.422 | 86.00% |
| Age (years) | <60 | *Ref* |  |  |  |
|  | 60-65 | -1.082 | -4.103, 1.938 | 0.463 |  |
|  | >65 | -1.238 | -5.189, 2.714 | 0.521 | 86.82% |
| Sample size | <50 | *Ref* |  |  |  |
|  | 50-100 | 0.065 | -3.252, 3.383 | 0.968 |  |
|  | >100 | -0.999 | -3.864, 1.866 | 0.475 | 85.32% |

Table S3. Meta regression in estimates of APTT

| Covariate | Unit | Coefficient | 95%*CI* | p value | Residual *I^2^* |
| --- | --- | --- | --- | --- | --- |
| Study Type | Prospective | *Ref* |  |  |  |
|  | Retrospective | 1.026 | -0.322, 2.374 | 0.127 | 89.28% |
| Study site | Two or more | *Ref* |  |  |  |
|  | One | 0.715 | -0.526, 1.956 | 0.241 | 89.96% |
| Age (years) | <60 | *Ref* |  |  |  |
|  | 60-65 | -0.808 | -2.176, 0.561 | 0.229 |  |
|  | >65 | 0.057 | -1618, 1.733 | 0.943 | 89.91% |
| Sample size | <50 | *Ref* |  |  |  |
|  | 50-100 | -1.267 | -2.904, 0.369 | 0.120 |  |
|  | >100 | -1.060 | -2.509, 0.389 | 0.141 | 89.88% |

Table S4. Meta regression in estimates of AST

| Covariate | Unit | Coefficient | 95%*CI* | p value | Residual *I^2^* |
| --- | --- | --- | --- | --- | --- |
| Study Type | Prospective | *Ref* |  |  |  |
|  | Retrospective | 0.048 | -2.277, 2.373 | 0.967 | 88.65% |
| Study site | Two or more | *Ref* |  |  |  |
|  | One | 0.705 | -1.457, 2.868 | 0.304 | 89.47% |
| Age (years) | <60 | *Ref* |  |  |  |
|  | 60-65 | -0.360 | -2.852, 2.131 | 0.768 |  |
|  | >65 | -0.820 | -4.015, 2.376 | 0.602 | 89.47% |
| Sample size | <50 | *Ref* |  |  |  |
|  | 50-100 | 0.629 | -1.997, 3.255 | 0.626 |  |
|  | >100 | -0.382 | -2.787, 2.023 | 0.746 | 89.54% |

Table S5. Meta regression in estimates of CK

| Covariate | Unit | Coefficient | 95%*CI* | p value | Residual *I^2^* |
| --- | --- | --- | --- | --- | --- |
| Study Type | Prospective | *Ref* |  |  |  |
|  | Retrospective | -0.707 | -6.026, 4.621 | 0.786 | 90.65% |
| Study site | Two or more | *Ref* |  |  |  |
|  | One | 1.995 | -2.871, 6.862 | 0.403 | 90.70% |
| Age (years) | <60 | *Ref* |  |  |  |
|  | 60-65 | -2.659 | -7.562, 2.245 | 0.271 |  |
|  | >65 | -3.341 | -9.639, 2.958 | 0.281 | 91.05% |
| Sample size | <50 | *Ref* |  |  |  |
|  | 50-100 | -2.028 | -7.910, 3.855 | 0.479 |  |
|  | >100 | -3.126 | -8.150, 1.898 | 0.208 | 90.88% |

Table S6. Meta regression in estimates of CK-MB

| Covariate | Unit | Coefficient | 95%*CI* | p value | Residual *I^2^* |
| --- | --- | --- | --- | --- | --- |
| Study Type | Prospective | *Ref* |  |  |  |
|  | Retrospective | -0.343 | -1.151, 0.466 | 0.363 | 71.20% |
| Study sites | Two or more | *Ref* |  |  |  |
|  | One | -0.248 | -0.921, 0.426 | 0.427 | 71.15% |
| Age (years) | <60 | *Ref* |  |  |  |
|  | 60-65 | 0.624 | -0.003, 1.252 | 0.051 |  |
|  | >65 | 0.052 | -1.080, 1.184 | 0.918 | 59.67% |
| Sample size | <50 | *Ref* |  |  |  |
|  | 50-100 | 0.060 | -0.892, 1.012 | 0.888 |  |
|  | >100 | -0.391 | -1.218, 0.437 | 0.308 | 71.06% |

Table S7. Meta regression in estimates of sCr

| Covariate | Unit | Coefficient | 95%*CI* | p value | Residual *I^2^* |
| --- | --- | --- | --- | --- | --- |
| Study Type | Prospective | *Ref* |  |  |  |
|  | Retrospective | 0.290 | -0.328, 0.908 | 0.336 | 66.73% |
| Study site | Two or more | *Ref* |  |  |  |
|  | One | 0.183 | -0.318, 0.685 | 0.451 | 66.31% |
| Age (years) | <60 | *Ref* |  |  |  |
|  | 60-65 | 0.216 | -0.373, 0.804 | 0.448 |  |
|  | >65 | 0.014 | -0.750, 0.777 | 0.970 | 67.73% |
| Sample size | <50 | *Ref* |  |  |  |
|  | 50-100 | 0.205 | -0.590, 1.000 | 0.593 |  |
|  | >100 | 0.248 | -0.334, 0.831 | 0.380 | 68.17% |

Table S8. Meta regression in estimates of LDH

| Covariate | Unit | Coefficient | 95%*CI* | p value | Residual *I^2^* |
| --- | --- | --- | --- | --- | --- |
| Study Type | Prospective | *Ref* |  |  |  |
|  | Retrospective | -0.435 | -1.435, 0.565 | 0.376 | 80.18% |
| Study site | Two or more | *Ref* |  |  |  |
|  | One | 0.283 | -0.559, 1.125 | 0.466 | 79.62% |
| Age (years) | <60 | *Ref* |  |  |  |
|  | 60-65 | 0.257 | -0.721, 1.234 | 0.590 |  |
|  | >65 | -0.173 | -1.292, 0.946 | 0.750 | 80.82% |
| Sample size | <50 | *Ref* |  |  |  |
|  | 50-100 | 0.950 | 0.050, 1.851 | 0.040 |  |
|  | >100 | 0.046 | -0.770, 0.863 | 0.907 | 72.40% |

Table S9. Meta regression in estimates of PLT

| Covariate | Unit | Coefficient | 95%*CI* | p value | Residual *I^2^* |
| --- | --- | --- | --- | --- | --- |
| Study Type | Prospective | *Ref* |  |  |  |
|  | Retrospective | 0.004 | -0.440, 0.447 | 0.987 | 55.29% |
| Study site | Two or more | *Ref* |  |  |  |
|  | One | -0.064 | -0.438, 0.311 | 0.729 | 54.63% |
| Age (years) | <60 | *Ref* |  |  |  |
|  | 60-65 | -0.105 | -0.539, 0.330 | 0.625 |  |
|  | >65 | -0.079 | -0.616, 0.458 | 0.765 | 56.07% |
| Sample size | <50 | *Ref* |  |  |  |
|  | 50-100 | -0.065 | -0.611, 0.481 | 0.809 |  |
|  | >100 | -0.072 | -0.541, 0.398 | 0.756 | 56.72% |

Table S10. Meta regression in estimates of PT

| Covariate | Unit | Coefficient | 95%*CI* | p value | Residual *I^2^* |
| --- | --- | --- | --- | --- | --- |
| Study Type | Prospective | *Ref* |  |  |  |
|  | Retrospective | 0.292 | -0.418, 1.002 | 0.381 | 61.58% |
| Study site | Multi | *Ref* |  |  |  |
|  | One | 0.243 | -0.452, 0.937 | 0.454 | 58.68% |
| Age (years) | <60 | *Ref* |  |  |  |
|  | 60-65 | 0.017 | -0.849, 0.882 | 0.966 |  |
|  | >65 | -0.235 | -1.328, 0.859 | 0.639 | 66.17% |
| Sample size | <50 | *Ref* |  |  |  |
|  | 50-100 | 0.248 | -0.688, 1.185 | 0.564 |  |
|  | >100 | -0.370 | -1.179, 0.439 | 0.328 | 47.49% |

Table S11. Meta regression in estimates of viral load

| Covariate | Unit | Coefficient | 95%*CI* | p value | Residual *I^2^* |
| --- | --- | --- | --- | --- | --- |
| Study Type | Prospective | *Ref* |  |  |  |
|  | Retrospective | -0.231 | -1.758, 1.296 | 0.736 | 81.46% |
| Study site | Multi | *Ref* |  |  |  |
|  | One | 0.0566 | -1.356, 1.470 | 0.929 | 81.61% |
| Age (years) | <60 | *Ref* |  |  |  |
|  | 60-65 | 0.538 | -1.134, 2.210 | 0.472 |  |
|  | >65 | -0.049 | -1.985, 1.887 | 0.954 | 82.25% |
| Sample size | <50 | *Ref* |  |  |  |
|  | 50-100 | 0.546 | -1.243, 2.335 | 0.494 |  |
|  | >100 | -0.011 | -1.736, 1.713 | 0.988 | 83.00% |
