# Supplementary material for: Clinical laboratory parameters and fatality of Severe fever with thrombocytopenia syndrome patients: A systematic review and meta-analysis
Source: PLoS Negl Trop Dis. 2022 Jun 17;16(6):e0010489. doi: 10.1371/journal.pntd.0010489 (PMC9246219; doi:10.1371/journal.pntd.0010489)
Supplement: S4 Text — ALB-albumin; ALT-alanine aminotransferase; APTT-activated partial-thromboplastin time; AST-creatin phosphokinase; CK-creatin phosphokinase; CK-MB-creatinine kinase myocardial b fraction; sCr-serum creatinine; FIB-fibrinogen; LDH-lactate dehydrogenase; MON-monocyte; PLT-platelet count; PT-partial-thromboplastin time; TB-total bilirubin; TT-thrombin time; LYM-lymphocyte. (DOCX) [file pntd.0010489.s009.docx]

**Sensitivity analysis for laboratory parameters with significant heterogeneity**

S1 Fig. Sensitivity analysis of the literature related to ALB


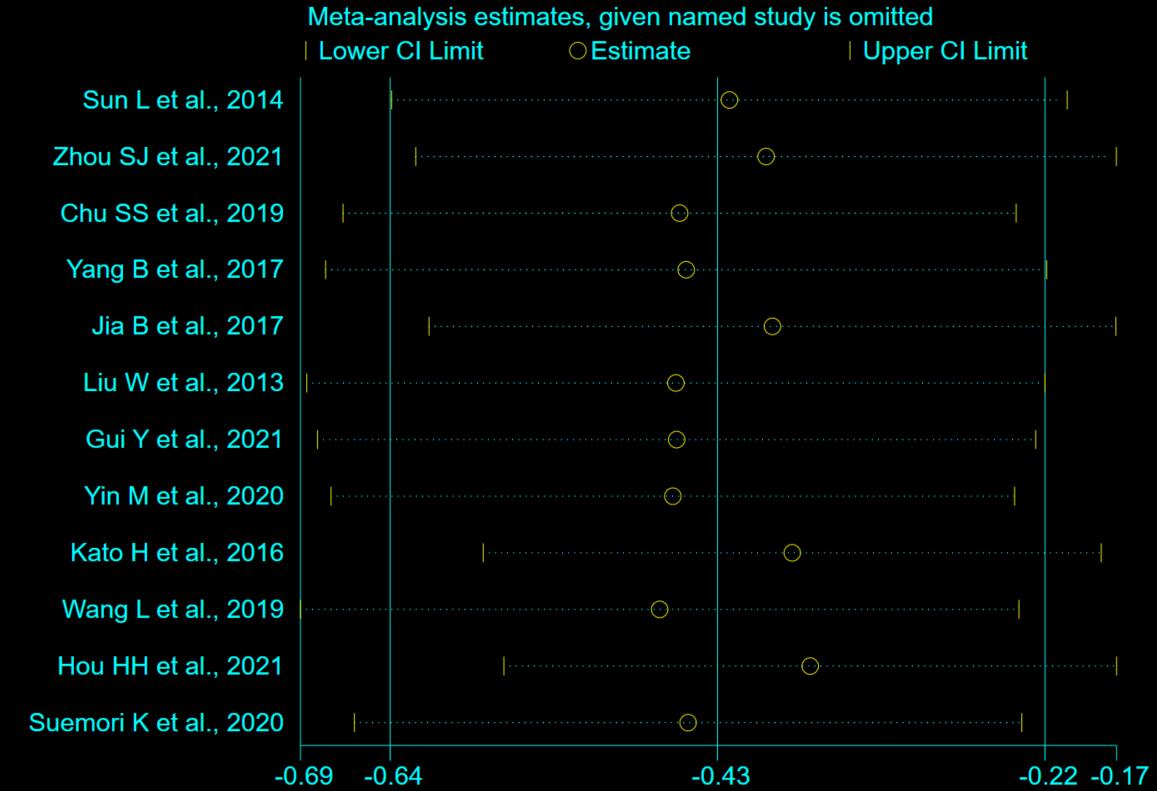


S2 Fig. Sensitivity analysis of the literature related to ALT


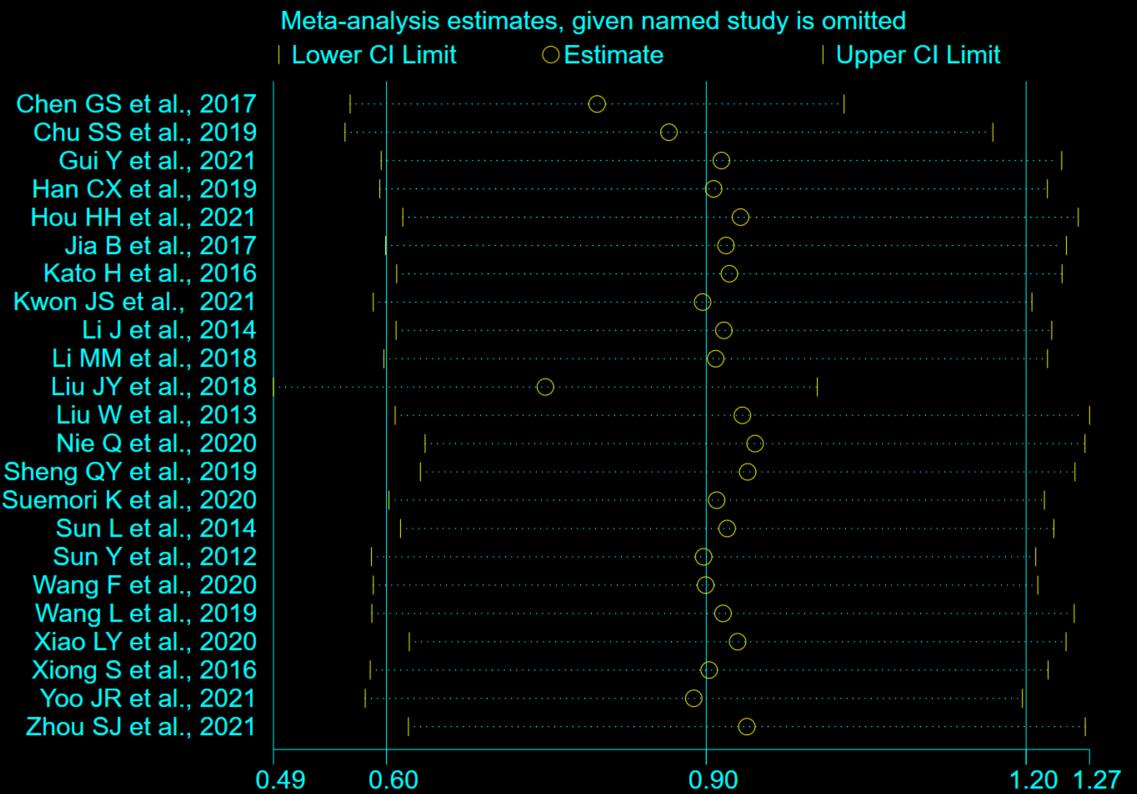


S3 Fig. Sensitivity analysis of the literature related to APTT


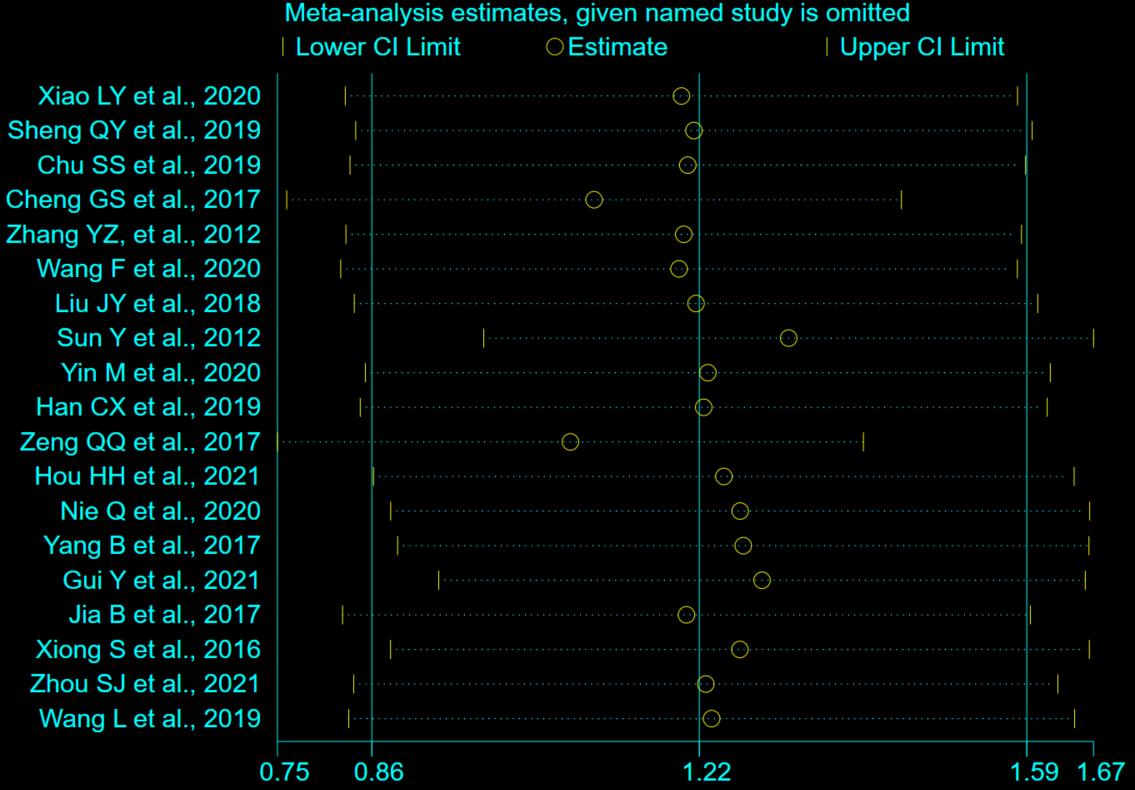


S4 Fig. Sensitivity analysis of the literature related to AST


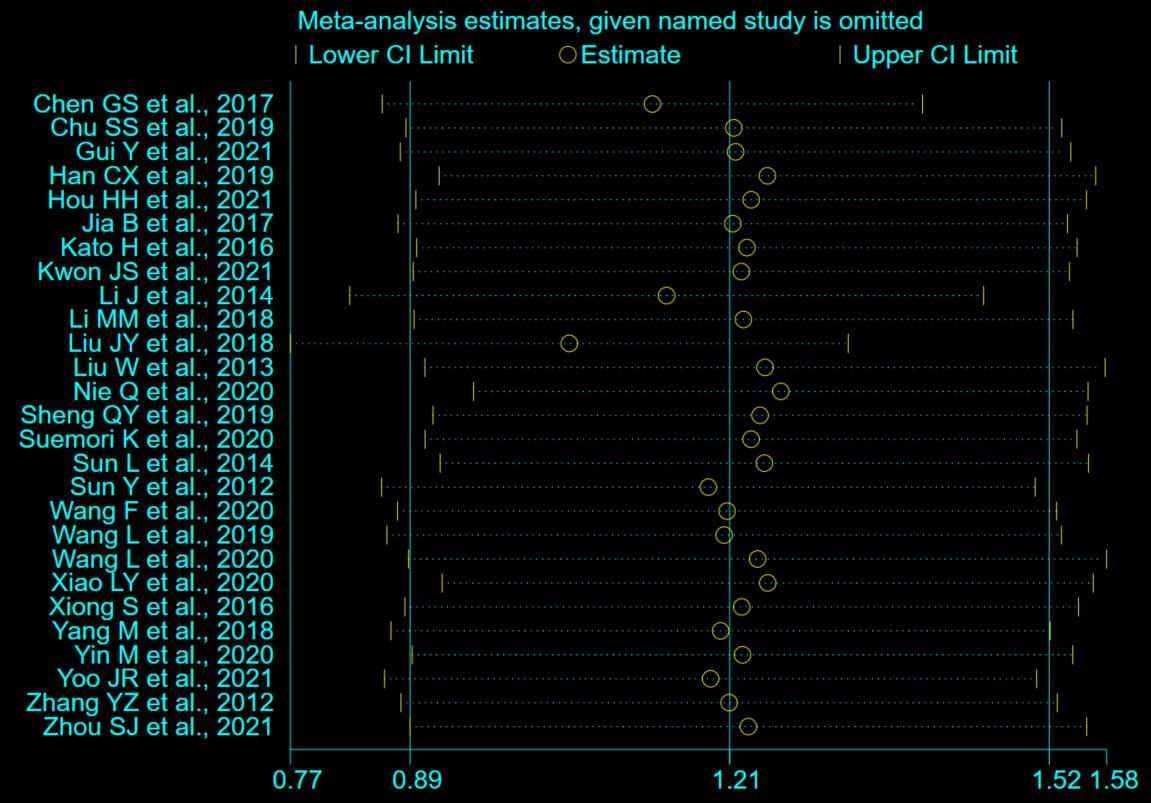


S5 Fig. Sensitivity analysis of the literature related to CK


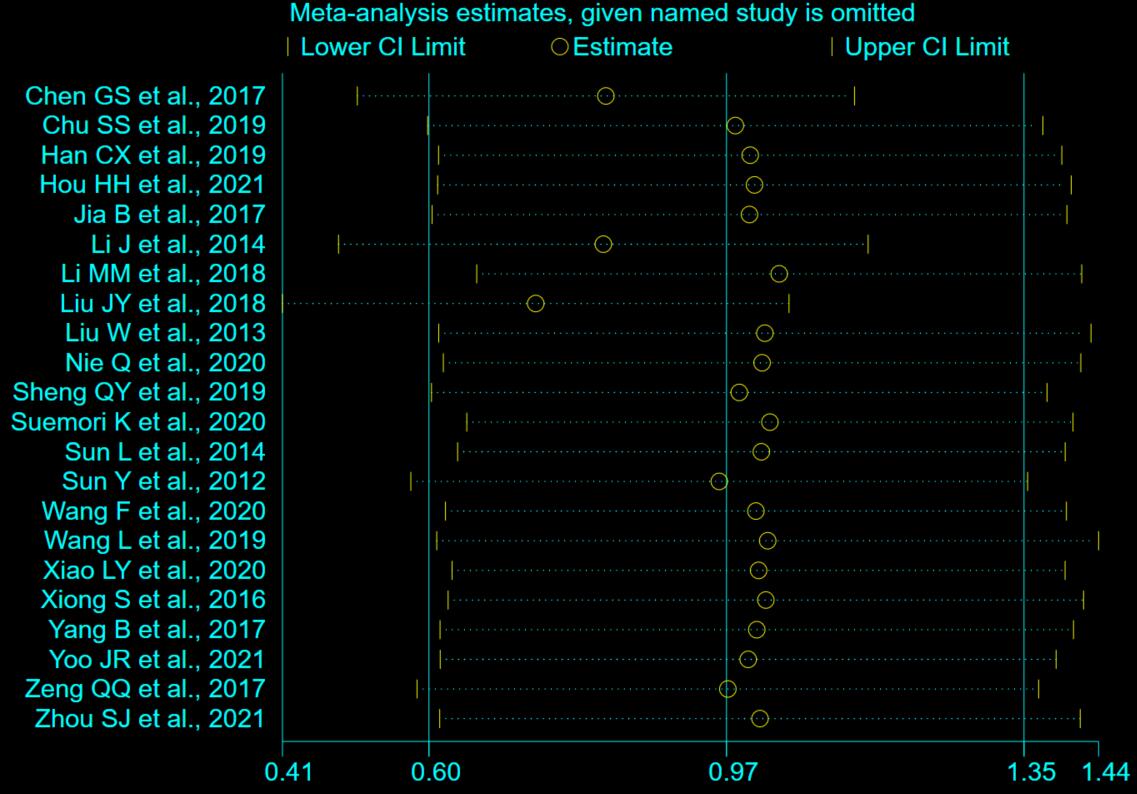


S6 Fig. Sensitivity analysis of the literature related to CK-MB


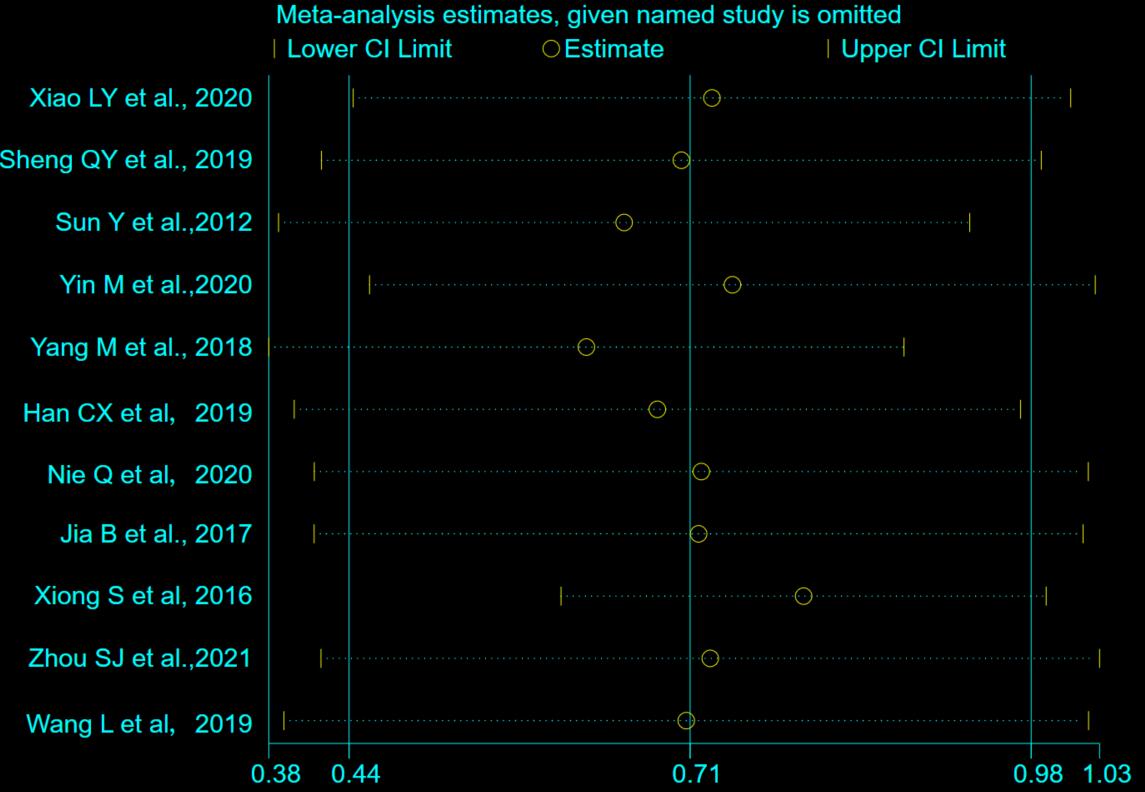


S7 Fig. Sensitivity analysis of the literature related to sCr


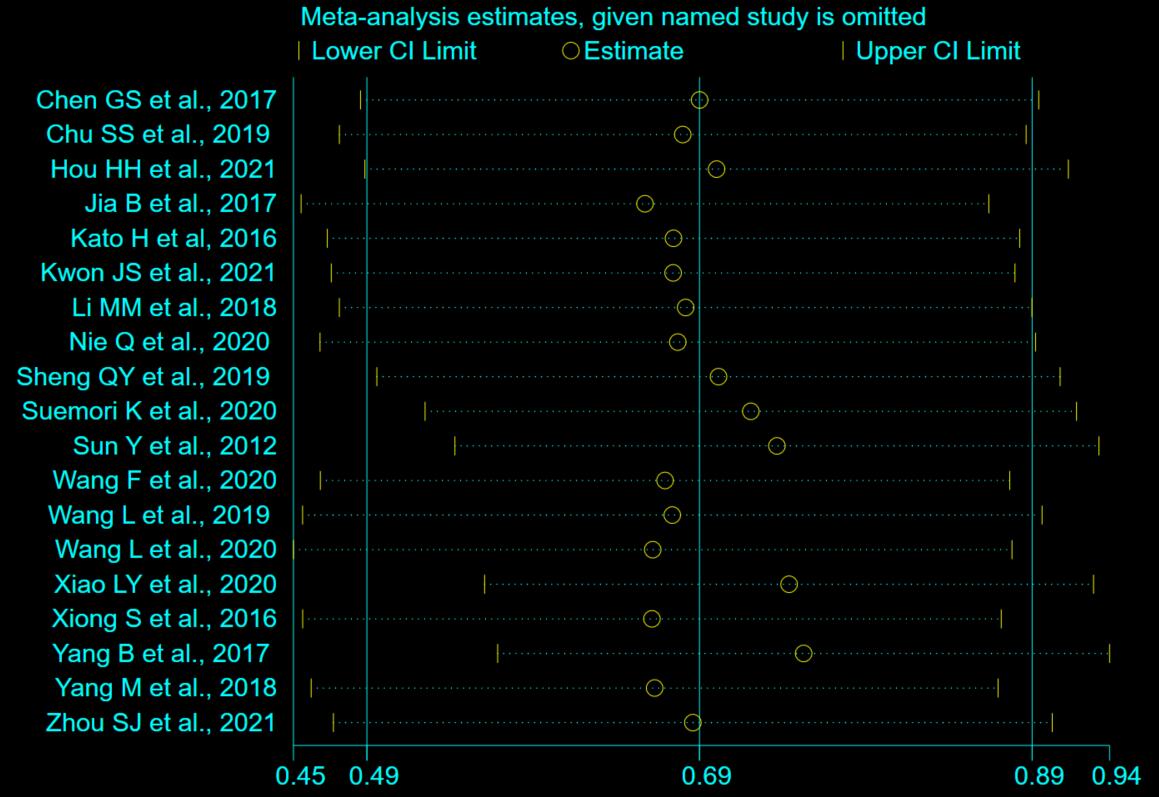


S8 Fig. Sensitivity analysis of the literature related to FIB


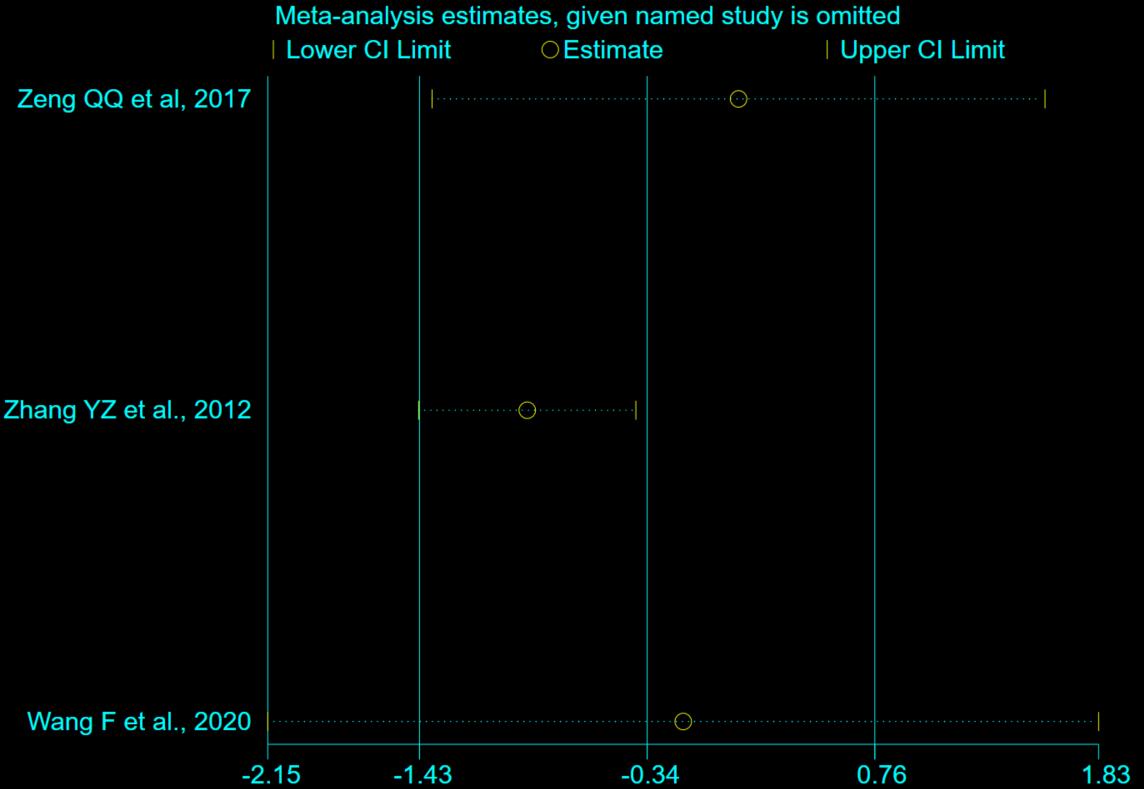


S9 Fig. Sensitivity analysis of the literature related to LDH


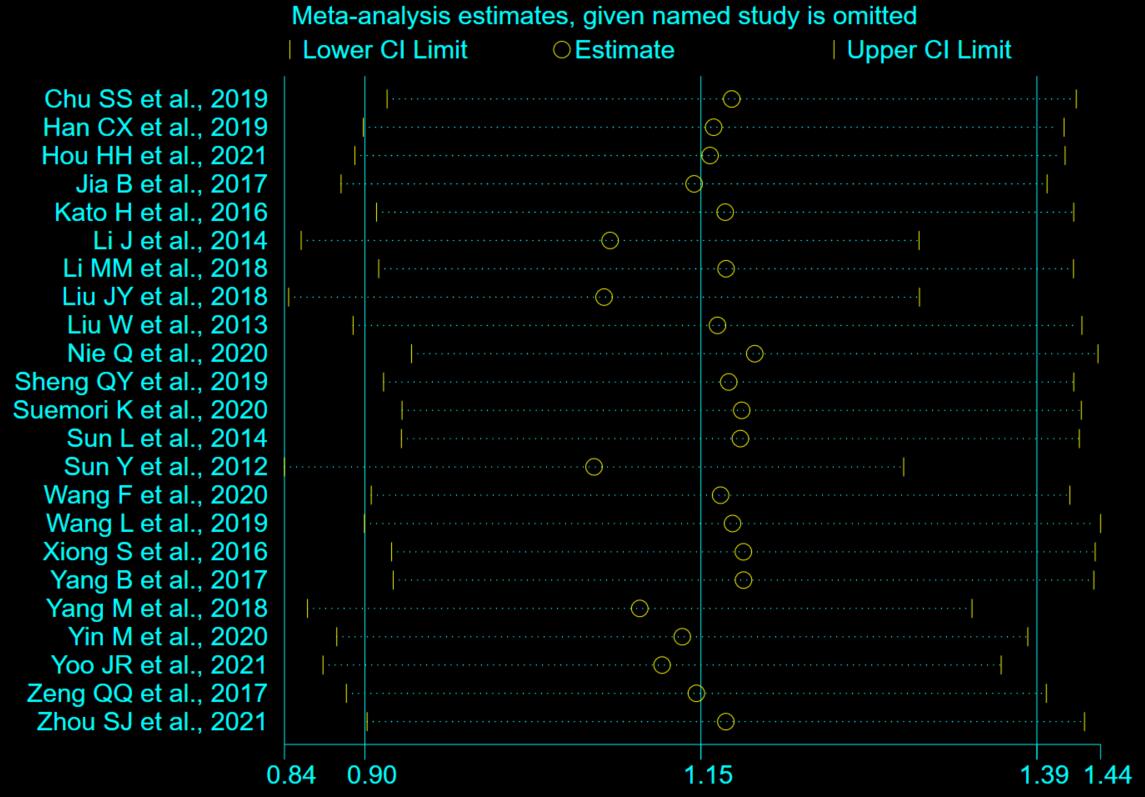


S10 Fig. Sensitivity analysis of the literature related to MON


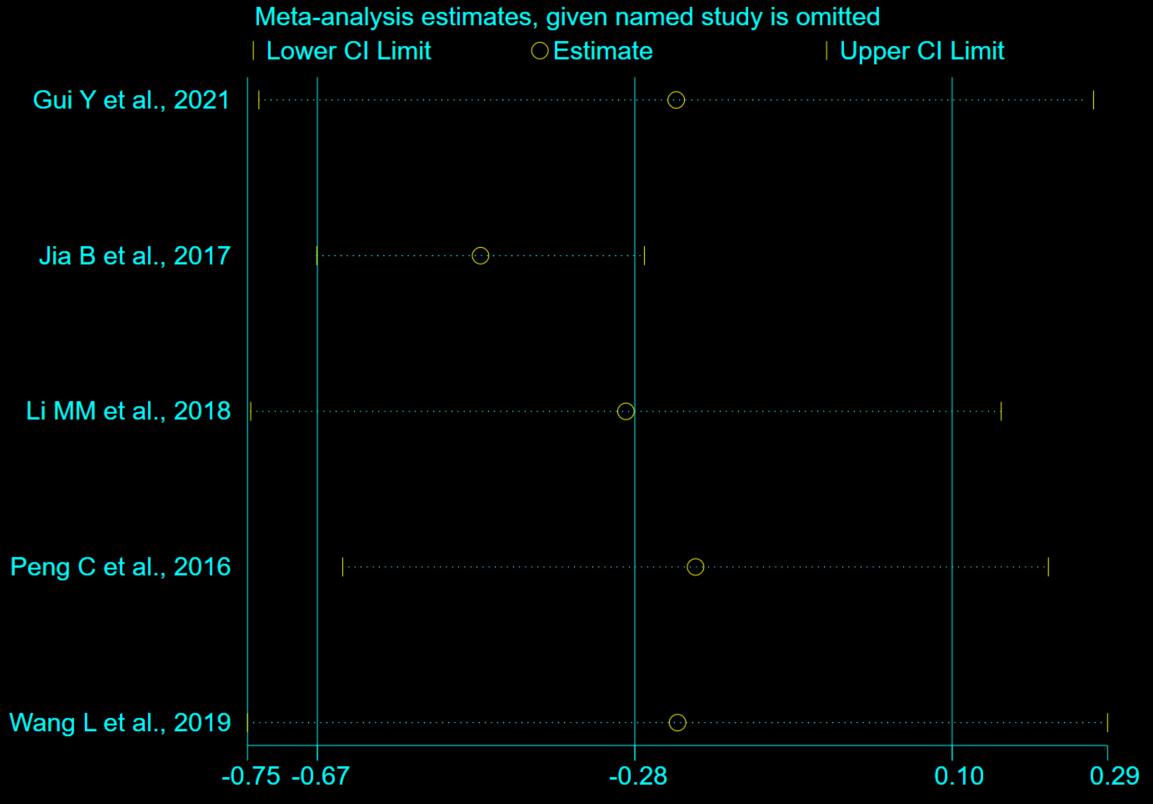


S11 Fig. Sensitivity analysis of the literature related to PLT


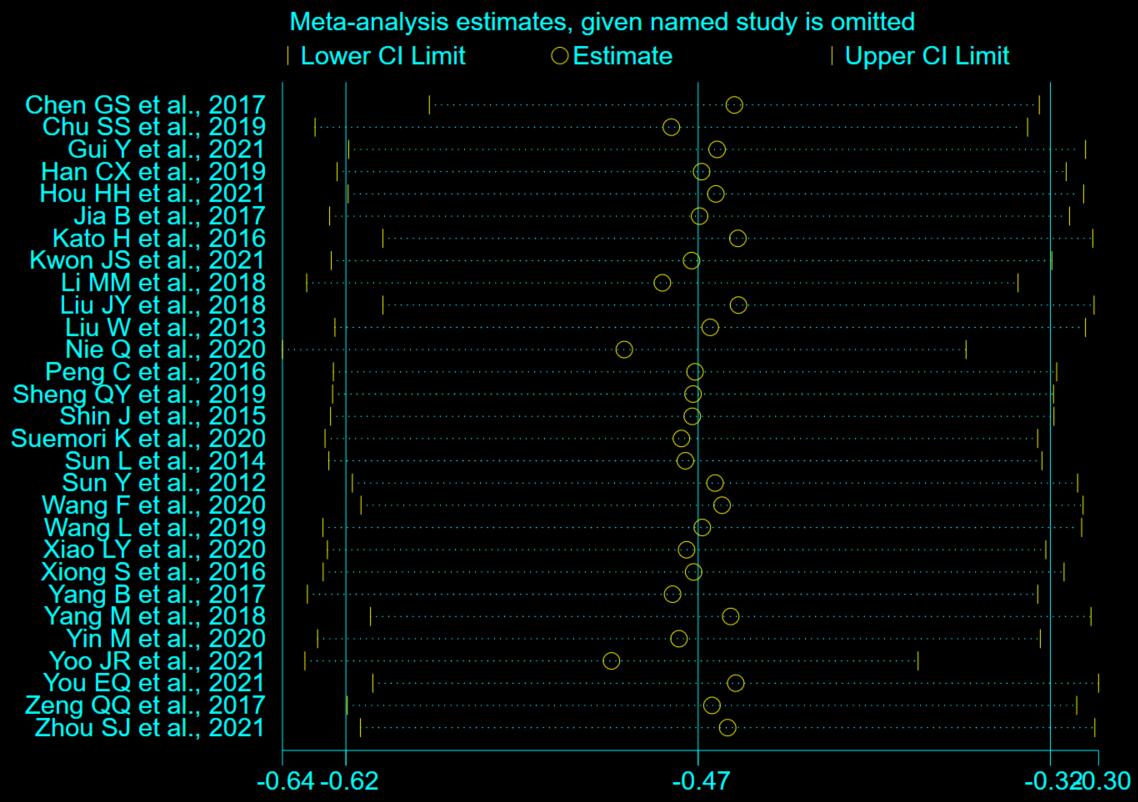


S12 Fig. Sensitivity analysis of the literature related to PT


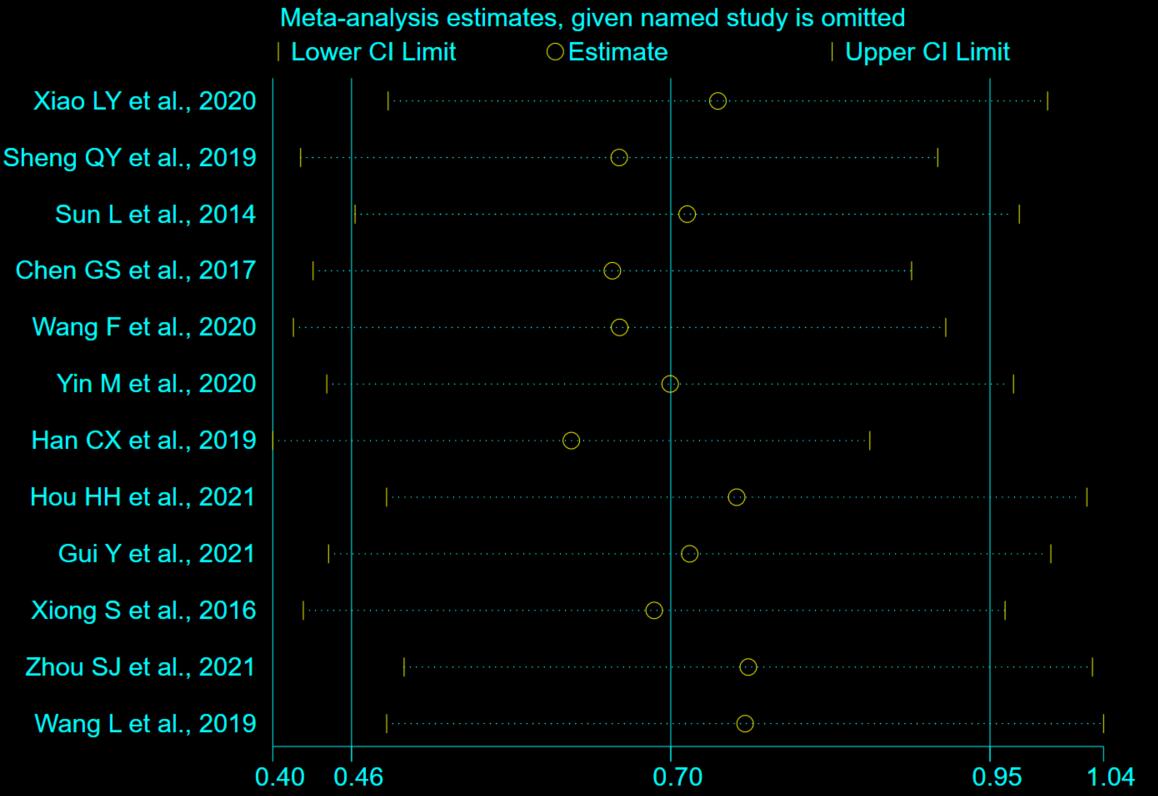


S13 Fig. Sensitivity analysis of the literature related to TB


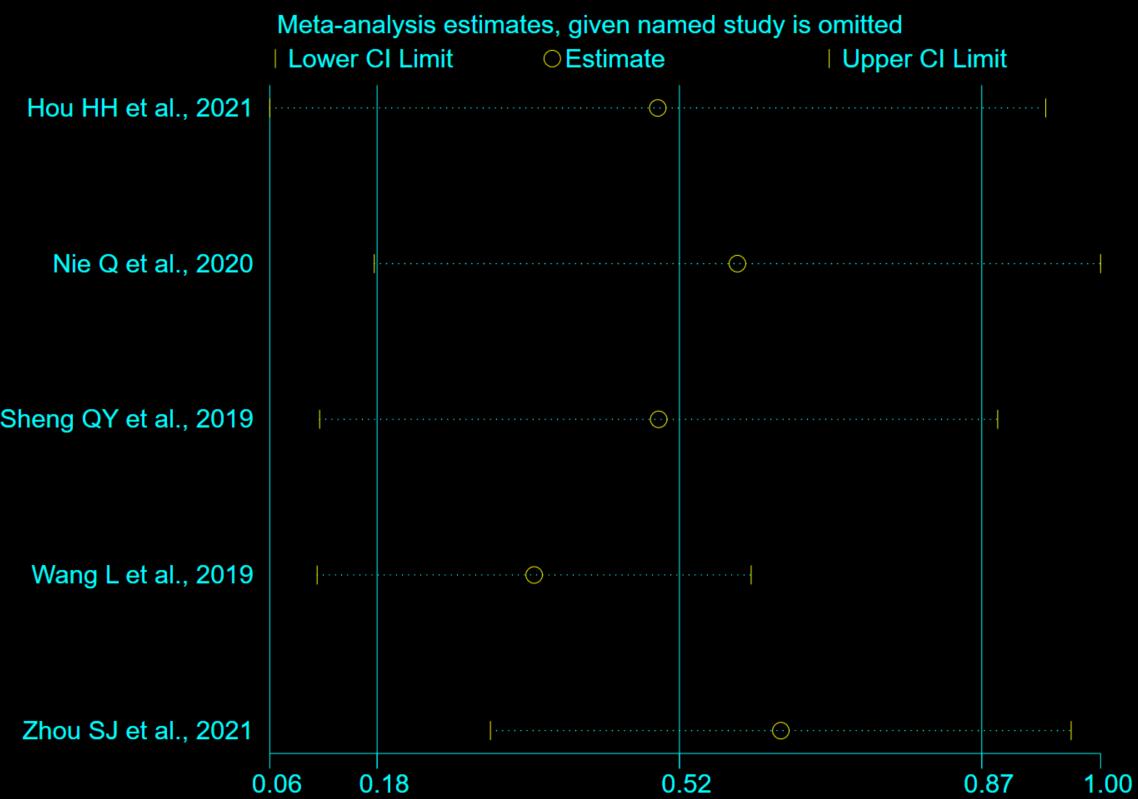


S14 Fig. Sensitivity analysis of the literature related to TT


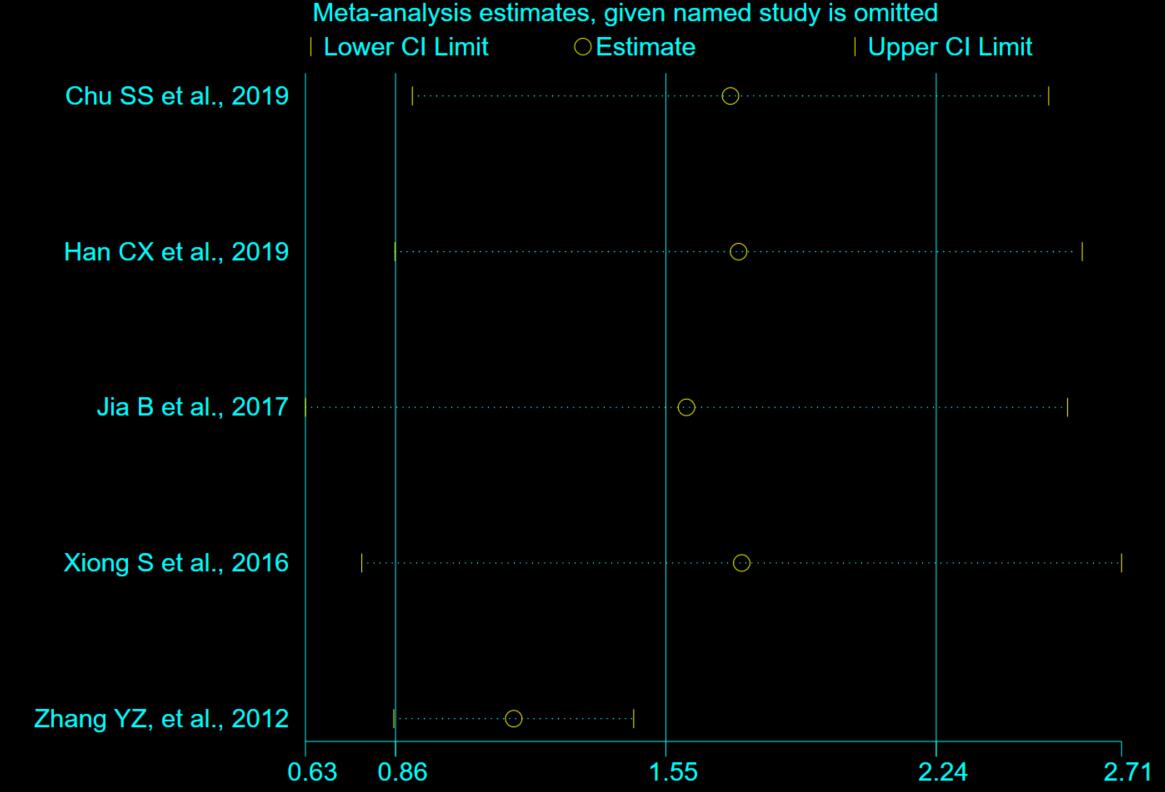


S15 Fig. Sensitivity analysis of the literature related to viral load


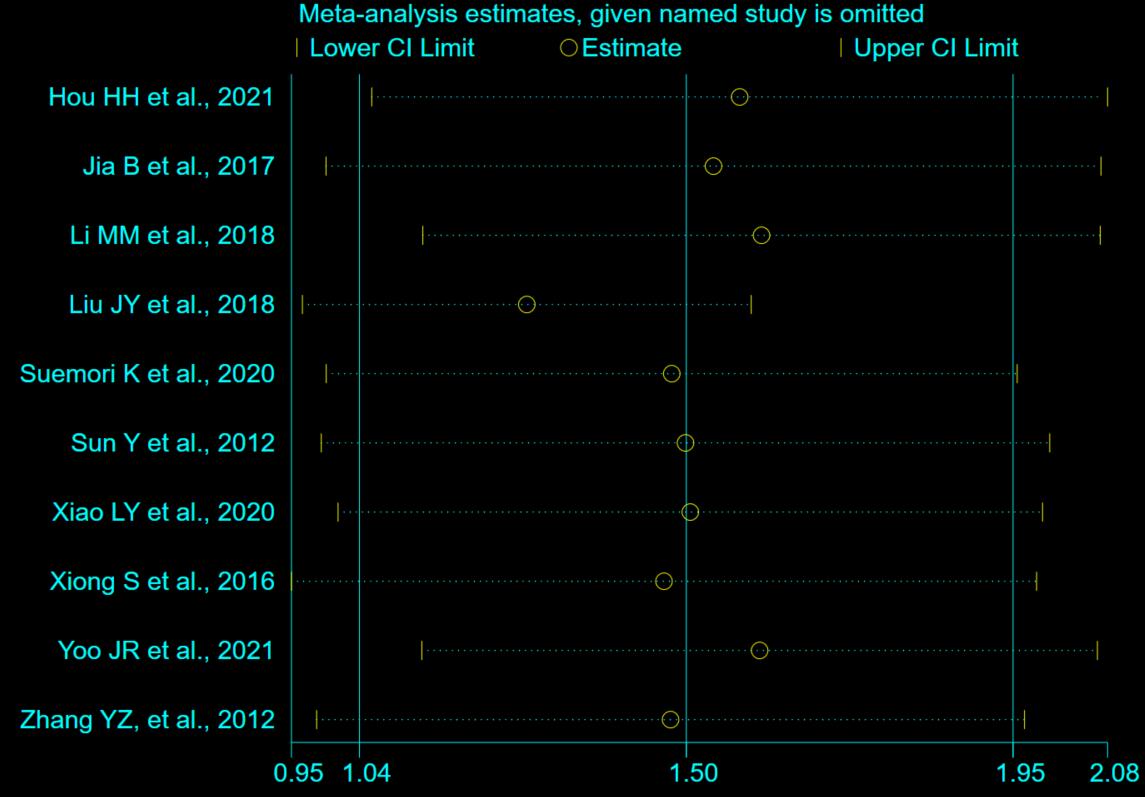


S16 Fig. Sensitivity analysis of the literature related to LYM


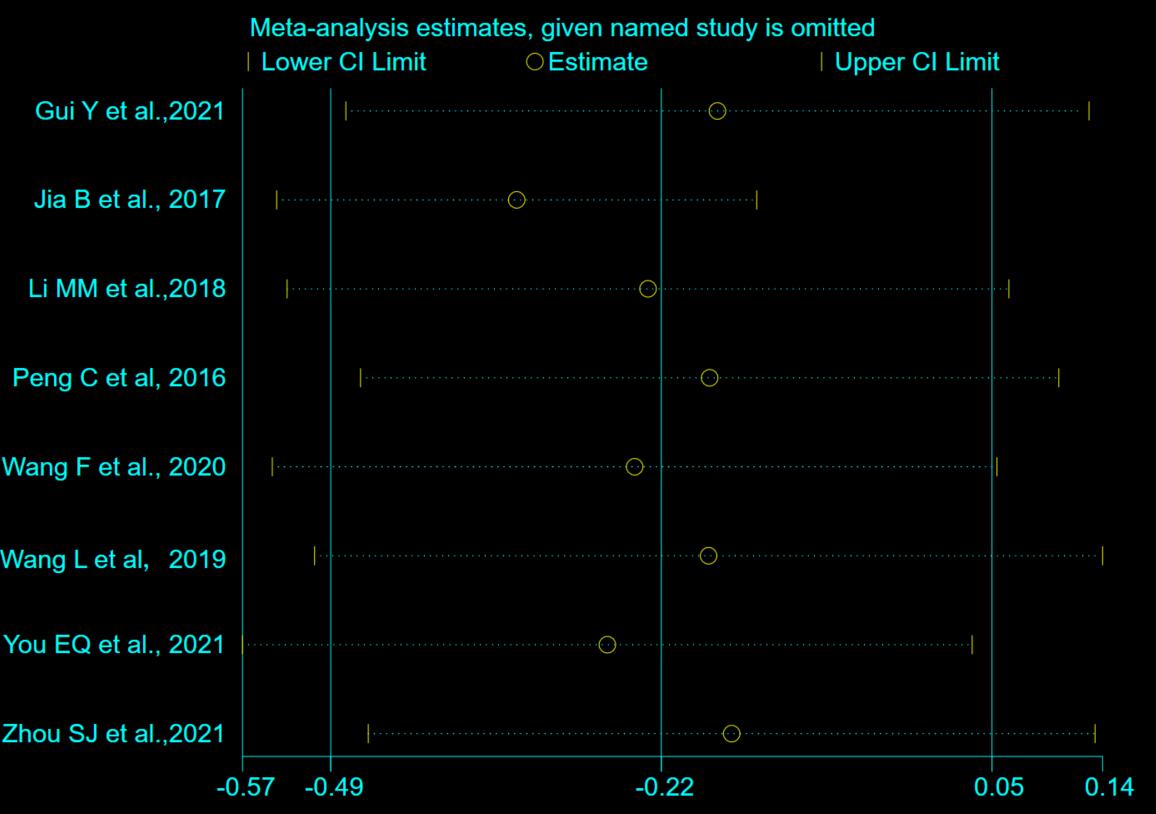


**References** (listed in alphabetical order)

Chen GS, Hu LF, Xu XH, Li JB. The clinical characteristics and prognostic indicators of severe fever with thrombocytopenia syndrome infected by new Bunia virus. China Medical Equipment. 2017; 14(5): 94-97. DOI: 10.3969/J.ISSN.1672-8270.2017.05.025.

Chu SS, Zheng W, Wang JW, Chen YJ, Yu L, Xu CD, et al. Clinical characteristics of 40 patients with severe fever with thrombocytopenia syndrome in Tiantai County. Inter J Epidemiol Infect Dis. 2019; 46(6): 526-529. DOI: 10.3760/cma.j.issn.1673-4149.2019.06.016.

Gui Y, Xu Y, Yang P. Predictive Value of the Platelet-to-Albumin Ratio (PAR) on the Risk of Death at Admission in Patients Suffering from Severe Fever with Thrombocytopenia Syndrome. J Inflamm Res. 2021; 14: 5647-5652. DOI: 10.2147/JIR.S335727.

Han CX, Sun AJ, Pu CW, Li YT, Sui F, Qin SJ, et al. Epidemiological characteristics of severe fever with thrombocytopenia syndrome caused by novel bunyavirus infection and influencing factors for prognosis. Chin J Nosocomiol. 2019; 29(2):171-174+187. DOI: 10.11816/cn.ni.2019-180685.

Hou HH, Mao LL, Liang HY, Liu Y, Liu XS, Deng BC. Clinical characteristics and influencing factors for prognosis of fever with severe thrombocytopenia syndrome in Dalian, Liaoning Province. Chin J Infect Control. 2021; 20(10): 897-902. DOI: 10.12138/j.issn.1671-9638.20218284.

Jia B, Yan X, Chen Y, Wang G, Liu Y, Xu B, et al. A scoring model for predicting prognosis of patients with severe fever with thrombocytopenia syndrome. PLoS Negl Trop Dis. 2017; 11(9): e0005909. DOI: 10.1371/journal.pntd.0005909.

Kato H, Yamagishi T, Shimada T, Matsui T, Shimojima M, Saijo M, et al. SFTS epidemiological research group-Japan. Epidemiological and Clinical Features of Severe Fever with Thrombocytopenia Syndrome in Japan, 2013-2014. PLoS One. 2016; 11(10): e0165207. DOI: 10.1371/journal.pone.0165207.

Kwon JS, Jin S, Kim JY, Ra SH, Kim T, Park SY, et al. Viral and Immunologic Factors Associated with Fatal Outcome of Patients with Severe Fever with Thrombocytopenia in Korea. Virus. 2021; 13(12): 2351. DOI: 10.3390/v13122351.

Li J, Han Y, Xing Y, Li S, Kong L, Zhang Y, et al. Concurrent measurement of dynamic changes in viral load, serum enzymes, T cell subsets, and cytokines in patients with severe fever with thrombocytopenia syndrome. PLoS One. 2014; 9(3): e91679. DOI: 10.1371/journal.pone.0091679.

Li MM, Zhang WJ, Weng XF, Li MY, Liu J, Xiong Y, et al. CD4 T cell loss and Th2 and Th17 bias are associate with the severity of severe fever with thrombocytopenia syndrome (SFTS). Clin Immunol. 2018; 195: 8-17. DOI: 10.1016/j.clim.2018.07.009.

Liu JY, Feng J, Li AL, Wang SY, Zheng R, Chen HZ. Analysis of clinical characteristics and death risk factors in patients infected with severe fever with thrombocytopenia syndrome bunyavirus. Chin J Postgrad Med, 2018; 41(5):429-433. DOI: 10.3760/cma.j.issn.1673-4904.2018.05.012.

Liu W, Lu QB, Cui N, Li H, Wang LY, Liu K, et al. Case-fatality ratio and effectiveness of ribavirin therapy among hospitalized patients in china who had severe fever with thrombocytopenia syndrome. Clin Infect Dis. 2013; 57(9):1292-9. DOI: 10.1093/cid/cit530.

Nie Q, Wang D, Ning Z, Li T, Tian X, Bian P, et al. Analysis of Severe Fever With Thrombocytopenia Syndrome in Critical Ill Patients in Central China. Shock. 2020; 54(4):451-457. DOI: 10.1097/SHK.0000000000001527.

Peng C, Wang H, Zhang W, Zheng X, Tong Q, Jie S, et al. Decreased monocyte subsets and TLR4-mediated functions in patients with acute severe fever with thrombocytopenia syndrome (SFTS). Int J Infect Dis. 2016; 43:37-42. DOI: 10.1016/j.ijid.2015.12.009.

Sheng QY, Sheng JF, Zhang X, Ye WW, Huang HJ. Clinical characteristics and prognostic factors of 25 patients with new bunyavirus infection. Chin J Crit Care Med (Electronic Edition) 2019; 12: 152-157. DOI: 10.3877/cma.j.issn.1674-6880.2019.03.002.

Shin J, Kwon D, Youn SK, Park JH. Characteristics and Factors Associated with Death among Patients Hospitalized for Severe Fever with Thrombocytopenia Syndrome, South Korea, 2013. Emerg Infect Dis. 2015; 21:1704-1710. DOI:10.3201/eid2110.141928.

Suemori K, Saijo M, Yamanaka A, Himeji D, Kawamura M, Haku T, et al. A multicenter non-randomized, uncontrolled single arm trial for evaluation of the efficacy and the safety of the treatment with favipiravir for patients with severe fever with thrombocytopenia syndrome. PLoS Negl Trop Dis. 2021; 15(2): e0009103. DOI: 10.1371/journal.pntd.0009103.

Sun L, Hu Y, Niyonsaba A, Tong Q, Lu L, Li H, et al. Detection and evaluation of immunofunction of patients with severe fever with thrombocytopenia syndrome. Clin Exp Med. 2014; 14(4):389-95. DOI: 10.1007/s10238-013-0259-0.

Sun Y, Jin C, Zhan F. Wang X, Lian M, Zhang Q, et al. Host cytokine storm is associated with disease severity of severe fever with thrombocytopenia syndrome. J Infect Dis. 2012; 206(7): 1085-94. DOI: 10.1093/infdis/jis452.

Wang F, Wu Y, Jiao J, Wang J, Ge Z. Risk Factors and Clinical Characteristics of Severe Fever with Thrombocytopenia Syndrome. Int J Gen Med. 2020; 13: 1661-1667. DOI: 10.2147/IJGM.S292735.

Wang L, Wan G, Shen Y, Zhao Z, Lin L, Zhang W, et al. A nomogram to predict mortality in patients with severe fever with thrombocytopenia syndrome at the early stage-A multicenter study in China. PLoS Negl Trop Dis. 2019; 13(11): e0007829. DOI: 10.1371/journal.pntd.0007829.

Wang L, Zou Z, Ding K, Hou C. Predictive risk score model for severe fever with thrombocytopenia syndrome mortality based on qSOFA and SIRS scoring system. BMC Infect Dis. 2020; 20(1): 595. DOI: 10.1186/s12879-020-05299-7.

Xiao LY, Shi DY, Liu YF, Zheng YS. Clinical characteristics and treatment efficacy of sever infection caused by new bunyaviridae. Electronic Journal of Emerging Infectious Diseases. 2020; 5(1): 16-19. DOI: 0.19871/j.cnki.xfcrbzz.2020.01.003.

Xiong S, Zhang W, Li M, Xiong Y, Li M, Wang H, et al. A simple and practical score model for predicting the mortality of severe fever with thrombocytopenia syndrome patients. Medicine (Baltimore). 2016; 95(52): e5708. DOI: 10.1097/MD.0000000000005708.

Yang B, Wang X, Li Y, Wu A, Liu Q, Lu Y, et al. A Newly Established Severity Scoring System in Predicting the Prognosis of Patients with Severe Fever with Thrombocytopenia Syndrome. Tohoku J Exp Med. 2017; 242(1): 19-25. DOI: 10.1620/tjem.242.19.

Yang M, Ye J, Li H, Hua TF, Zheng Y, Li J. Investigation of clinical characteristics and prognosis of severe fever with thrombocytopenia syndrome: 69 cases analysis. Chin J Dis Control Prev. 2018; 22(4): 402-405. DOI: 10.16462/j.cnki.zhjbkz.2018.04.018.

Yin M, Zhao ZH, Yang Y. Risk factors for death in 95 patients with fever and thrombocytopenia syndrome. Journal of Anhui Health Vocational & Technical College. 2020; 19(4): 21-23+25. DOI: 10.3969/j.issn.1671-8054.2020.04.009.

Yoo JR, Kim TJ. Heo ST, Hwang KA, Oh H, Ha T, et al. IL-6 and IL-10 Levels, Rather Than Viral Load and Neutralizing Antibody Titers, Determine the Fate of Patients With Severe Fsver With Thrombocytopenia Syndrome Virus Infection in South Korea. Front Immunol. 2021; 12:711847. DOI: 10.3389/fimmu.2021.711847.

You EQ, Wang L, Zhang L, Wu J, Zhao K, Huang F. Epidemiological characteristics of severe fever with thrombocytopenia syndrome in surveillance study from 2011 to 2018. Eur J Clin Microboil Infect Dis. 2021; 40(5): 929-939. DOI: 10.1007/s10096-020-04098-x.

Zeng QQ, Wang QJ, Zhang JJ, Yang ZJ, Li YC, Zhu HM, et al. Risk factors for mortality in patients with severe fever with thrombocytopenia syndrome. Chin J Infect Dis. 2017; 35(6):336-340. DOI: 10.3760/cma.j.issn.1000-6680.2017.06.004.

Zhang YZ, He YW, Dai YA, Xiong Y, Zheng H, Zhou DJ, et al. Hemorrhagic fever caused by a novel Bunyavirus in China: pathogenesis and correlates of fatal outcome. Clin Infect Dis. 2012; 54(4):527-33. DOI:10.1093/cid/cir804.

Zhou SJ, Xia GM, He TF, Xu MY, Ye J, Li X, et al. Clinical characteristics and prognostic factors of patients infected with novel Bunyavirus. Acta Universitatis Medicinalis Anhui. 2021; 56(6): 942-947. DOI: 10.19405/j.cnki.issn1000-1492.2021.06.020.
