# Supplementary material for: Clinical laboratory parameters and fatality of Severe fever with thrombocytopenia syndrome patients: A systematic review and meta-analysis
Source: PLoS Negl Trop Dis. 2022 Jun 17;16(6):e0010489. doi: 10.1371/journal.pntd.0010489 (PMC9246219; doi:10.1371/journal.pntd.0010489)
Supplement: S5 Text — ALB-albumin; ALT-alanine aminotransferase; APTT-activated partial-thromboplastin time; AST-creatin phosphokinase; BUN-blood urea nitrogen; CK-creatin phosphokinase; CK-MB-creatinine kinase myocardial b fraction; sCr-serum creatinine; CRP-C reactive protein; Hgb-hemoglobin; LDH-lactate dehydrogenase; LYM%-lymphocyte percentage; MON%-monocyte percentage; MON-monocyte; NEU%-neutrophil percentage; NEU-neutrophil; PLT-platelet count; PT-partial-thromboplastin time; TB-total bilirubin; TT-thrombin time; WBC-white blood cell; LYM-lymphocyte. (DOCX) [file pntd.0010489.s010.docx]

**Funnel plots for laboratory parameters reported in at least five studies**

**S1 Fig. Funnel plot of ALB estimates**


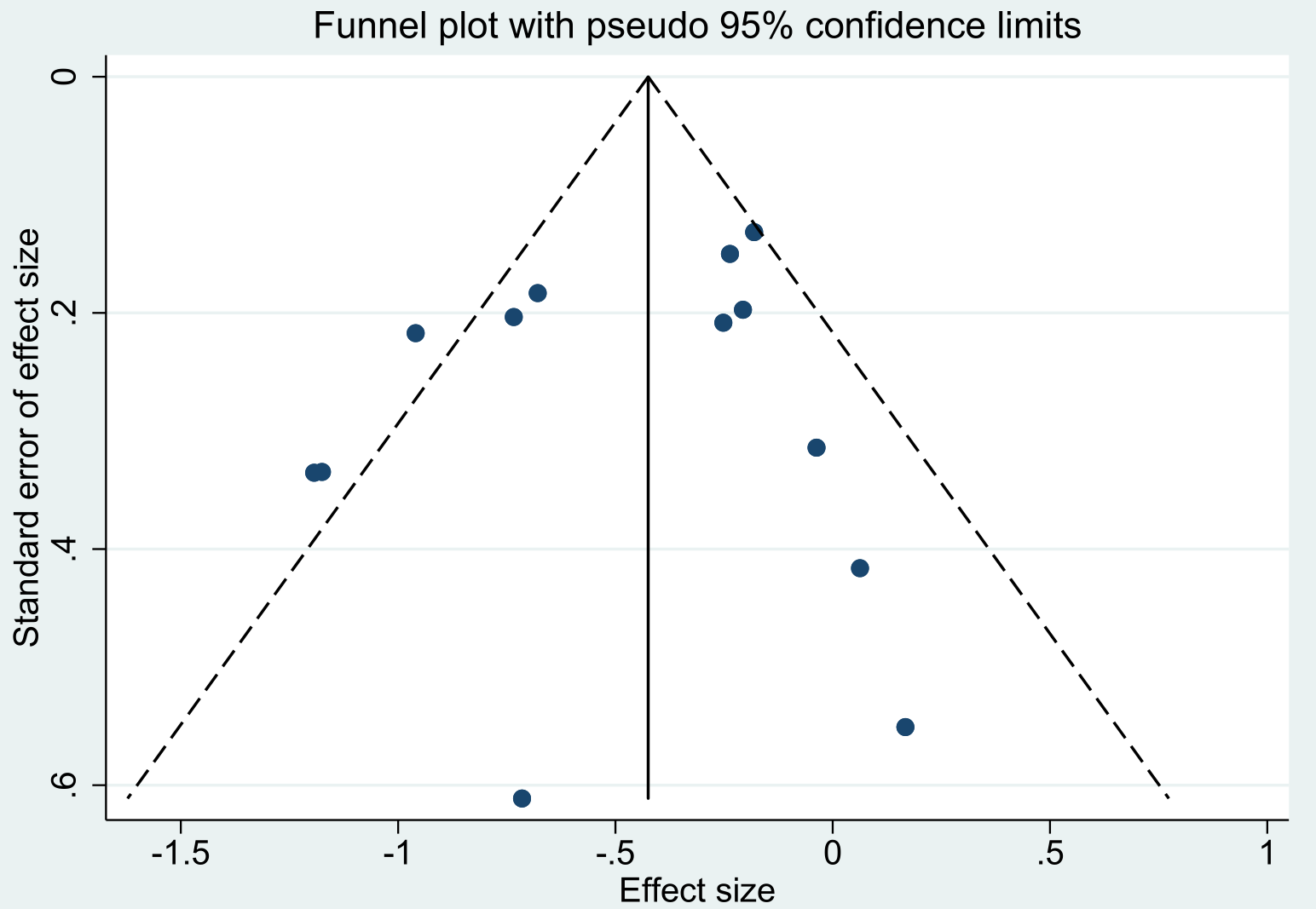


*Egger’s test: bias=-0.94; standard error=1.17; t=-0.80; p-value=0.440*

**S2 Fig. Funnel plot of ALT estimates**


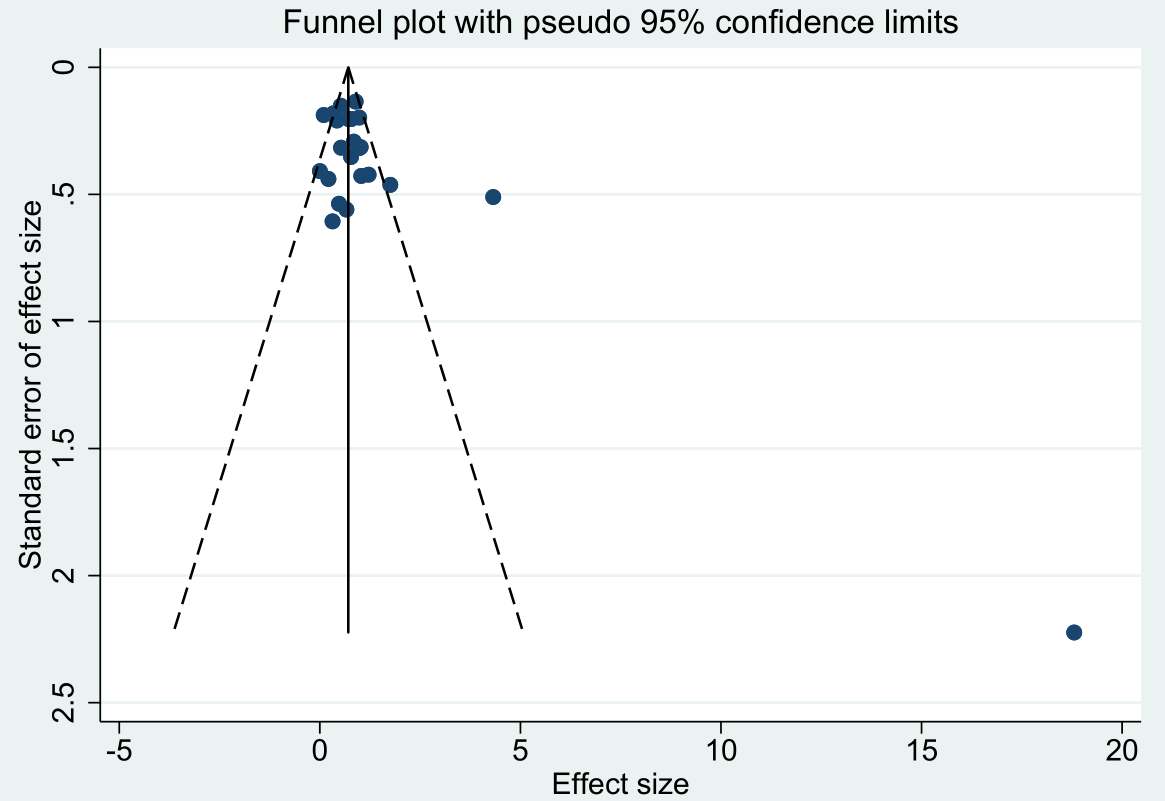


*Egger’s test: bias=2.73; standard error=1.11; t=2.47; p-value=0.022*

**S3 Fig. Funnel plot of APTT estimates**


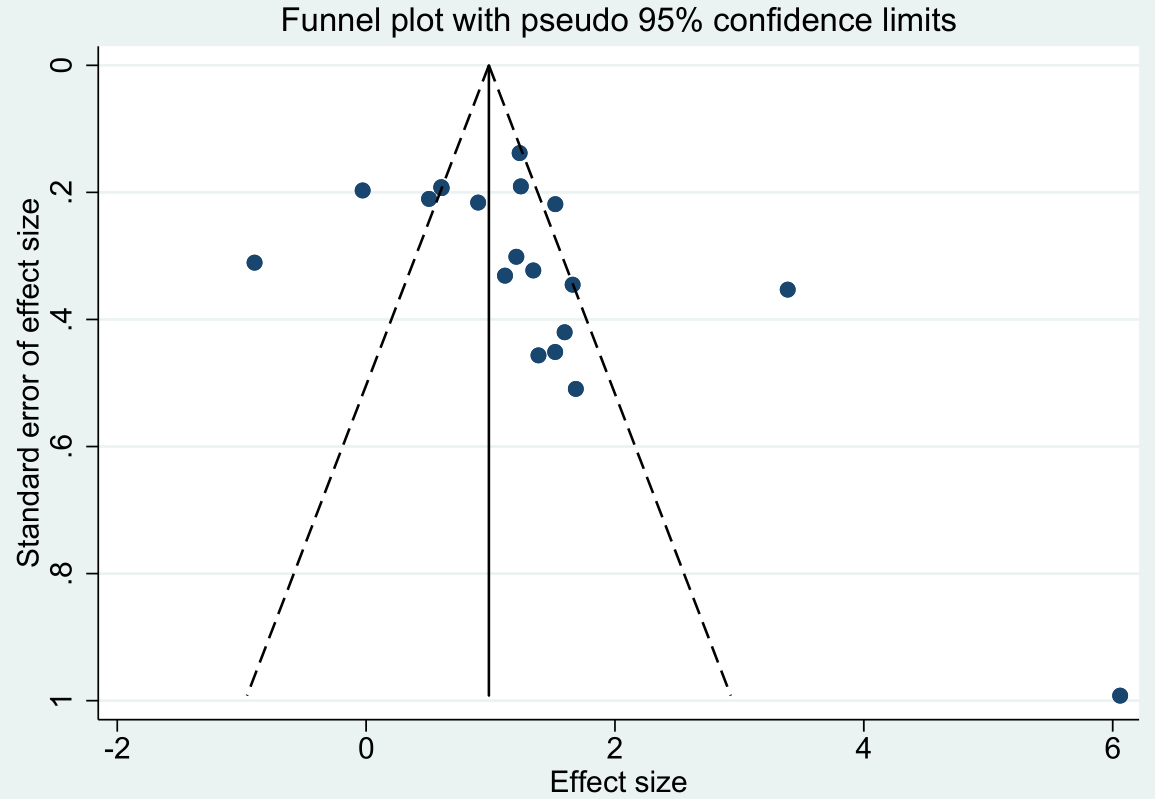


*Egger’s test: bias=3.19; standard error=1.77; t=1.81; p-value=0.088*

**S4 Fig. Funnel plot of AST estimates**


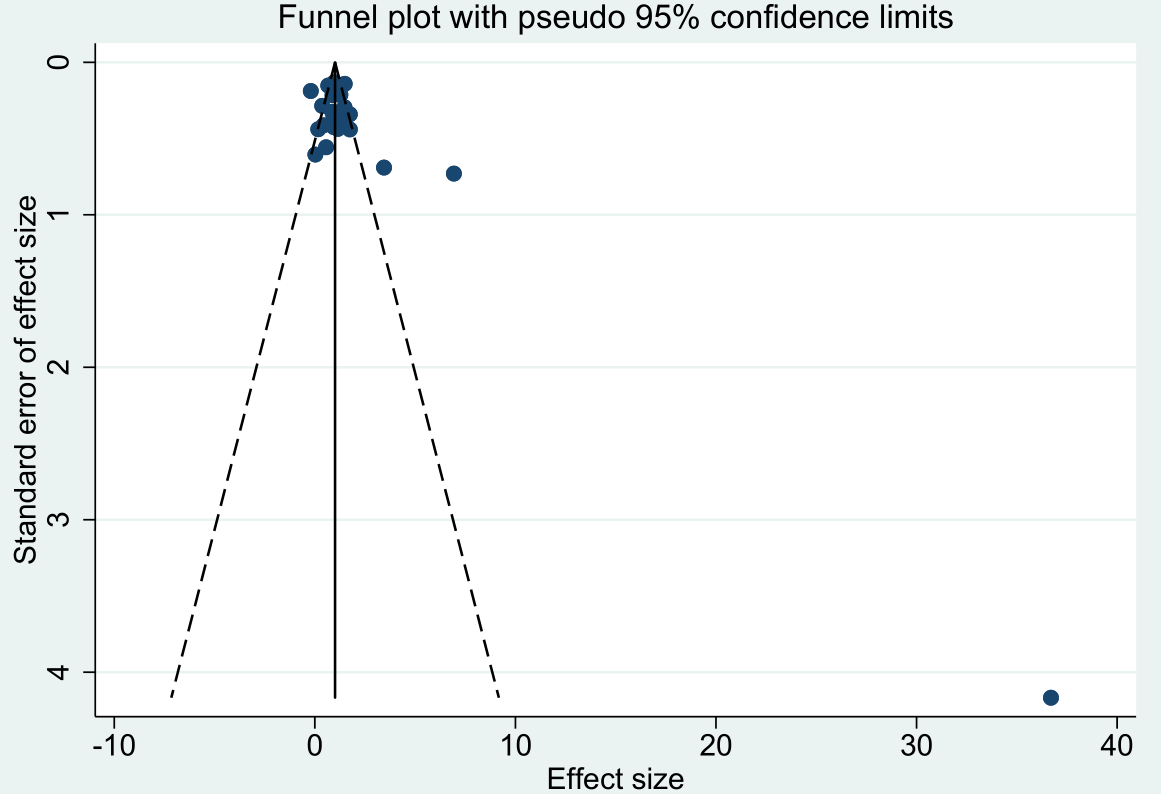


*Egger’s test: bias=2.65; standard error=1.17; t=2.26; p-value=0.033*

**S5 Fig. Funnel plot of BUN estimates**


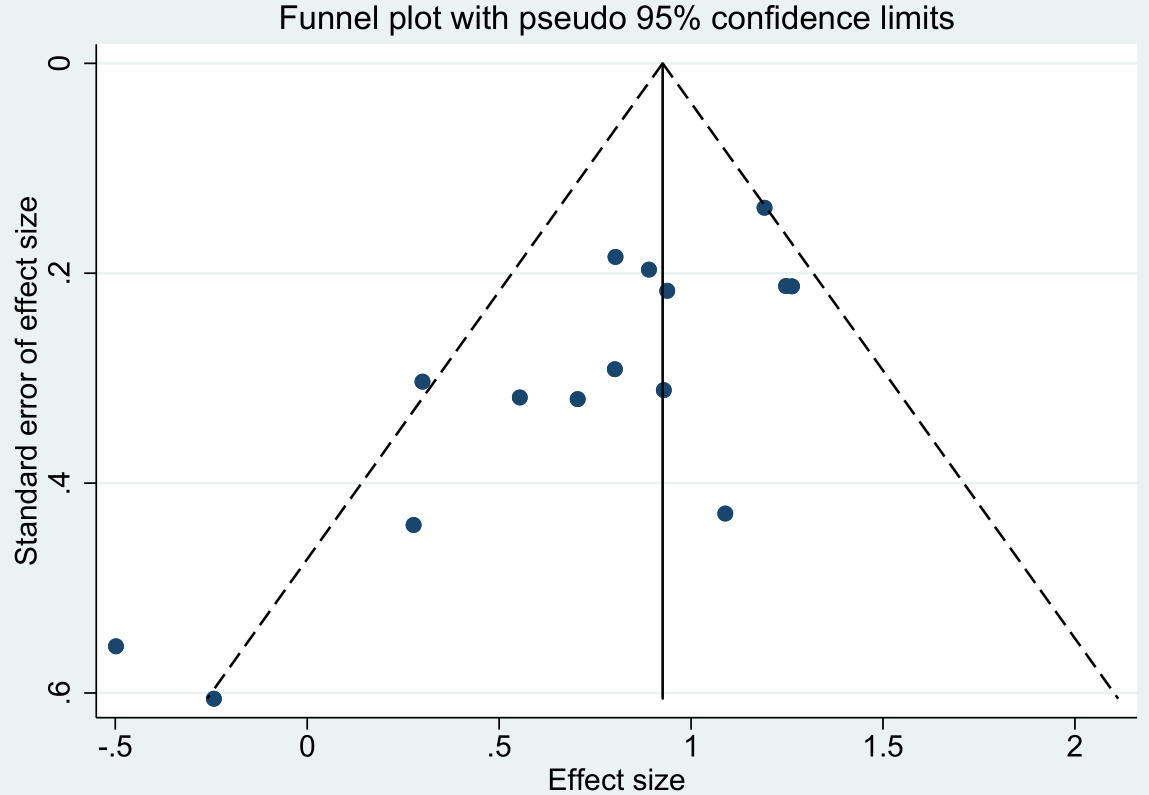


*Egger’s test: bias=-2.77; standard error=0.67; t=-4.11; p-value=0.001*

**S6 Fig. Funnel plot of CK estimates**


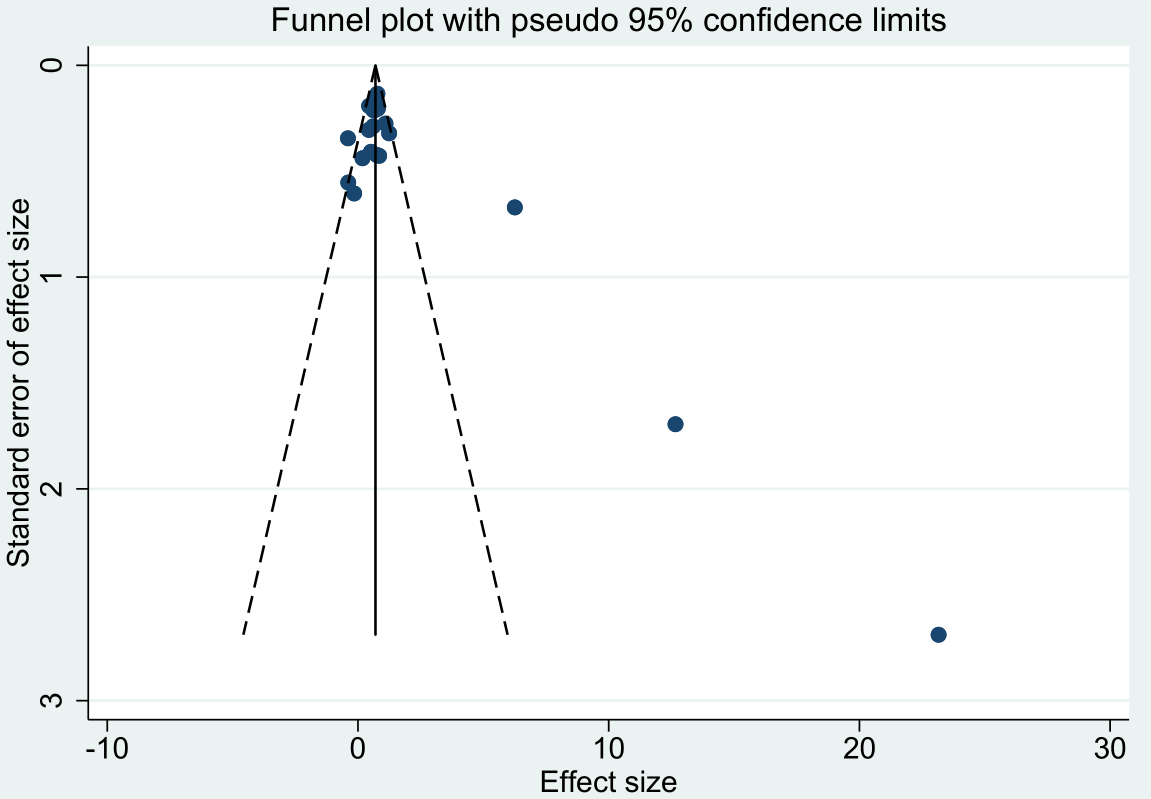


*Egger’s test: bias=3.32; standard error=1.29; t=2.57; p-value=0.018*

**S7 Fig. Funnel plot of CK-MB estimates**


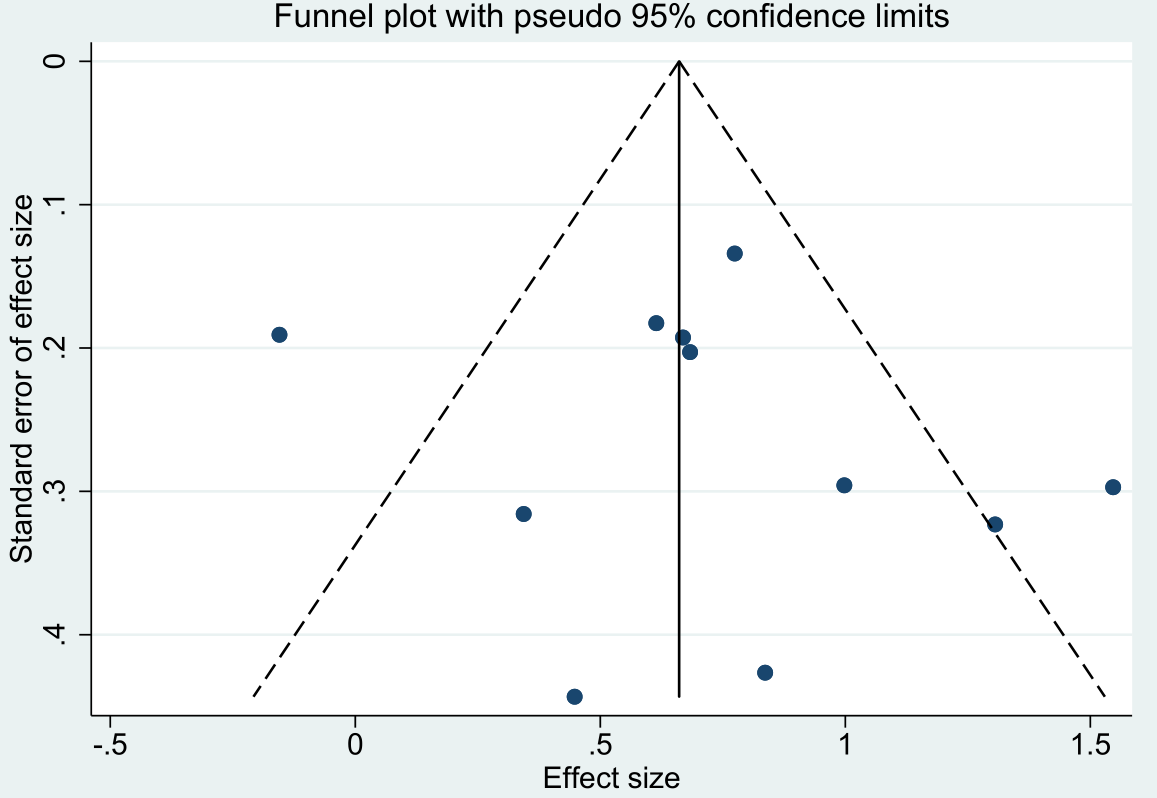


*Egger’s test: bias=1.16; standard error=1.68; t=0.69; p-value=0.508*

**S8 Fig*. Funnel plot of sCr estimates***


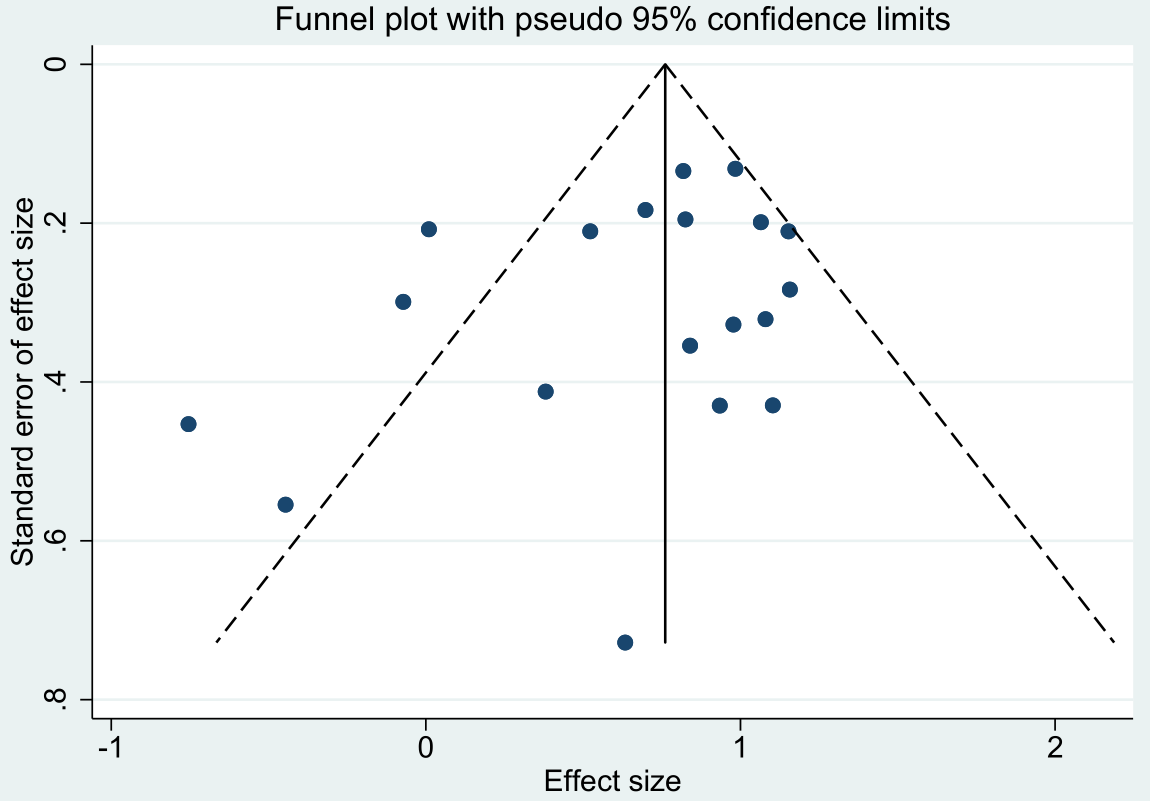


*Egger’s test: bias=-130; standard error=0.93; t=-1.41; p-value=0.178*

**S9 Fig. Funnel plot of CRP estimates**


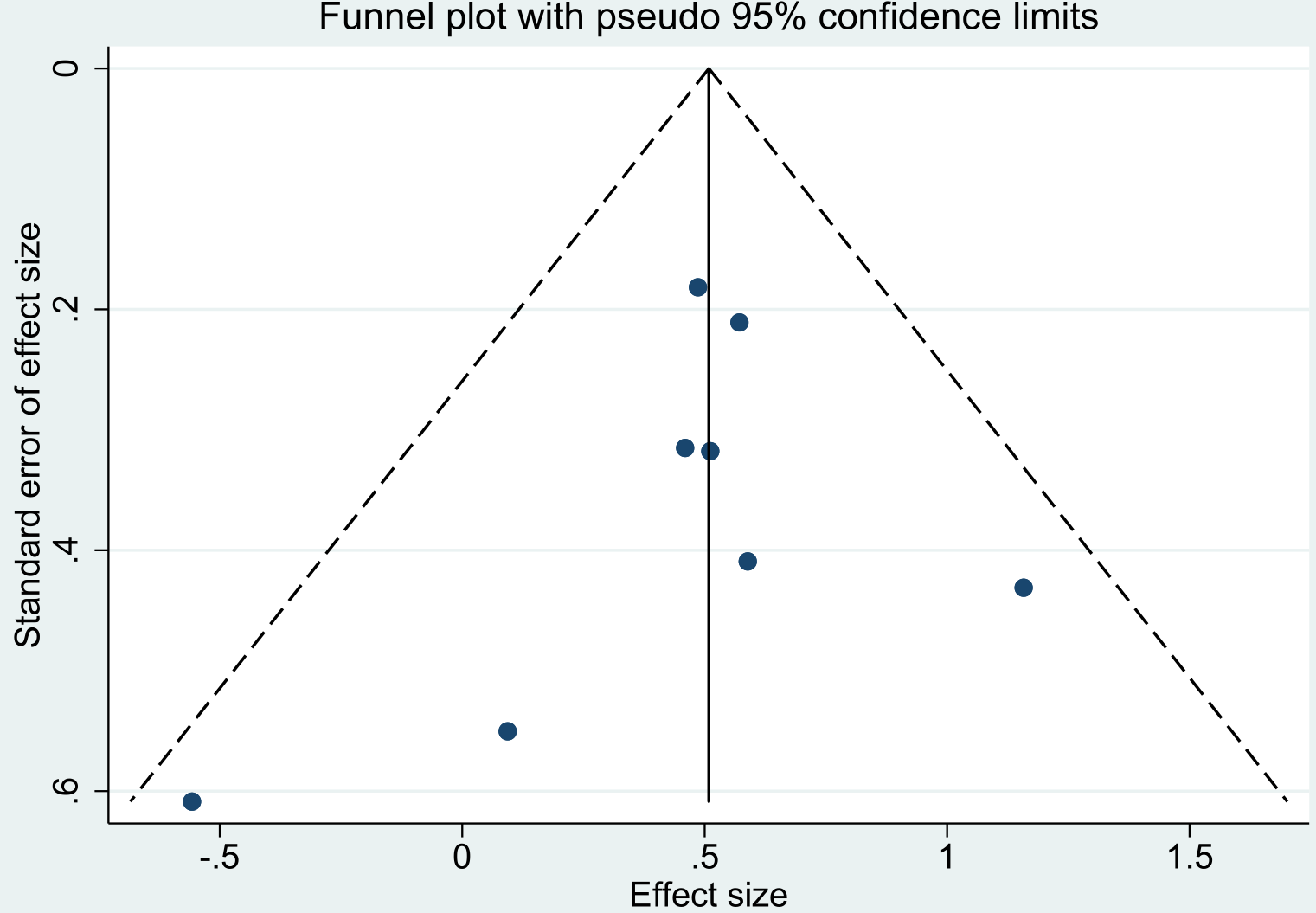


*Egger’s test: bias=-0.66; standard error=0.89; t=-0.74; p-value=0.488*

**S10 Fig. Funnel plot of Hgb estimates**


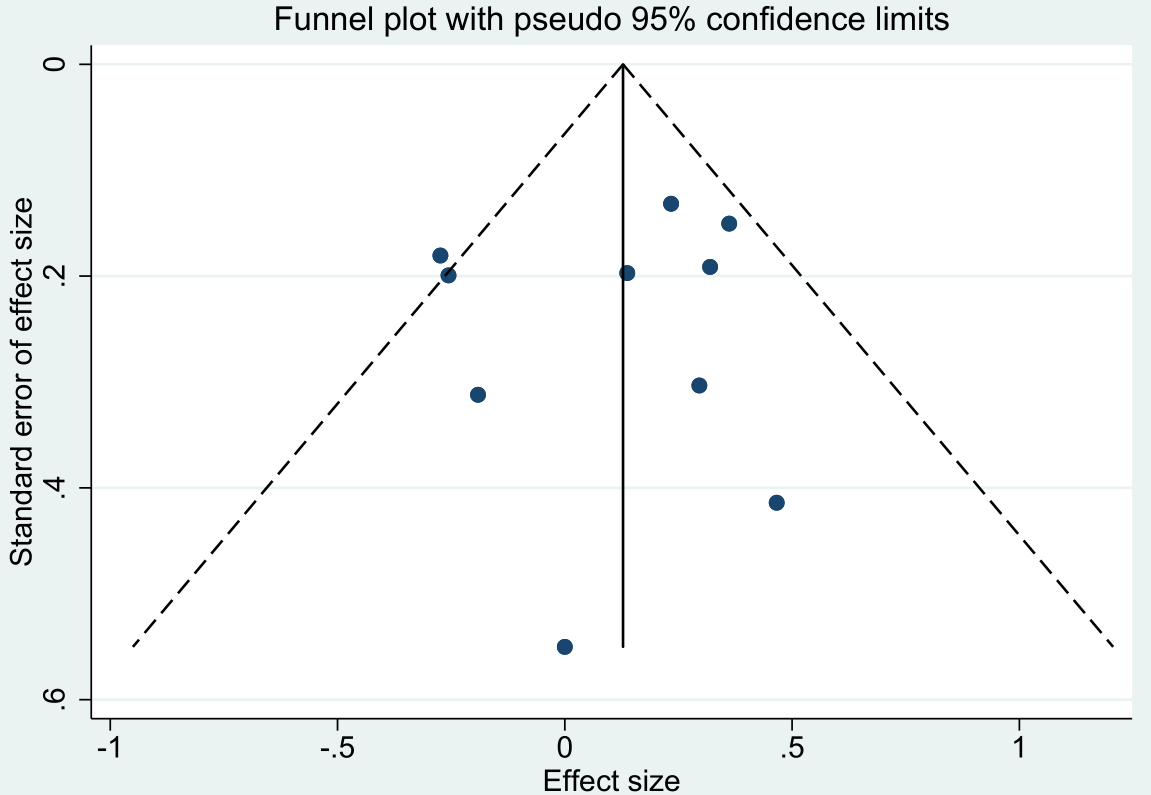


*Egger’s test: bias=-0.49; standard error=1.19; t=-0.41; p-value=0.689*

**S11 Fig. Funnel plot of K estimates**


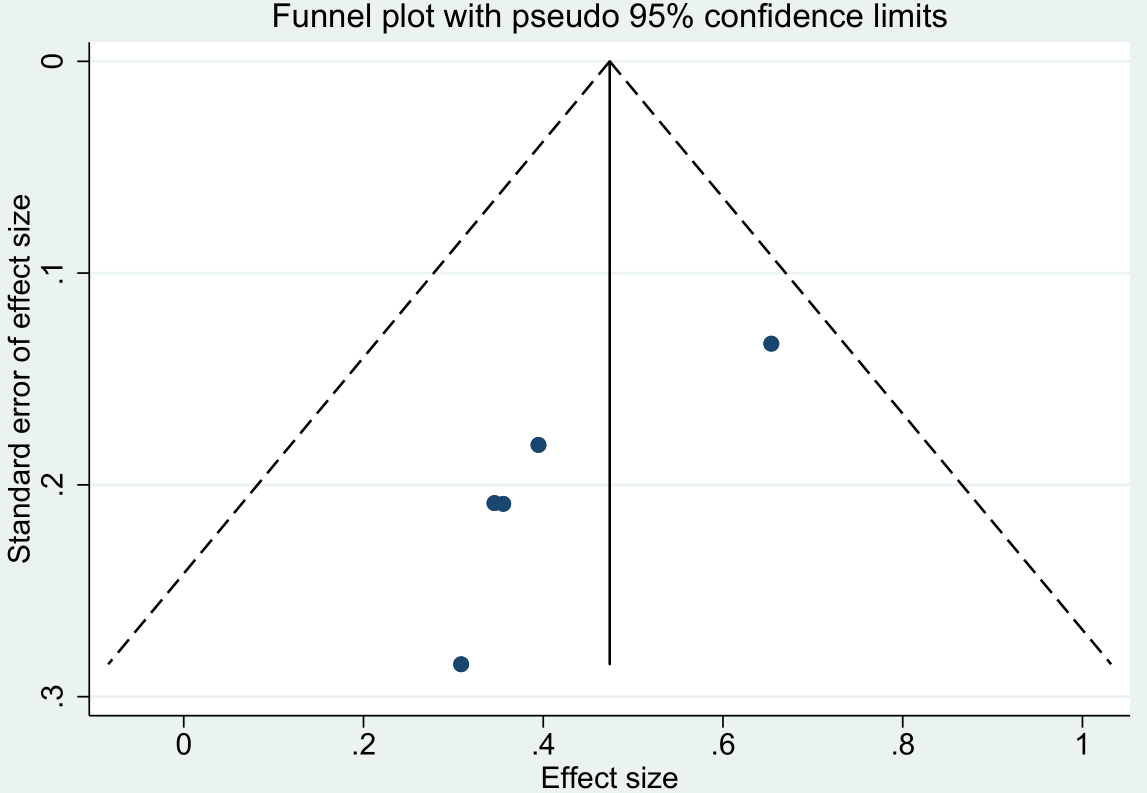


*Egger’s test: bias=-2.88; standard error=0.82; t=-3.51; p-value=0.039*

**S12 Fig*. Funnel plot of LDH estimates***


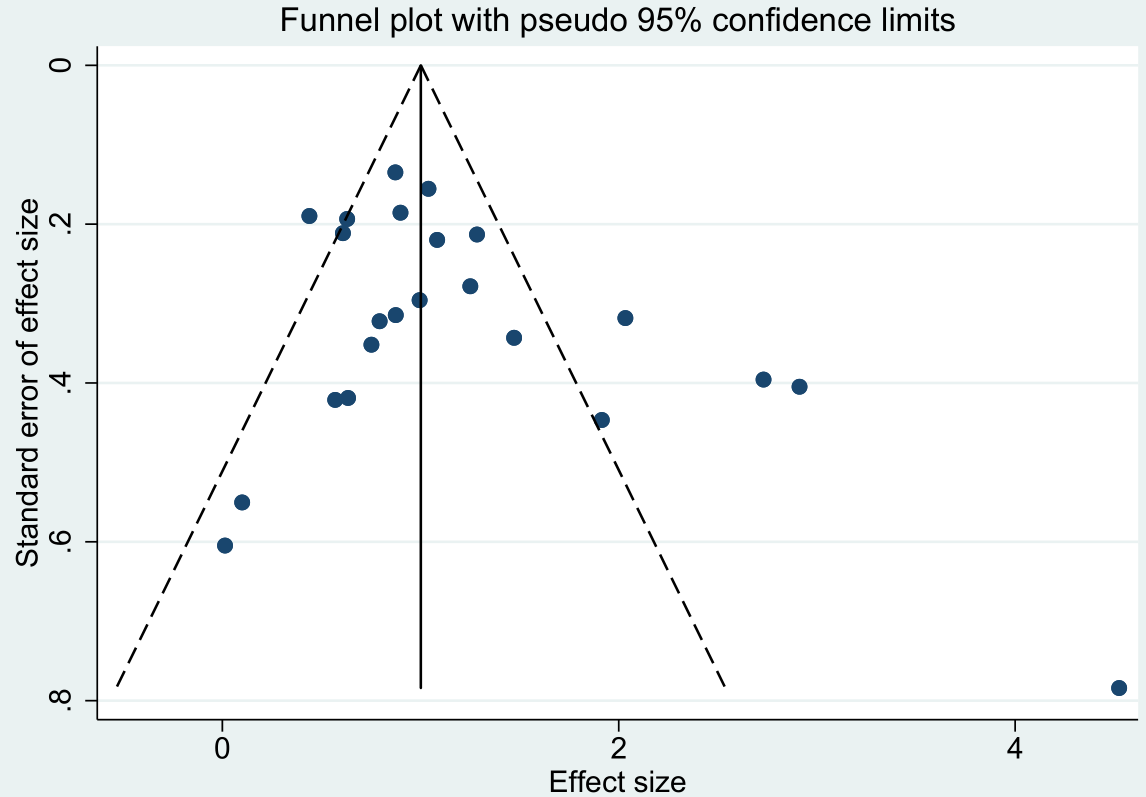


*Egger’s test: bias=2.17; standard error=1.08; t=2.01; p-value=0.058*

**S13 Fig. Funnel plot of viral load estimates**


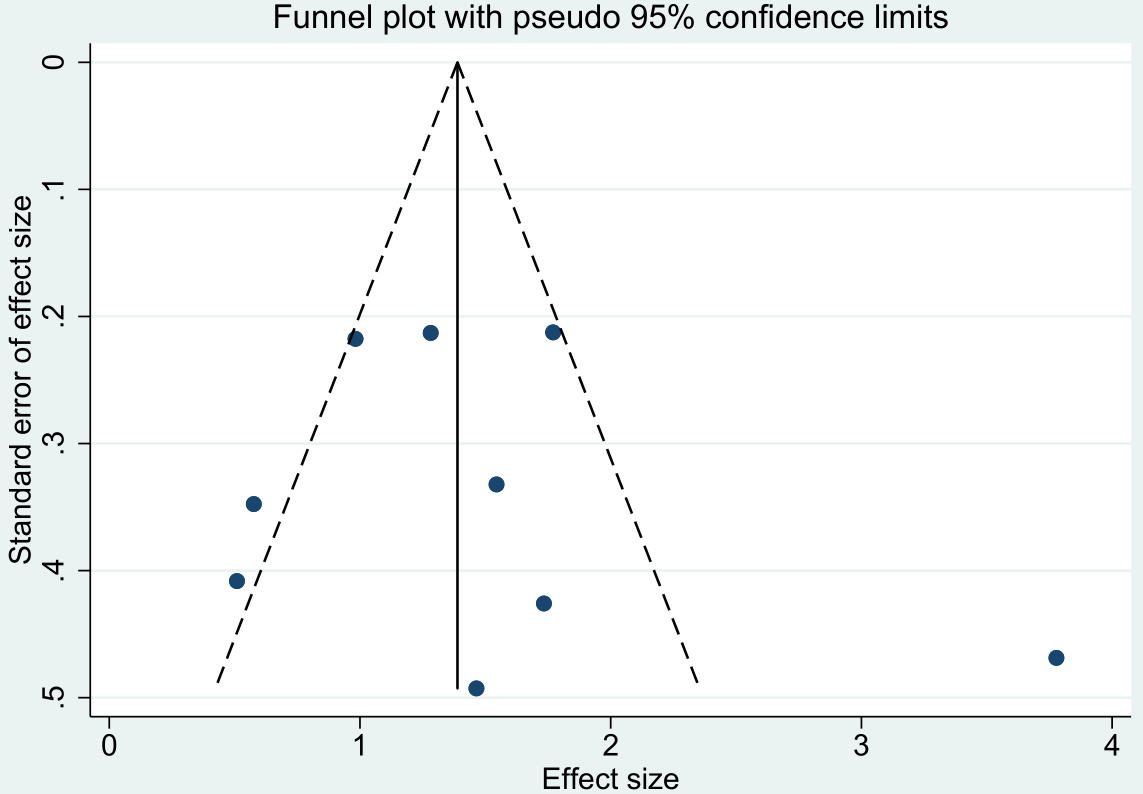


*Egger’s test: bias=1.51; standard error=2.08; t=0.72; p-value=0.490*

**S14 Fig. Funnel plot of LYM% estimates**


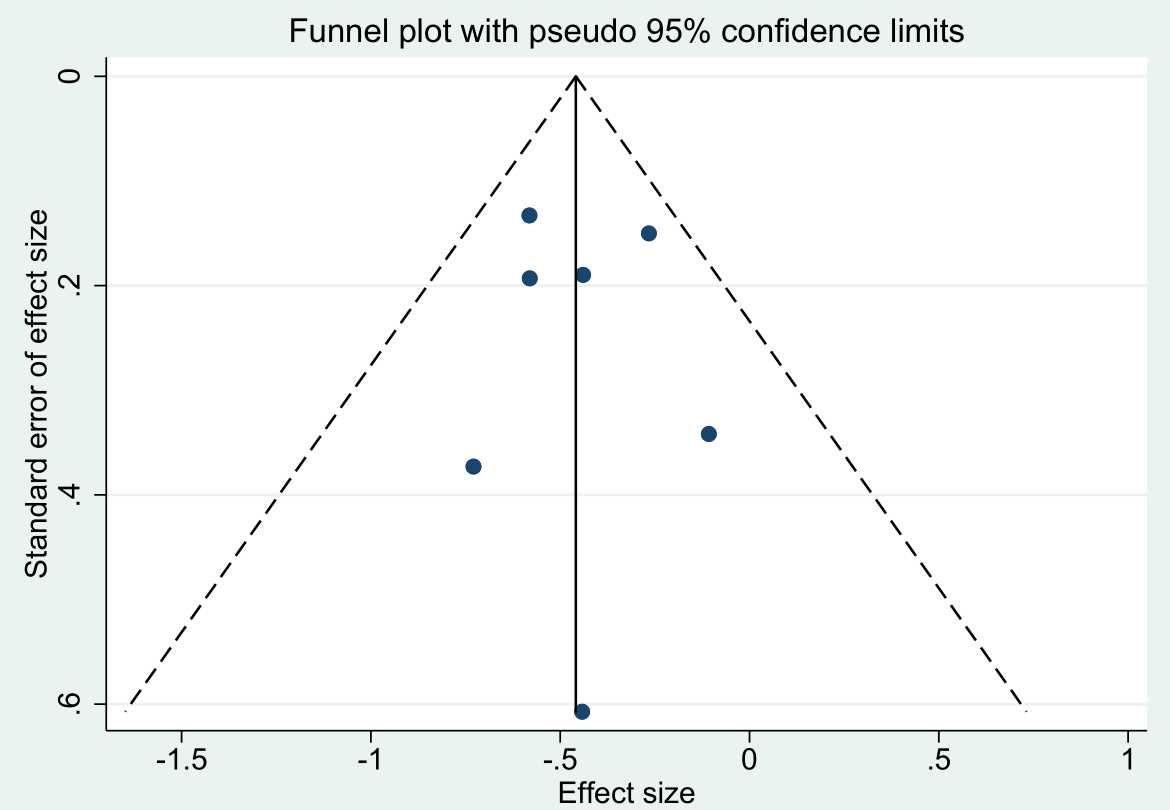


*Egger’s test: bias=0.14; standard error=0.88; t=0.16; p-value=0.878*

**S15 Fig. Funnel plot of MON% estimates**


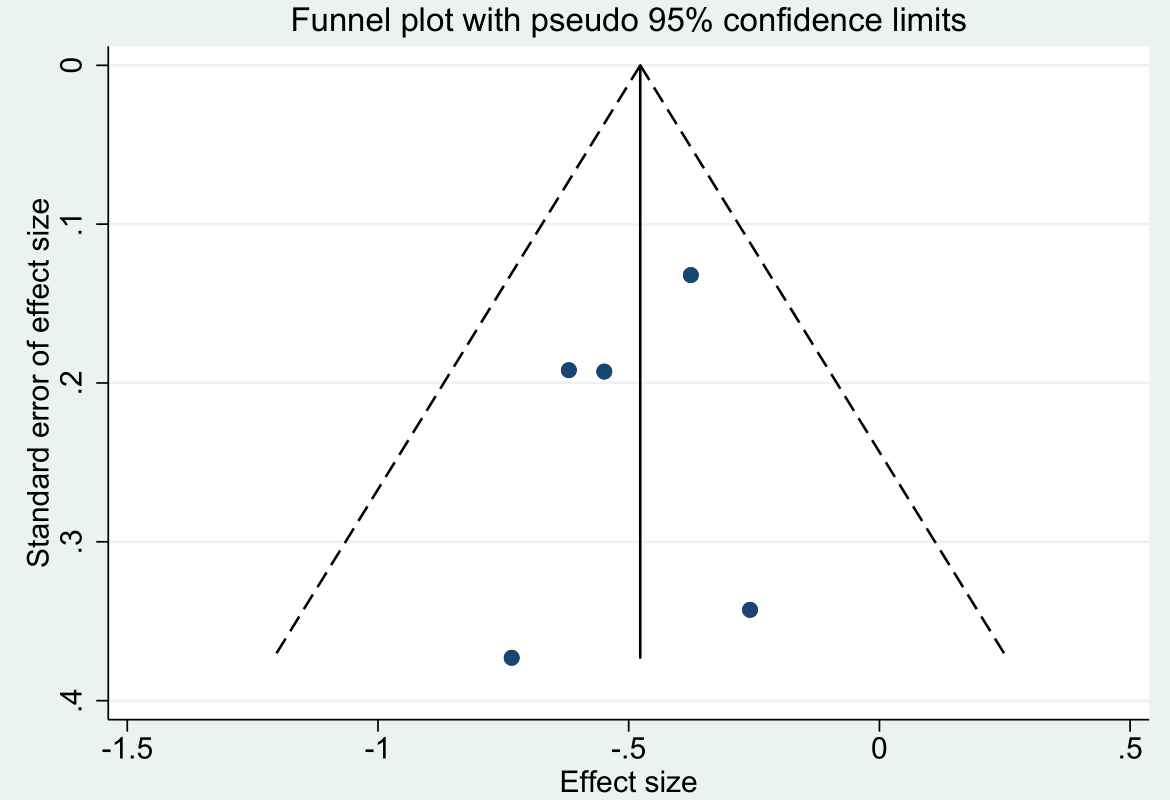


*Egger’s test: bias=-0.64; standard error=1.00; t=-0.63; p-value=0.571*

**S16 Fig. Funnel plot of MON estimates**


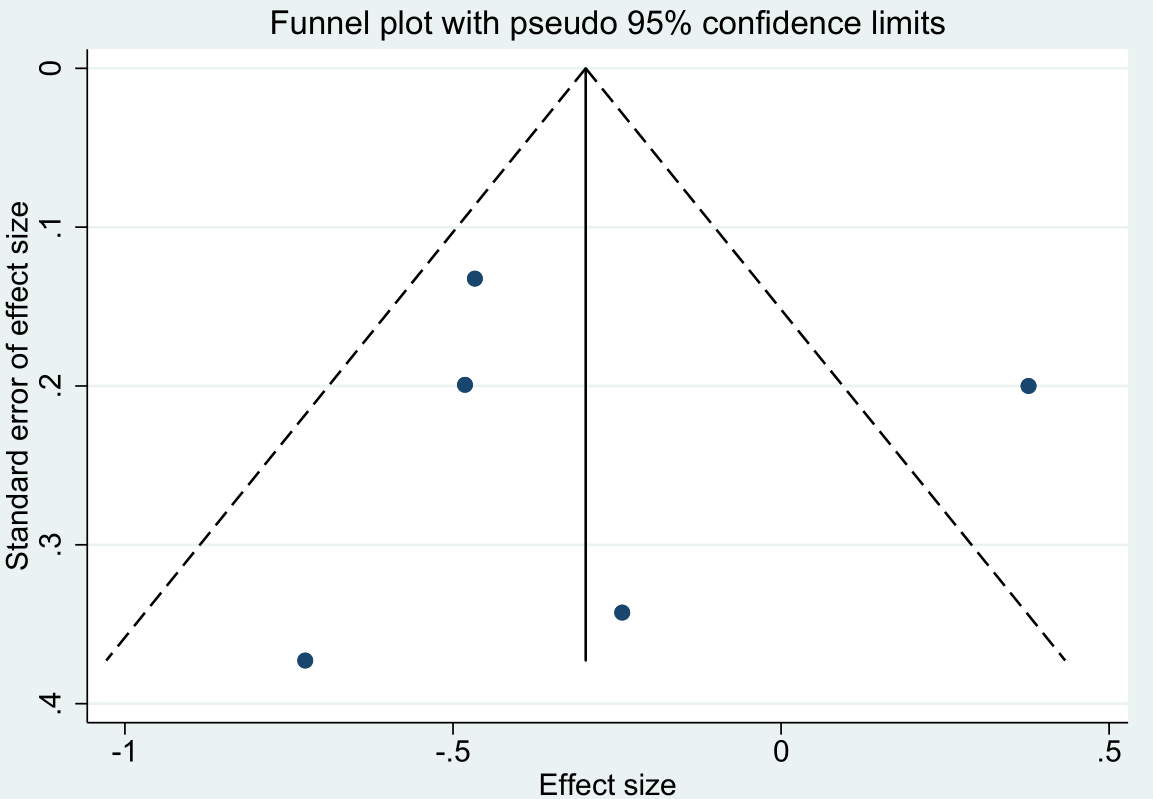


*Egger’s test: bias=0.30; standard error=2.83; t=0.11; p-value=0.922*

**S17 Fig. Funnel plot of Na estimates**


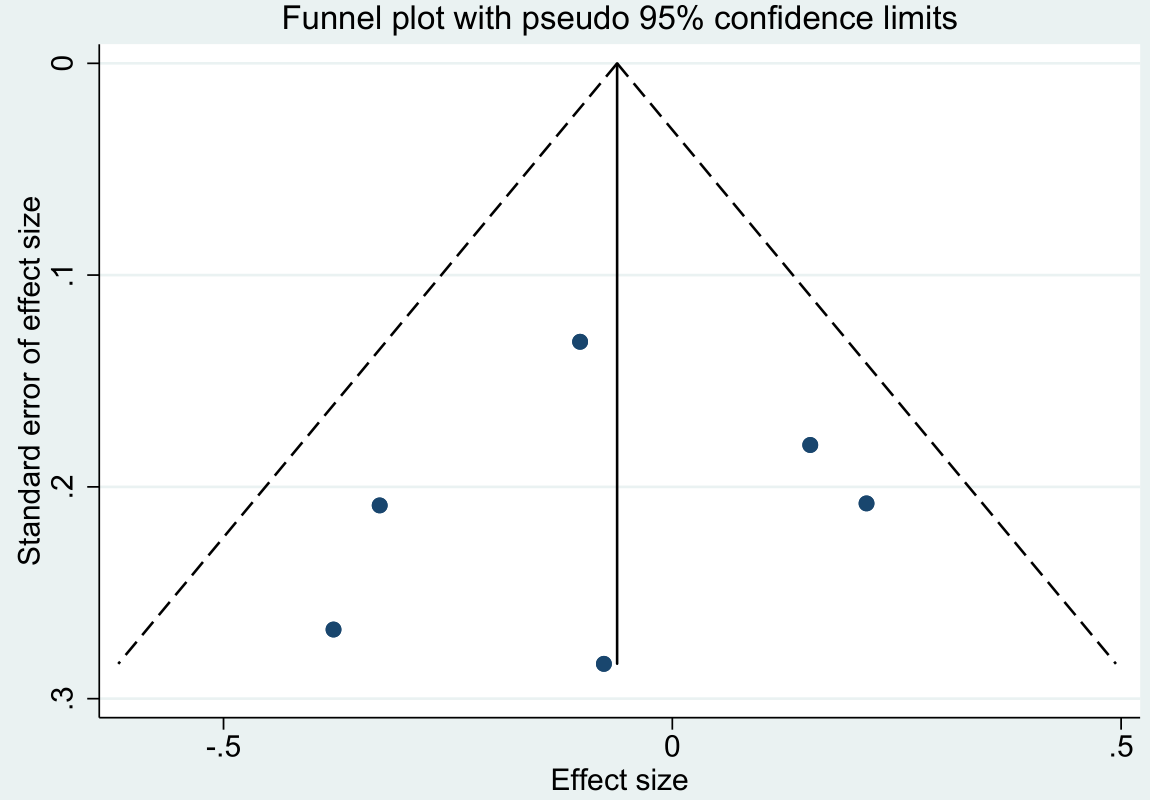


*Egger’s test: bias=-0.70; standard error=1.94; t=-0.36; p-value=0.738*

**S18 Fig. Funnel plot of NEU% estimates**


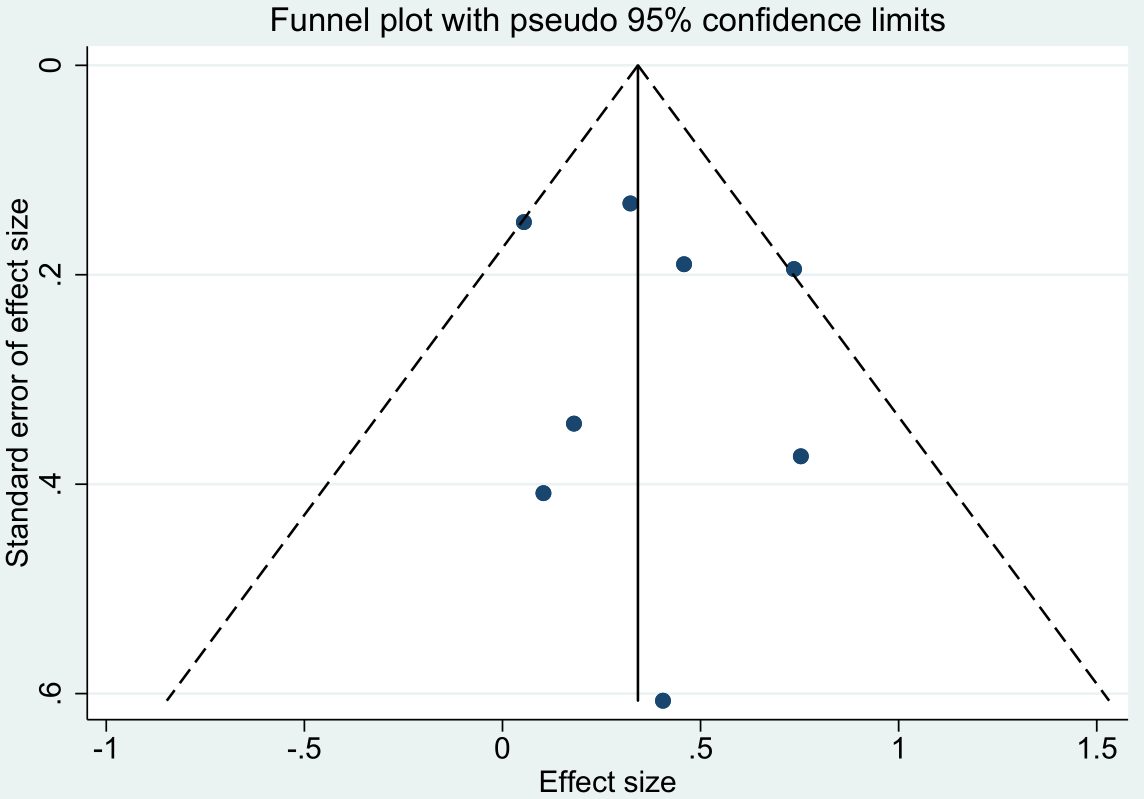


*Egger’s test: bias=0.49; standard error=1.05; t=0.47; p-value=0.656*

**S19 Fig. Funnel plot of NEU estimates**


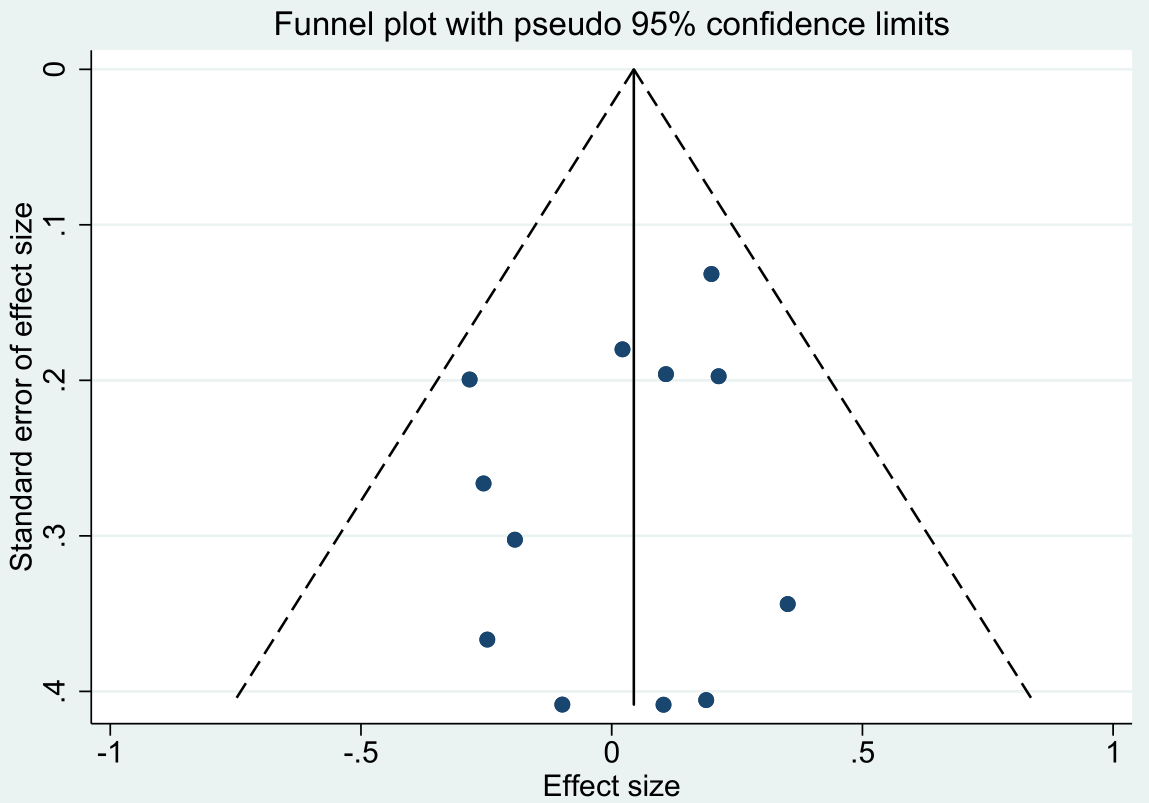


*Egger’s test: bias=-0.68; standard error=0.71; t=-0.97; p-value=0.356*

**S20 Fig. Funnel plot of PLT estimates**


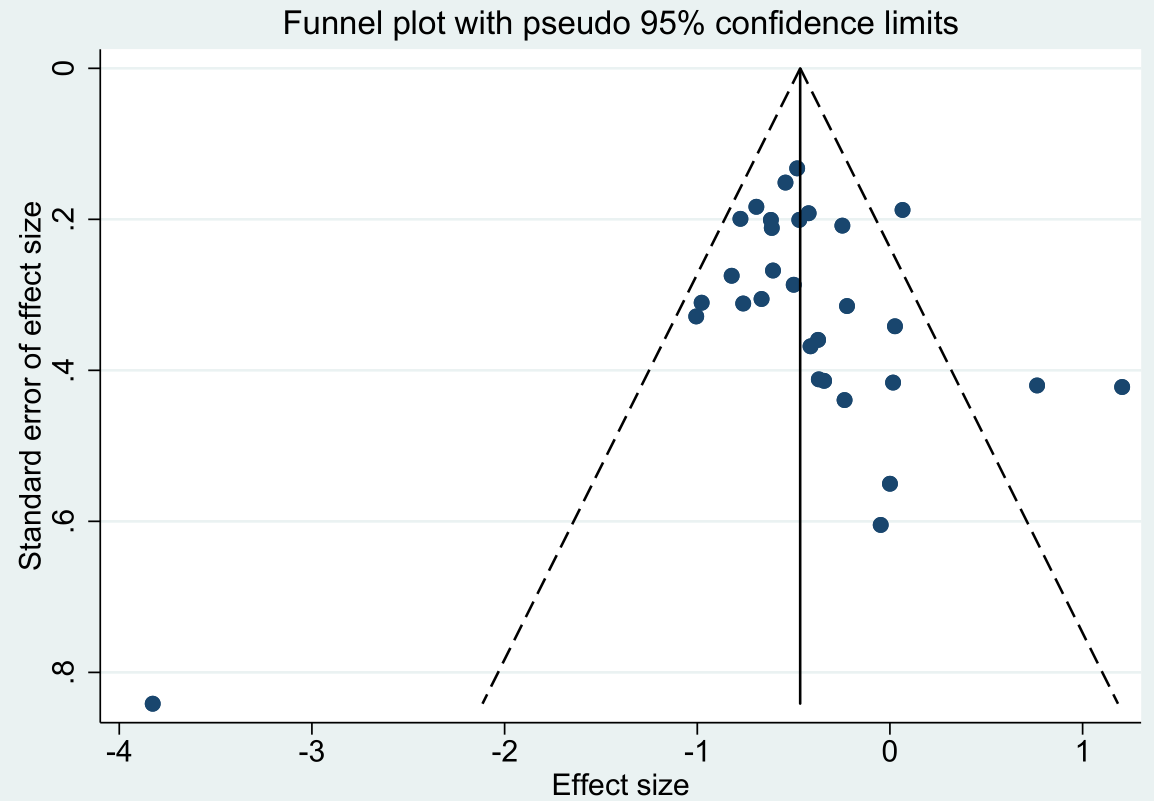


*Egger’s test: bias=0.37; standard error=0.75; t=0.50; p-value=0.622*

**S21 Fig. Funnel plot of PT estimates**


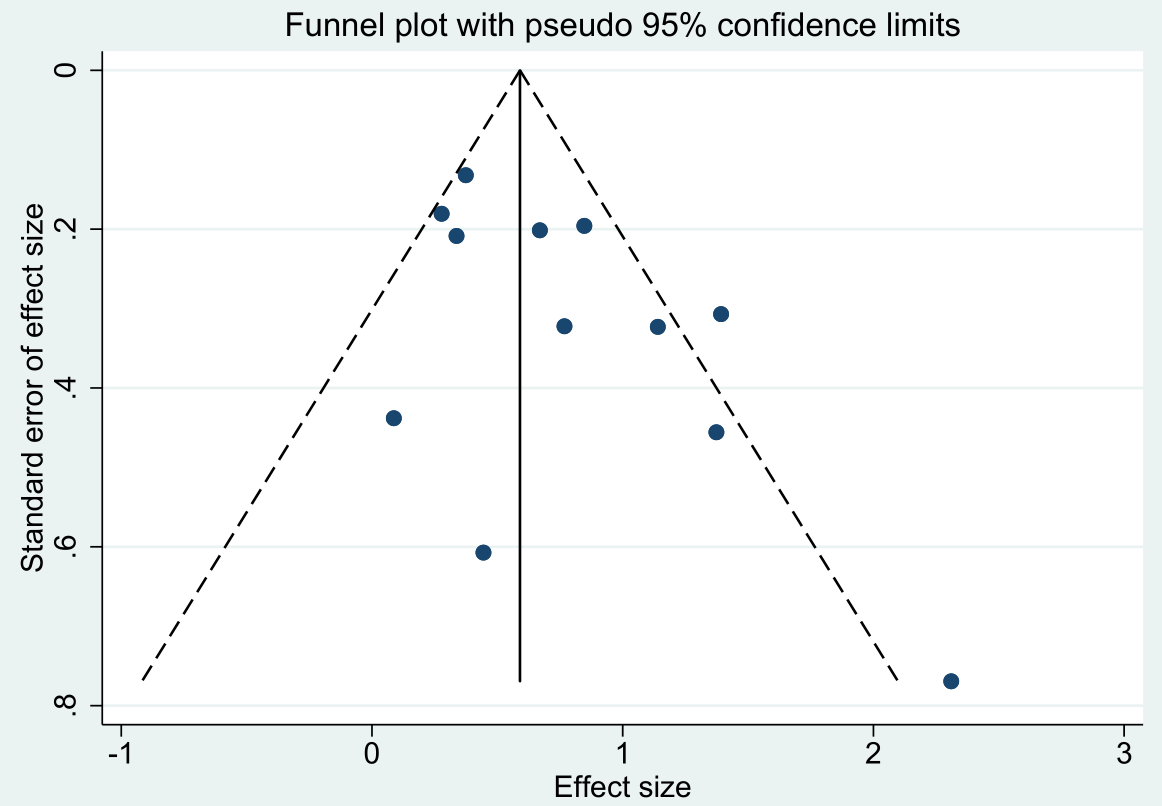


*Egger’s test: bias=2.04; standard error=0.92; t=2.21; p-value=0.052*

**S22 Fig. Funnel plot of TB estimates**


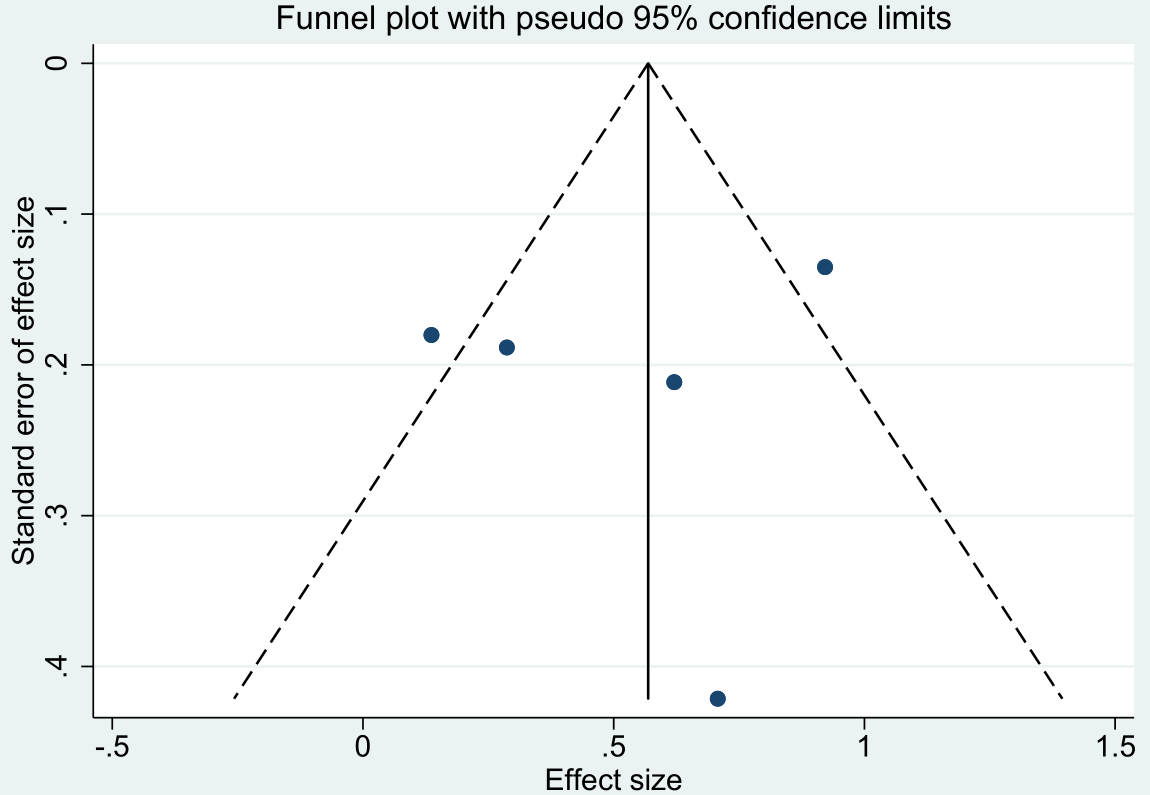


*Egger’s test: bias=-1.54; standard error=3.16; t=-0.49; p-value=0.660*

**S23 Fig. Funnel plot of TT estimates**


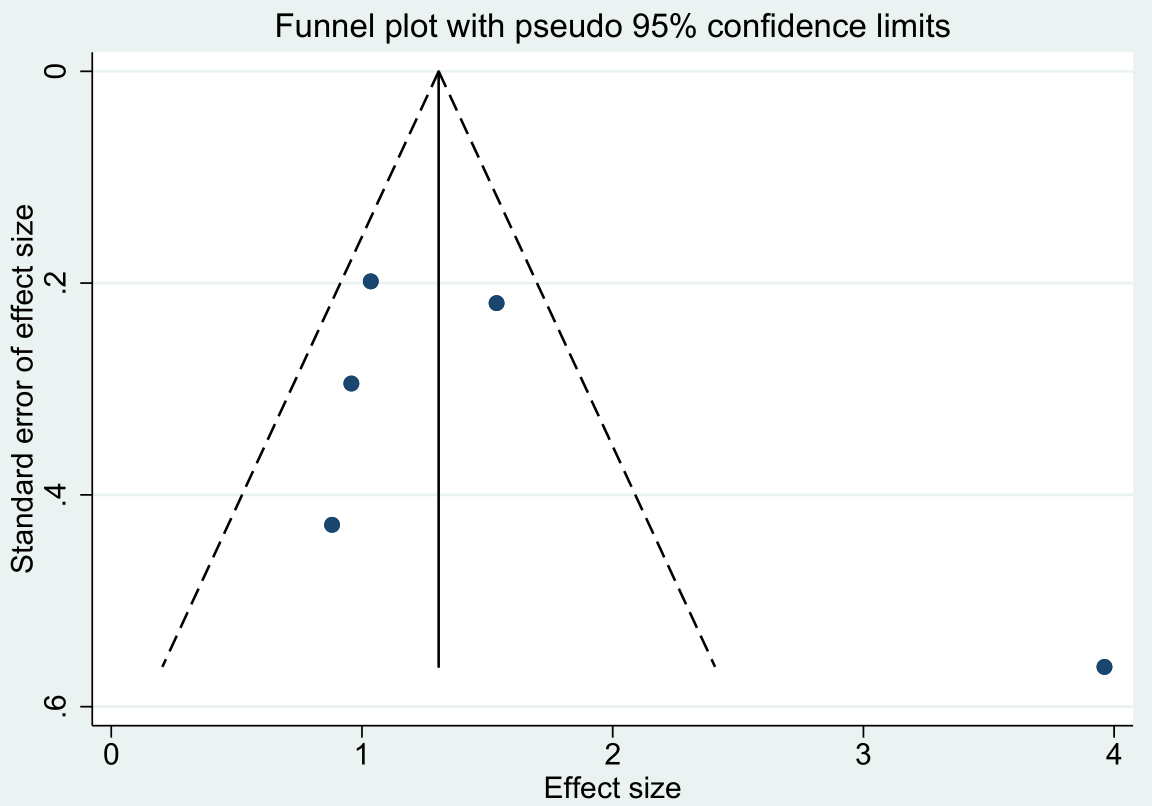


*Egger’s test: bias=3.80; standard error=3.29; t=1.15; p-value=0.333*

**S24 Fig. Funnel plot of WBC estimates**


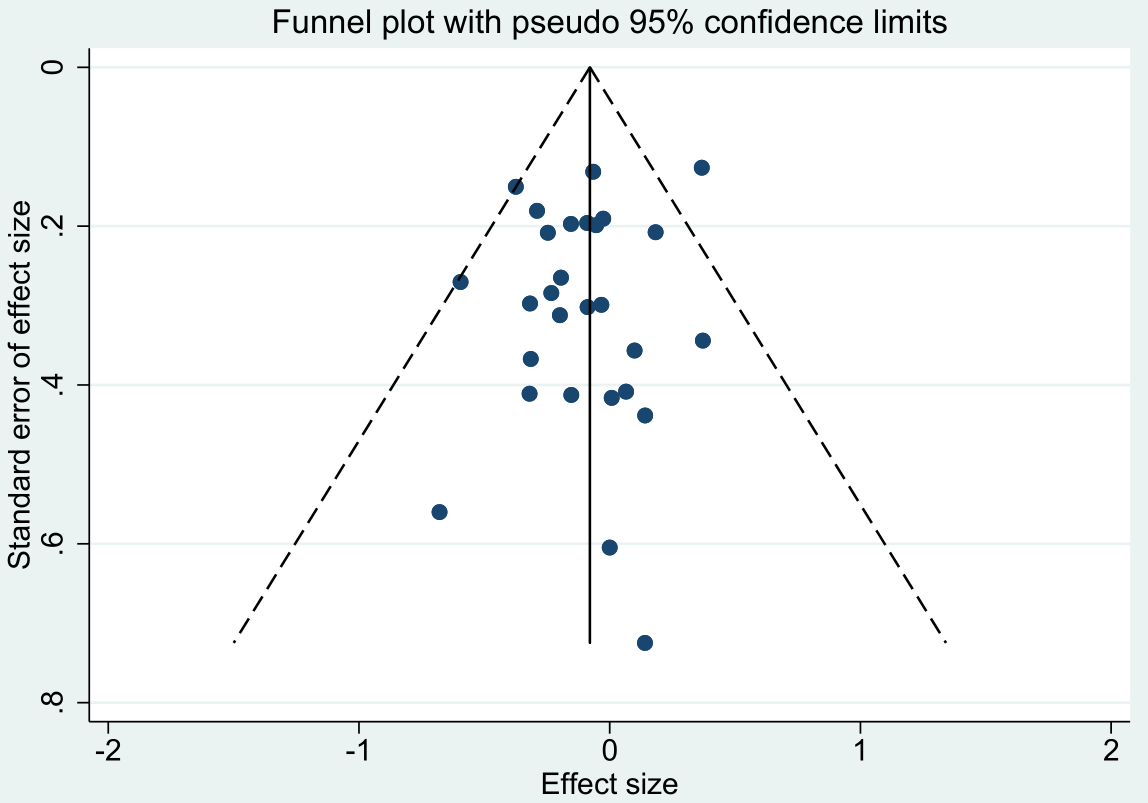


*Egger’s test: bias=-0.48; standard error=0.49; t=-0.98; p-value=0.336*

**S25 Fig. Funnel plot of LYM estimates**


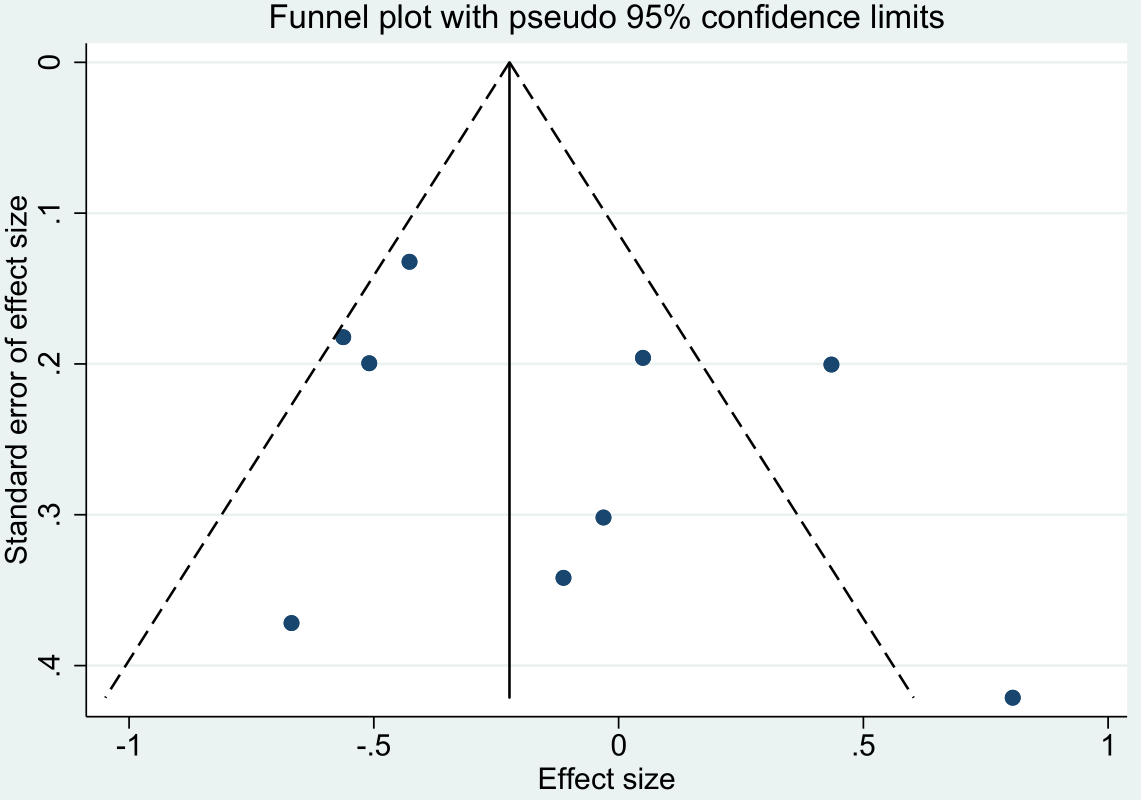


*Egger’s test: bias=1.94; standard error=1.83; t=1.06; p-value=0.325*
